# Quantitative Modeling of the Alternative Pathway of the Complement System

**DOI:** 10.1371/journal.pone.0152337

**Published:** 2016-03-31

**Authors:** Nehemiah Zewde, Ronald D. Gorham, Angel Dorado, Dimitrios Morikis

**Affiliations:** 1 Department of Bioengineering, University of California Riverside, Riverside, California, United States of America; 2 Department of Mechanical Engineering, University of California Riverside, Riverside, California, United States of America; University of California, Davis, UNITED STATES

## Abstract

The complement system is an integral part of innate immunity that detects and eliminates invading pathogens through a cascade of reactions. The destructive effects of the complement activation on host cells are inhibited through versatile regulators that are present in plasma and bound to membranes. Impairment in the capacity of these regulators to function in the proper manner results in autoimmune diseases. To better understand the delicate balance between complement activation and regulation, we have developed a comprehensive quantitative model of the alternative pathway. Our model incorporates a system of ordinary differential equations that describes the dynamics of the four steps of the alternative pathway under physiological conditions: (i) initiation (fluid phase), (ii) amplification (surfaces), (iii) termination (pathogen), and (iv) regulation (host cell and fluid phase). We have examined complement activation and regulation on different surfaces, using the cellular dimensions of a characteristic bacterium (*E*. *coli*) and host cell (human erythrocyte). In addition, we have incorporated neutrophil-secreted properdin into the model highlighting the cross talk of neutrophils with the alternative pathway in coordinating innate immunity. Our study yields a series of time-dependent response data for all alternative pathway proteins, fragments, and complexes. We demonstrate the robustness of alternative pathway on the surface of pathogens in which complement components were able to saturate the entire region in about 54 minutes, while occupying less than one percent on host cells at the same time period. Our model reveals that tight regulation of complement starts in fluid phase in which propagation of the alternative pathway was inhibited through the dismantlement of fluid phase convertases. Our model also depicts the intricate role that properdin released from neutrophils plays in initiating and propagating the alternative pathway during bacterial infection.

## Introduction

**Fig 1:**
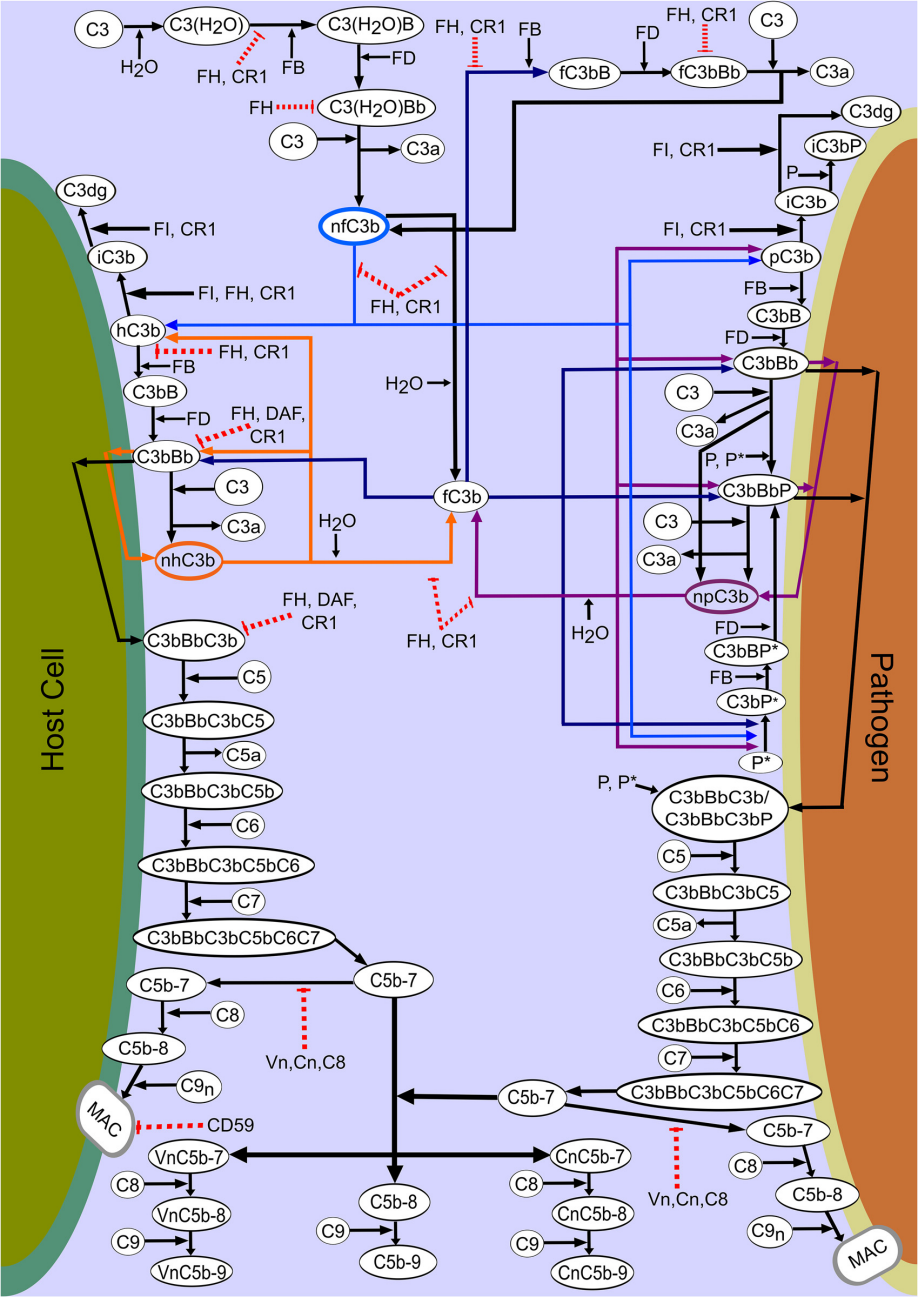
The biochemical reactions of the alternative pathway. Fluid state activation of the alternative pathway leads to C3b that can indiscriminately bind to host or pathogen surface. On the right (pathogen), complement activity propagates without the disruption of regulatory proteins. On the left (host), plasma proteins in conjunction with surface-bound regulators actively inhibit complement propagation by disrupting complement interaction at different points of the alternative pathway. Abbreviations: C3, complement component 3; C3(H_2_O), thioester-hydrolyzed form of C3; C3(H_2_O)Bb, initial C3 convertase of AP; C3a, anaphylatoxin fragment from C3; C3b, activated fragment from C3; iC3b, inactivated C3b; nfC3b, nascent fluid phase C3b; nhC3b, nascent host C3b; npC3b, nascent pathogen C3b; fC3b, fluid phase C3b; fC3bBb, fluid phase C3 convertase, C3bBb, C3 convertase; C3bBbP, C3 convertase with properdin; C3bBbP*, C3 convertase with properdin* released from neutrophils; C5, complement component 5; C5a anaphylatoxin fragment from C5; C5b, activated fragment from C5; C3bBbC3b, C5 convertase; FB, Factor B; FD, Factor D; FI, Factor I; CR1, Complement Receptor 1; DAF, Decay Accelerating Factor; Vn, vitronectin; Cn, clusterin; CD59, Protectin. Lines and colors: solid lines with an arrow tip denote activation and dashed lines with a straight tip denote inhibition. Red line color denotes inhibition. The rest of line colors denote clusters of reactions involving different C3b molecules, i.e. nhC3b (orange), nfC3b (light blue), npC3b (purple), fC3b (dark blue).

The complement system is one of our primary defense mechanisms that plays a vital role in coordinating immune responses. Complement function and regulation are induced by more than 30 distinct proteins present in plasma and on cell surfaces. The soluble proteins normally circulate as inactive precursors but once stimulated initiate cascades of biochemical reactions that propagate the activation of the complement system through three distinct pathways, the classical (CP), lectin (LP), and alternative (AP) pathways. The activation of these pathways leads to recognition and elimination of invading pathogens, recruitment of adaptive immunity, and facilitation of the removal of apoptotic cells [1–4]. Despite having similar roles, all three pathways have different sources for activation. The classical pathway is triggered by C1q binding to antigen-bound antibody complexes and also by direct attachment of C1q to pathogen surfaces. This leads to the production of C4b and C2a, which together form the C3 cleaving enzyme of the classical pathway, C4bC2a, called C3 convertase. Upon enzymatic cleavage, C3 is converted to the anaphylactic peptide C3a and opsonin C3b, which covalently attaches to cell surfaces via a thioester bond. The lectin pathway is activated by binding of mannose-binding lectin (MBL) with carbohydrate structures on bacterial and viral surfaces. This pathway is homologous to the classical pathway since the lectin pathway initiates complement cascade using MBL, a protein that is similar to C1q. The lectin pathway also forms the C3 convertase C4bC2a. Finally, the alternative pathway activation starts in the fluid phase by the spontaneous hydrolysis of the internal thioester bond of C3 to form a molecule known as hydrolyzed C3, C3(H_2_O). This molecule follows a cascade of reactions that involves complement proteins Factor B and Factor D to form the initial C3 convertase of the alternative pathway, C3(H_2_O)Bb. This protease complex like its counterparts in the classical and lectin pathway also generates C3a and C3b fragments by cleaving C3. Once C3b attaches to the surface of the pathogen it plays a crucial role in initiating a robust AP activation that results in an amplification loop. The cleavage of C3 is the convergence point of all three activation pathways and the initiation point of the common pathway towards the formation of the membrane attack complex that produces a pore on the surface of pathogens to cause lysis. Fig 1 shows the set of biochemical reactions involved in the alternative pathway through its initiation in the fluid phase, continued propagation on pathogen surface, and regulation on host cell.

Due to the detrimental effects of the complement system, a delicate balance must be kept between activation and inhibition to prevent host cell damage. The complement system is regulated through the actions of complement control proteins (CCPs), which are present in blood plasma and on the surfaces of host cells. In cases of impaired complement regulation, inadequate activation (or hyper-regulation) may cause susceptibility to infection, whereas excessive complement activation (or hypo-regulation) can harm host cells and tissues, resulting in autoimmune and inflammatory diseases. For instance, systemic lupus erythematosus (SLE) [2,5,6], atypical hemolytic uremic syndrome (aHUS) [5,7,8], paroxysmal nocturnal hemoglobinuria (PNH) [2,5], and age related macular degeneration (AMD) [9,10] are all related to the malfunction of the complement regulation.

The complexity of the interplay between complement function and regulation makes mathematical modeling of the complement pathways an indispensable tool in understanding the dynamics of the biochemical reactions during activation and inhibition. Differential equations can be used to model the effects of complement proteins under pathological situations, such as protein deficiencies, sequence deletions, or amino acid mutations, and compare their effects on the global dynamics of the system. The goal of the present work is to mathematically model and interpret the dynamics of the biochemical reactions in the alternative pathway of the complement system during infections (pathogens) and homeostasis (host cells).

Earlier studies of mathematical modeling of the complement system began with the classical pathway in which the dynamics of the system were assumed to be linear [11]. A subsequent model was proposed in which the dynamics were assumed to be non-linear and also included the alternative pathway [12]. A later work [13] was then performed on the classical and lectin pathways that also added an experimental study. Our study is the first to incorporate a comprehensive mathematical model that highlights the four time-dependent dynamic steps of the alternative pathway: (i) initiation (fluid phase), (ii) amplification (surfaces), (iii) termination (pathogen), and (iv) regulation (host cell and fluid state). We also have incorporated representative bacteria (*E*. *coli*) and representative host cells (human erythrocytes) to account for complement activation and regulation on different surfaces. In addition, our proposed model incorporates all the known complement regulatory proteins that act in the fluid phase, host cell membranes, and also exogenously on bacteria. Finally, we included neutrophil-secreted properdin in our dynamic system because neutrophils, once stimulated, they have the ability to release properdin (P*), the only known positive complement regulator that has been shown to bind to surfaces and initiate complement activation.

## Results

### Activation and propagation

**Fig 2:**
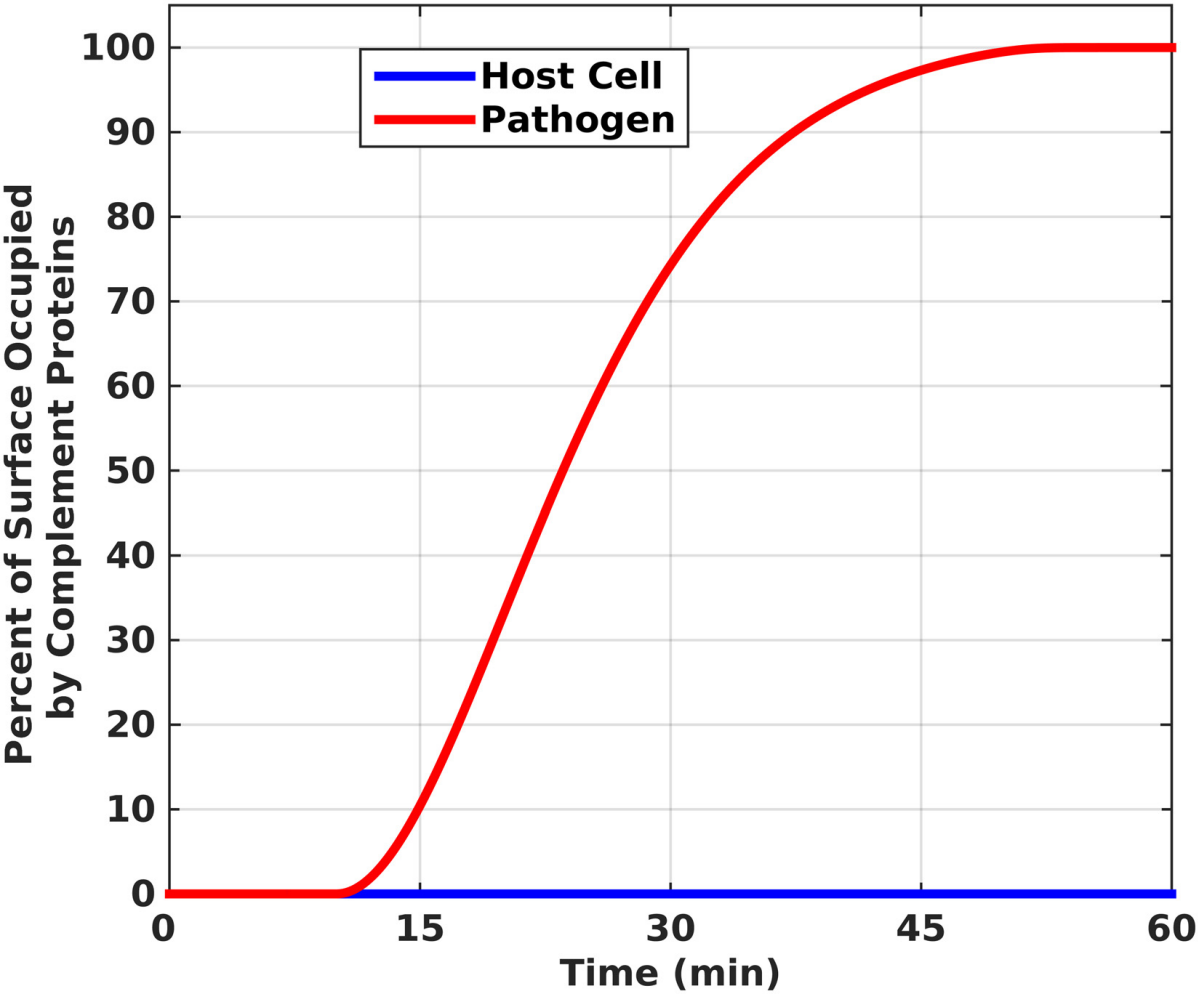
Complement deposition on pathogen and host surfaces. Saturation of pathogen surface is reached in 54 minutes with complement proteins such as C3b, iC3b, iC3bP (pathogen), C3dg, C3/C5 convertases, properdin (P*), MAC, and intermediates such as C3bB, C3bBP, C5b6-7, C5b6-8, and others. Propagation of the alternative pathway is inhibited on host cells resulting in complement proteins occupying significantly less than 1 percent.

**Fig 3:**
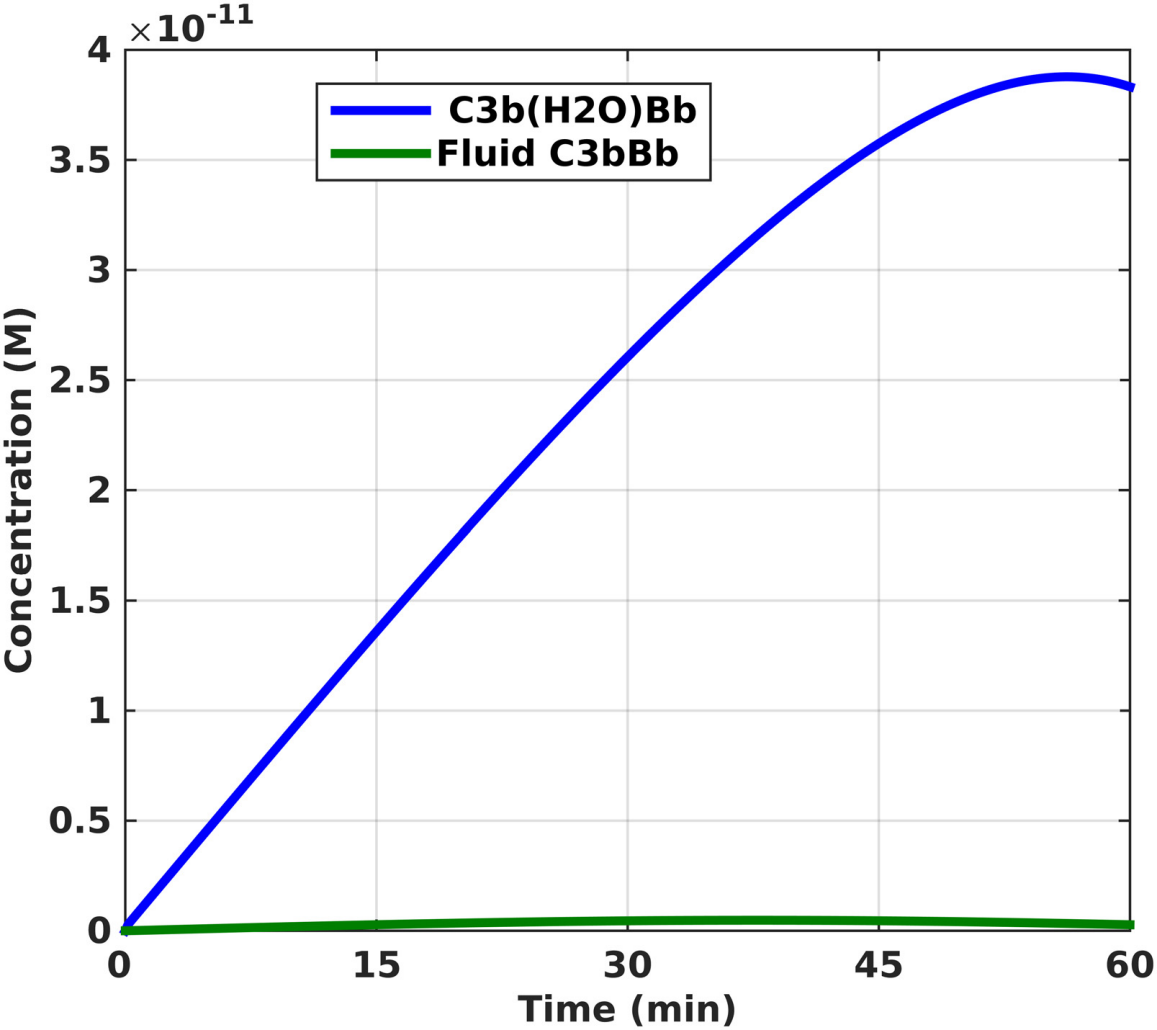
Concentration of assembled fluid phase convertases, C3(H_2_O)Bb and C3bBb, of the alternative pathway. The time profiles show that the concentration of C3(H_2_O)Bb increases significantly within 60 minutes compared to the concentration of C3bBb.

Our model incorporates a set of rate equations that describes the biochemical reactions shown in Fig 1. The alternative pathway is activated by the tick-over reaction of C3 and leads to a C3b molecule that indiscriminately binds to host cells/pathogens and initiates a set of cascade reactions. Fig 2 shows the percentage of host and pathogenic cell surfaces occupied by complement components, including C3/C5 convertases, C3b, iC3b, iC3bP, C3dg, P*, MAC, and all intermediate species like C3bB, C3bBP, C5b6-7, C5b6-8, and others. A sigmoidal response is generated on the surface of pathogens that plateaus in 54 minutes. Preceding the robust activation, there was a lag phase of 11 minutes. Complement components occupy half of the available space on the pathogen surface in 24 minutes during the exponential phase of the sigmoidal response. However, due to the presence of complement regulators on host cells, significantly less than 1 percent of their surface is covered by complement components, as shown in Fig 2. Extended time profiles to 180 minutes are shown in S1 Fig. One of the main catalysts for the amplification loop in the alternative pathway is the production of C3 convertases that rapidly cleave C3 into C3a and C3b. Fig 3 shows the response generated for the initial (fluid phase) convertase, C3(H_2_O)Bb, and fluid phase convertase, fC3bBb. In 35 minutes, fC3bBb reaches a peak concentration of 4.6×10^−13^ M and starts declining to reach a concentration of 2.7×10^−13^ M in 60 minutes while C3(H_2_O)Bb reaches a concentration of 2.9×10^−11^ M in 35 minutes and continues to reach a concentration of 3.8×10^−11^ M in 60 minutes. Thus, C3(H_2_O)Bb seems to be the primary fluid phase convertase responsible for C3 cleavage, with significantly higher concentration than fC3bBb, despite possibly minor differences in their enzymatic activity against C3. Extended time frames to 180 minutes (S2 Fig), show that the concentration profile of both C3(H_2_O)Bb and fC3bBb flattens in 105 and 75 minutes, respectively, owed to the dissociation of Bb subunit and the regulatory action of Factor H that degrades formed convertases.

**Fig 4:**
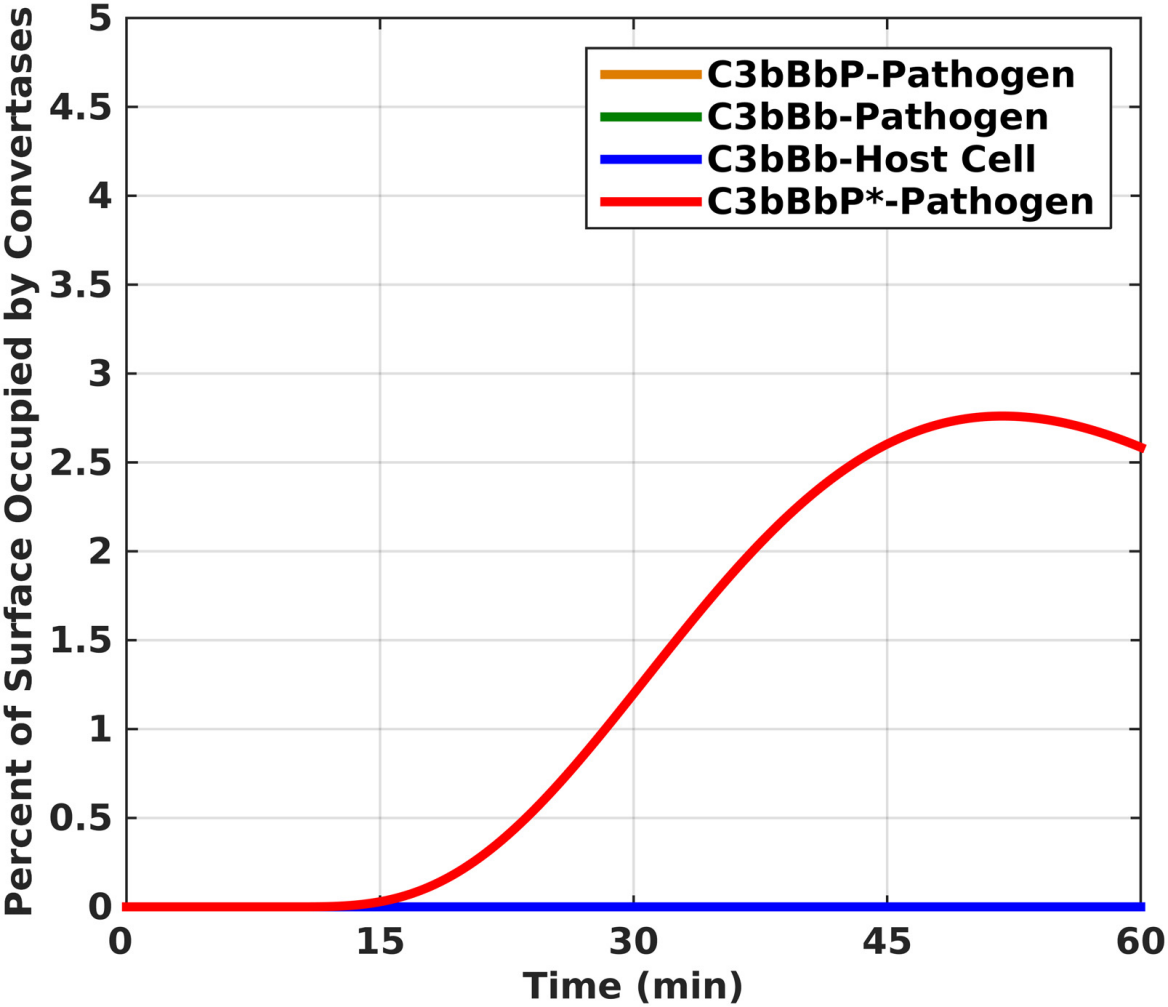
Formation of C3 cleaving enzymes on the surface of host cells and pathogens. C3bBbP* occupies 2.7 percent of the pathogen surface in 51 minutes. The asterisk denotes properdin released from neutrophils. The time profile of C3bBbP* shows a curve with a lag phase followed by an accelerated production (surface occupational) phase. The production of C3bBb and C3bBbP occupies significantly less than 1 percent on pathogens and even less on host cells. The orange and green lines are obscured by the blue line in the figure.

The cascade of reactions initiated by C3b also form convertases on the surface of pathogens and host cells as shown in Fig 4. Despite the presences of three types of convertases on the surface of pathogens (C3bBb, C3bBbP, and C3bBbP*), the properdin-initiated activation of the alternative pathway convertase, C3bBbP*, is the dominant C3 cleaving enzyme present. The asterisk means that properdin is secreted from neutrophils and binds to the surface first to act as an initiator of *de novo* formation of convertase, whereas lack of asterisk means that C3b binds first to the surface and initiates convertase formation. In 51 minutes, C3bBbP* occupies 2.7 percent of the pathogen surface while C3bBb and C3bBbP occupy significantly less than 1 percent. On the surface of host cells, C3bBb also occupies significantly less than 1 percent. The characteristic response of C3bBbP* production shows a lag phase of approximately 11 minutes followed by an accelerated production (surface occupational) phase. This phase starts slowing down after 53 minutes to eventually reach the decelerated occupational phase of the convertase. A similar characteristic response was also observed for pathogen-bound C3bBb but at significantly less than 1 percent occupation, if one zooms in Fig 4 (zoom-in data not shown). Even in extended time frames of 180 minutes, all three convertases, (C3bBb-Pathogen, C3bBbP-Pathogen, and C3bBb-Host Cell), occupy significantly less than 1 percent (S3 Fig). However the extended half-life of C3bBbP* highlights the intricate role P* plays for the amplification loop of the alternative pathway.

**Fig 5:**
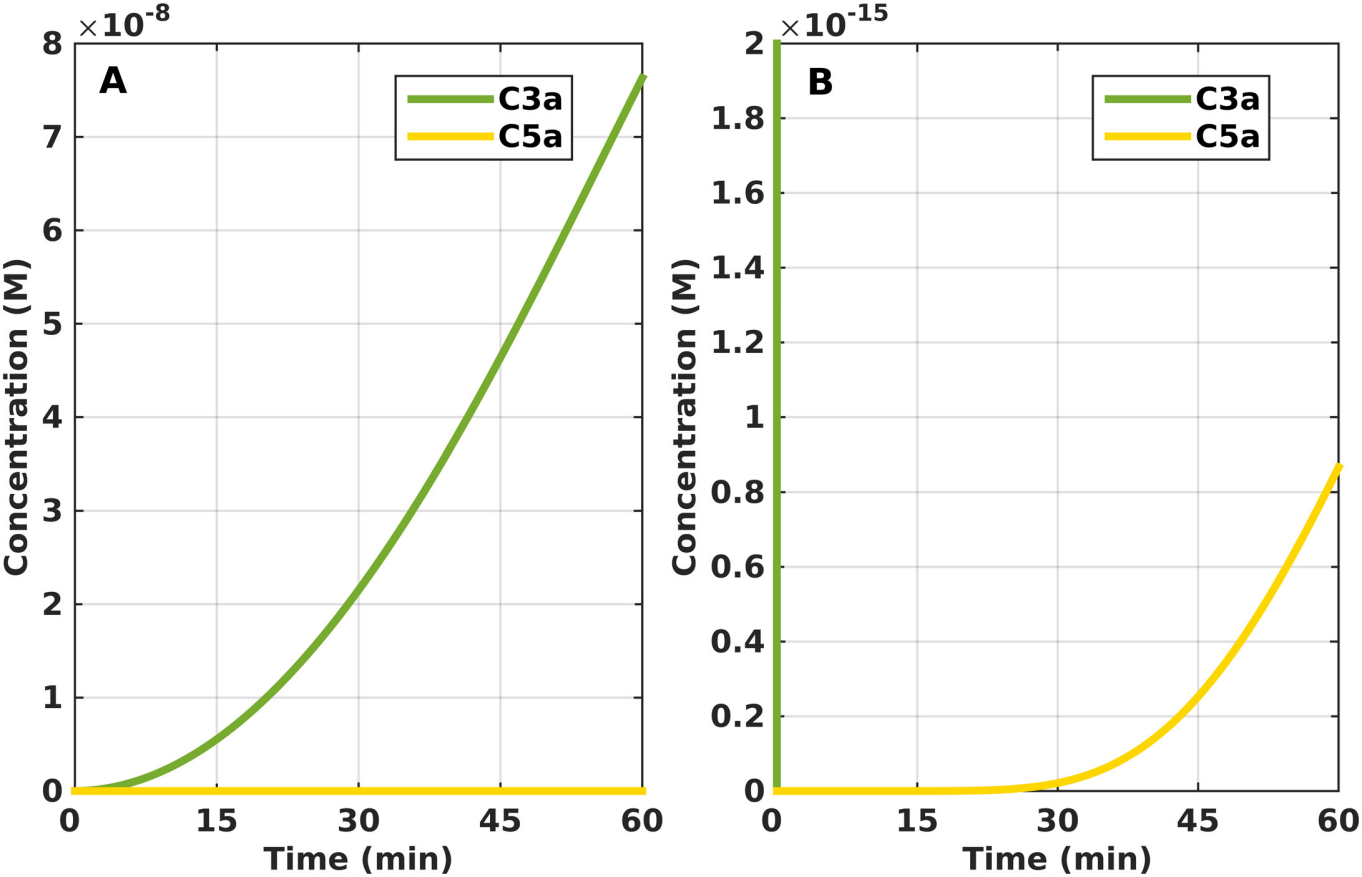
Time profile for the production of C3a and C5a. (A) The response generated for C3a shows a lag phase that is followed by an accelerated production phase. In 60 minutes, the amount of C3a produced is 10^8^ times greater than that of C5a. (B) Zoom-in of panel (A) to show the time profile of C5a.

As concentrations of the surface bound convertases decreases over time due to the Bb subunit dissociating and C3 convertase enzymes becoming C5 convertases (C3bBbC3b), the formation of C3bBbC3b initiates the cleavage process of C5a from C5. The total amount of anaphylatoxins C3a and C5a produced from C3 and C5 convertases are shown in Fig 5. Within 60 minutes, C3a reaches a concentration of 7.6×10^−8^ M while C5a reaches 8.4×10^−16^ M (Fig 5). We can see from the response generated that the amount of C3a produced in 1 hour is 10^8^ times greater than C5a. However, C3a does plateau in 105 minutes with a concentration of 11.5×10^−8^ M while that of C5a reaches a concentration of 2.4×10^−16^ M within the same time frame (S4 Fig).

**Fig 6:**
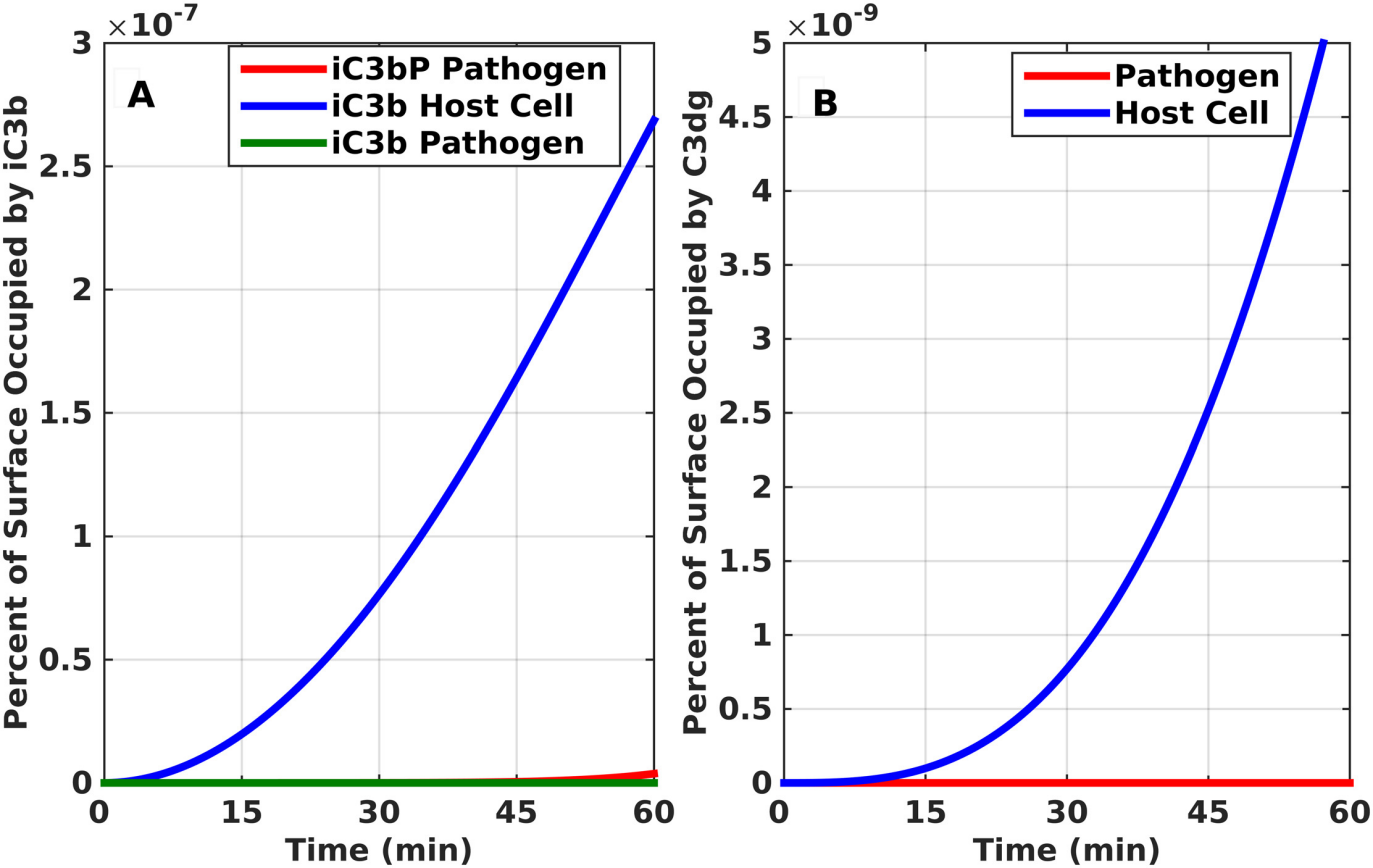
Time profile production for cleavage products of C3b. iC3b and C3dg. (A) iC3b. (B) C3dg. Both proteins take significantly less than 1 percent of pathogen and host cell surfaces.

Surface-bound (host cell) and fluid phase regulators bind and act as cofactors for Factor I, which then cleaves C3b to its inactive derivative, iC3b. Factor I also further degrades iC3b into C3dg, through the intermediate C3c. The formation of iC3b and C3dg on pathogen surface is also possible due to the CR1 acting exogenously from the surface it is expressed. The final outputs of iC3b and C3dg are shown in Fig 6. Our data for surface-bound iC3b (Fig 6A) and C3dg (Fig 6B) shows that they account for significantly less than 1 percent of the pathogen and host cell surface.

**Fig 7:**
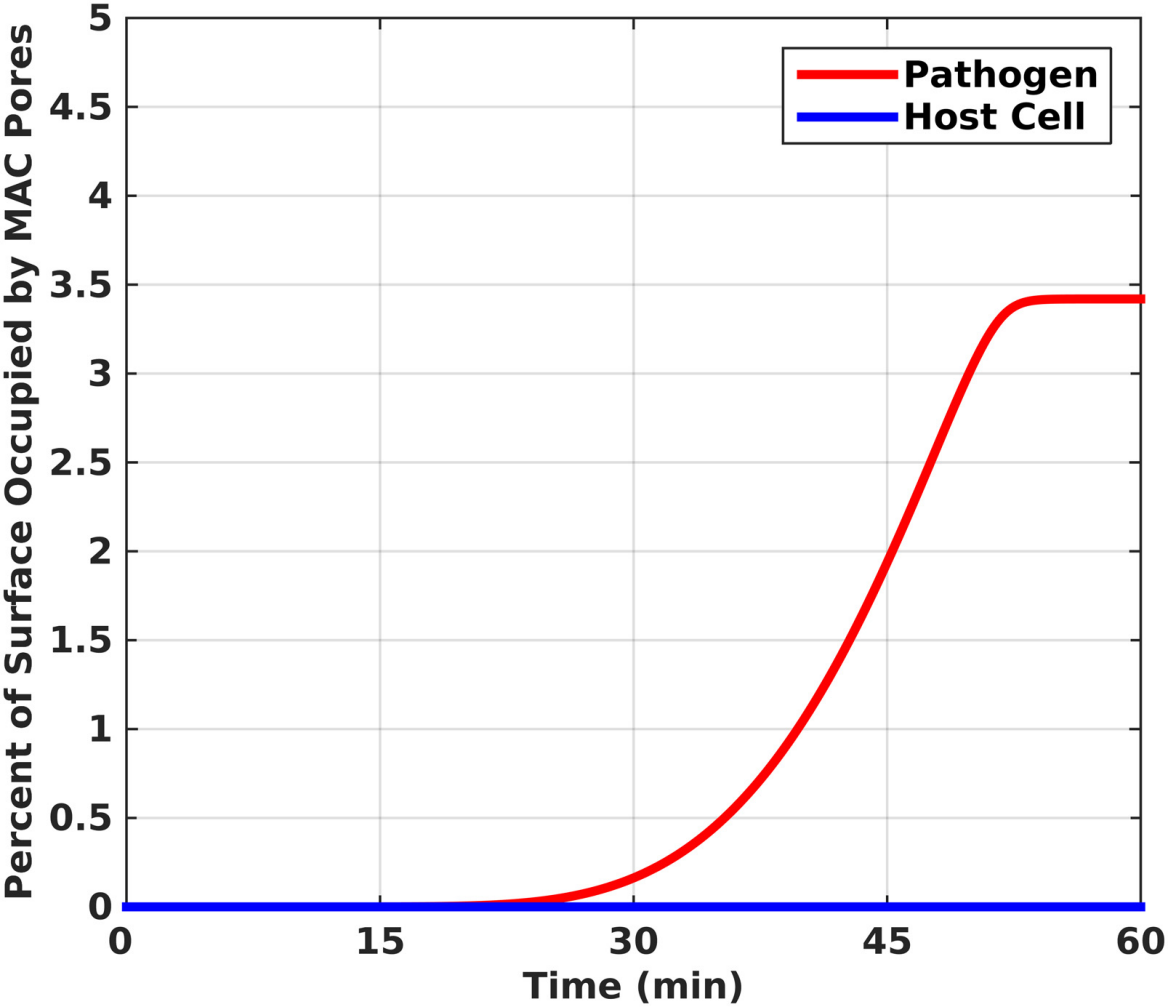
Formation of MAC pores is characterized by three phases: lag phase, production phase, and a steady state phase. The first 22 minutes present a lag phase that is followed by a rapid production of MAC pores, which plateaus in 54 minutes and occupies 3.3 percent of the pathogen surface. However, on host cells the MAC pores take significantly less than 1 percent.

After the formation of the C5 convertase, a cascade of reactions is initiated that forms MAC. This complex produces pores that render the pathogen/host cell amenable to lysis. In Fig 7, the amount of formed MAC pores occupies 3.3 percent of total pathogen surface, while taking significantly less than 1 percent on the surface of a host cell. The MAC pore production on pathogens is characterized by three phases: lag phase, production phase, and a steady state phase. The lag phase is 22 minutes long and is followed by a pronounced production of MAC pores that plateaus in 54 minutes. It is worth noting the time frame for MAC pore production to plateau is the same as the time frame for covering the entire pathogen surface (Fig 7) by complement components (Fig 2). Furthermore, this process highlights the rapidity of complement cascade that leaves no more than 3.3 percent of the pathogen surface for MAC pores to take root, also shown at extended time frames of 180 minutes in S5 Fig.

**Fig 8:**
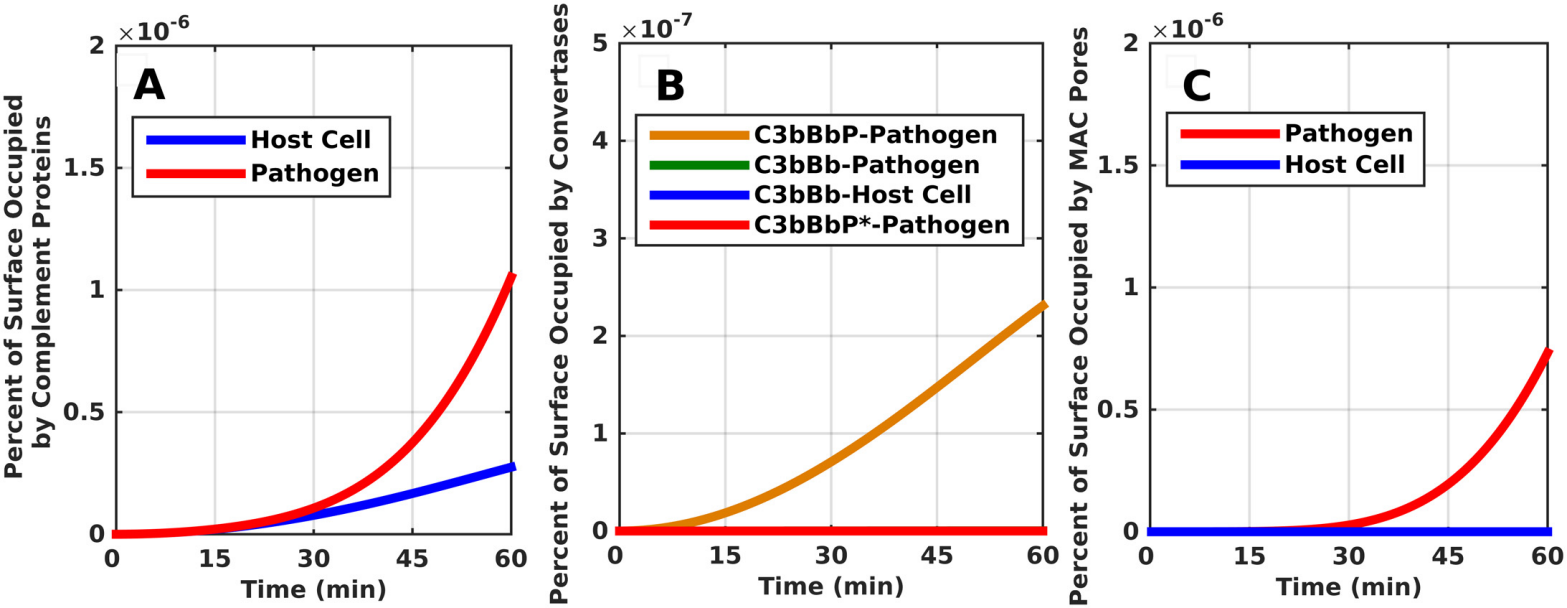
Time course for properdin (P*) as a stabilizer and not an initiator of the alternative pathway. (A) More than 99 percent of pathogen surface remains unoccupied by complement components. (B, C) The C3 convertases and MAC pores occupy significantly less than 1 percent on pathogens and even less on host cells.

The only positive regulatory protein of the complement system, properdin, has emerged more than a stabilizer of the C3 and C5 convertases. It has been shown as a molecule that distinguishably binds to specific cell surfaces and initiates complement activation [14–22]. To gain a deeper understanding on the role of neutrophil-secreted properdin (P*) during complement activation, we inhibited the ability of properdin (P*) to bind to surfaces and recruit C3b, but the stabilizing role of properdin (P/P*) on convertases was not inhibited. The results are shown in shown in Fig 8. Compared to Fig 2 where the pathogen was fully saturated by complement components in 54 minutes, we see in Fig 8A that complement components account for significantly less than 1 percent of the pathogen surface. The key enzyme responsible for the robust activation of the alternative pathway is the assembly of the C3 convertase. We can see from Fig 8B that the convertase in its various forms also accounts for significantly less than 1 percent of the pathogen surface. In contrast, in Fig 4, the convertase assembled from properdin (P*) accounted for 2.7 percent of pathogen surface. Besides affecting the amplification loop of the alternative pathway, properdin (P*) also affects the end product of the terminal cascade, MAC pores. Compared to Fig 7, where MAC pores accounted for 3.3 percent of pathogen surface, Fig 8C shows that MAC pores take significantly less than 1 percent of the pathogen surface. Extension of the time frame beyond 60 min leaves the plotted surface areas in Fig 8A–8C at significantly less than 1 percent (data not shown).

### Regulation (host cell and fluid phase)

**Fig 9:**
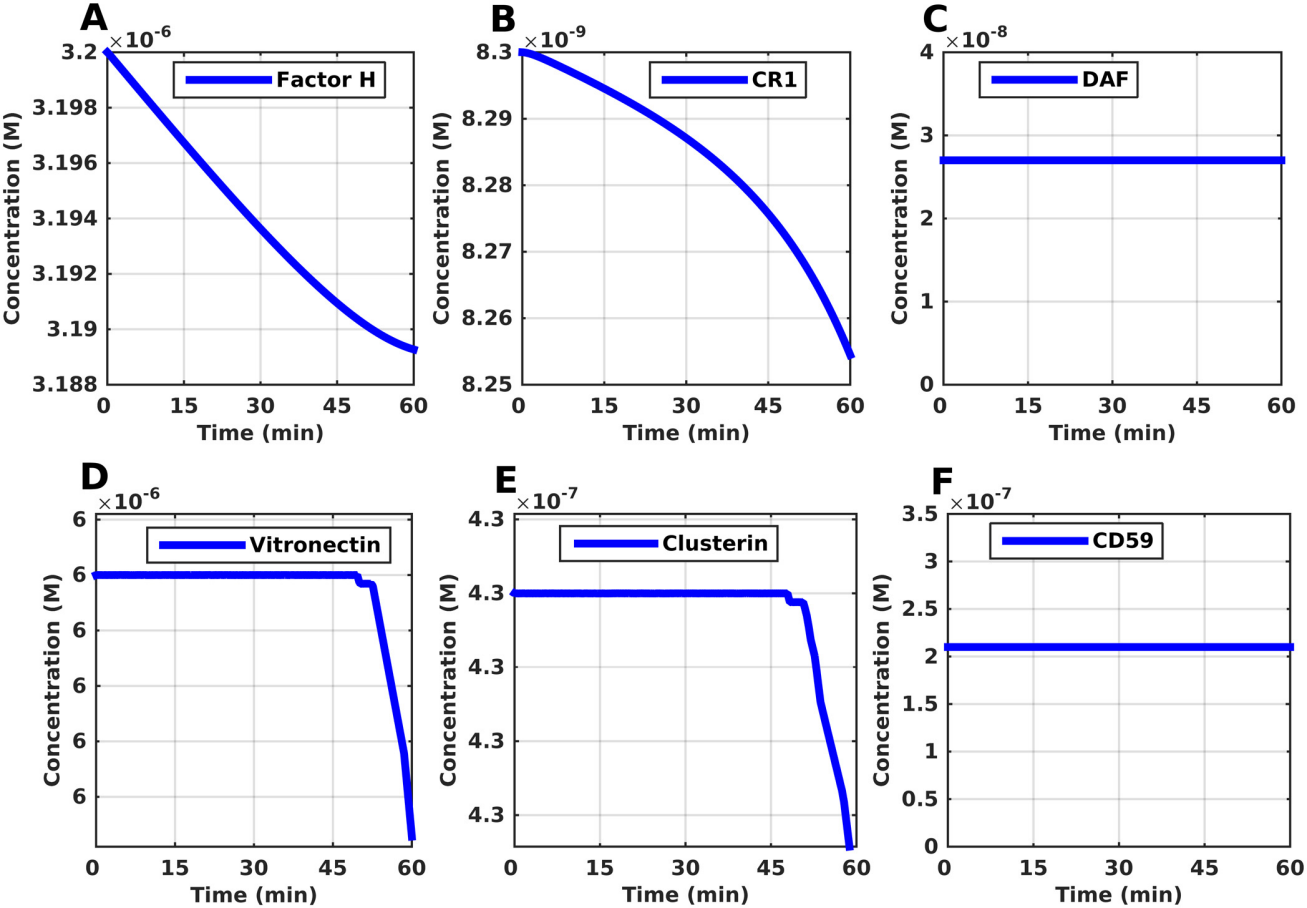
Time profiles for complement regulators Factor H, CR1, DAF, vitronectin, clusterin, and CD59. (A, B) The concentrations of Factor H and CR1 decrease, with Factor H showing initially a linear like response. The initial response of Factor H signifies the regulation taking root at the start of the AP by inhibiting C3(H_2_O) and C3(H_2_O)Bb. (C) The concentration of DAF remains constant and does not change, which highlights the rapid deactivation of any C3b by FH and CR1. (D,E) Vitronectin and clusterin experience a long delay of 52 and 51 minutes respectively before their concentration starts decreasing. Their regulatory component takes root at the formation of fluid C5b-7, which comes at a later stage of complement activation. (F) The response generated for CD59 is the same as that of DAF (no change in concentration). This is expected since DAF remains the same (inhibition of C3 convertase formation), the terminal cascade for MAC pore formation will not take root because formation C5 convertases will also be inhibited.

Due to the destructive effects of the complement system on pathogens, the body has implemented several mechanisms to prevent its uncontrolled activation on host cells by using complement control proteins that are present in plasma and bound on host cell membranes. Fig 9 presents the results generated for fluid phase (Factor H, vitronectin, clusterin) and surface-bound (CR1, DAF, CD59) complement regulators. The time profile of the concentration of Factor H rapidly decreases (Fig 9A), while that of CR1 also decreases, but the decrease is not as rapid as Factor H. (Fig 9B). This is expected because despite the fact that both Factor H and CR1 regulate the initial complement protein of the alternative pathway, C3(H_2_O), Factor H has a higher binding affinity. In addition to regulating the hydrolyzed C3 (C3H_2_O), Factor H can also bind to C3bBb and C3(H_2_O)Bb to promote their rapid decay. Regulatory proteins on host cell membranes, such as CR1 and DAF also inhibit the formation and/or promote rapid dissociation of convertases [23–25]. While the concentration of CR1 decreases (Fig 9B), that of DAF does not change (Fig 9C). This highlights how rapidly CR1 and Factor H inactivate C3b to inhibit the formation of the C3 convertases on host cells and also their continued complement regulation in the fluid state. The time profiles of Factor H, CR1, and DAF at extended timeframes are shown in S6A–S6C Fig. If the formation of the C3 convertase is tightly controlled, one can conclude that the terminal cascade will be effectively shut down because the formation of the C5 convertase will also be inhibited.

MAC production is inhibited on host cells by a combination of complement regulatory proteins that reside on host cells and in fluid phase. Vitronectin and clusterin are fluid phase regulators that inhibit MAC formation by binding to C5b-7, while CD59 inhibits C9 polymerization to prevent pore formation on the lipid bilayer of host cells [23–27]. Fig 9D–9F shows the time profiles generated for vitronectin, clusterin, and CD59, respectively. Both vitronectin and clusterin experience a long delay of about 52 and 51 minutes, respectively, before their concentration is reduced (Fig 9D and 9E). The lag response is expected because vitronectin/clusterin inhibit C5b-7 from attaching to host cells and the production of C5b-7 comes at a later stage of complement activation. However, the concentrations of vitronectin and clusterin do reach a steady state in 105 minutes and 124 minutes, respectively (S6D and S6E Fig). Lastly, the time profile generated for CD59 (Fig 9F and S6F Fig) shows that its concentration does not change. This type of response is also seen for DAF. Since CR1 and Factor H inhibit the formation of C3/C5 convertases, the propagation of the terminal cascade is tightly controlled, thus effectively leaving no MAC pores to be regulated by CD59, under physiological conditions.

### Sensitivity analysis

**Fig 10:**
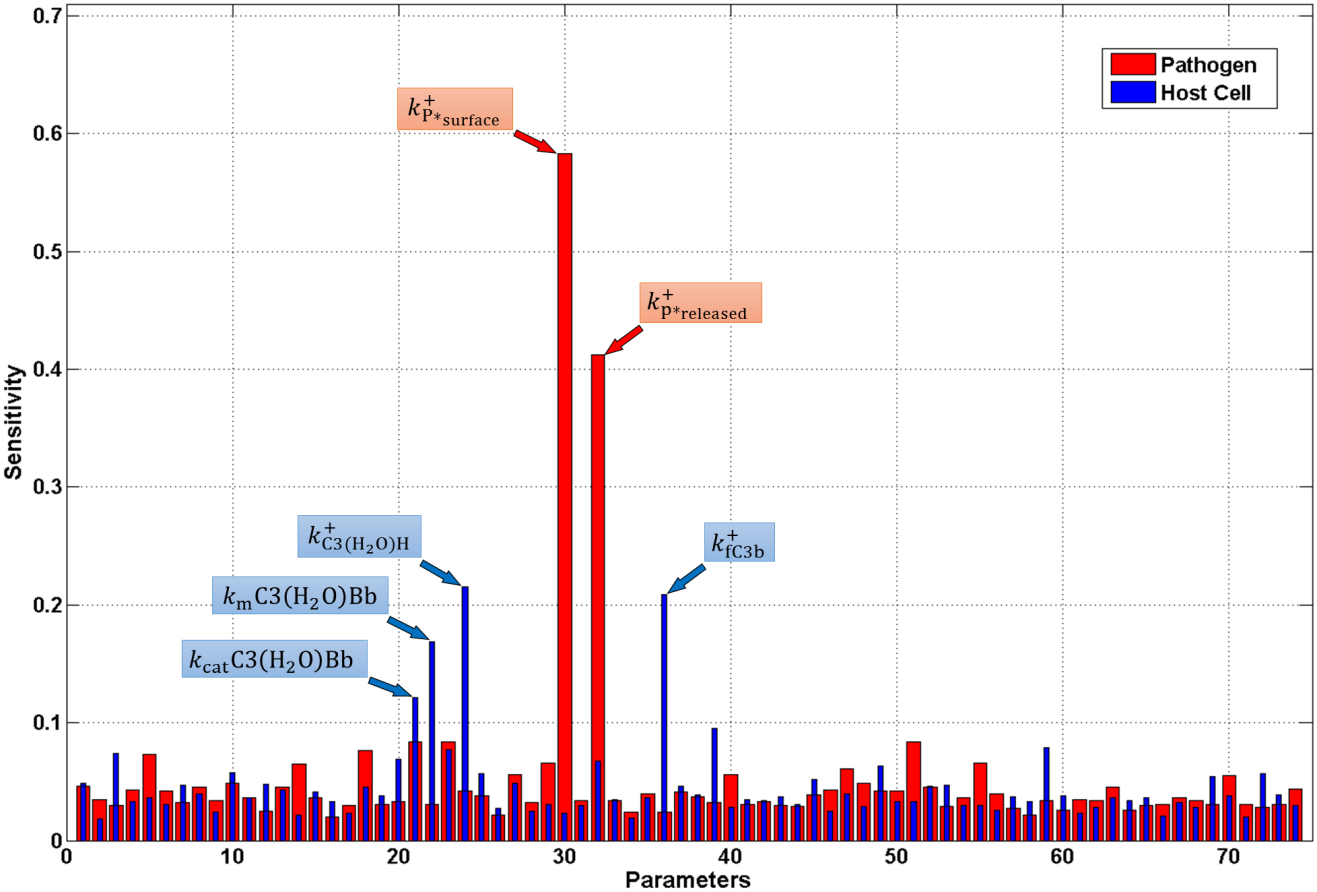
Results of multi-parametric sensitivity analysis to kinetic parameter values. The most sensitive rates on the surface of pathogens (red) are $k_{{\text{P}*}_{\text{surface}}}^{+}$, and $k_{{\text{p}*}_{\text{released}}}^{+}$, which represent the rate at which properdin associates to the surface and the rate at which it is released from neutrophils, respectively. While most kinetic parameter are mildly sensitive on host cells (blue), $k_{\text{C}3\left({\text{H}}_{2}\text{O}\right)}^{+}$ and $k_{\text{fC}3\text{b}}^{+}$, which represent association of Factor H to hydrolyzed C3 and conversion rate of nfC3b to fC3b, respectively, are more sensitive to parameter variations. The Michaelis–Menten kinetic parameters, *k*_cat_ and *K*_m_ of the fluid phase convertase C3(H_2_O)Bb are also sensitive.

Multi-parametric sensitivity analysis (MPSA) was used to identify initial complement concentrations and kinetic parameters that are critical for the four dynamic steps of the alternative pathway. Table 1 shows the results of the relative sensitivity of the initial concentrations on the surface of pathogens and host cells. C3, the central protein of the alternative pathway, strongly influences the response of the system, in contrast to initial concentrations of all other complement components that have low sensitivities. This is likely due to the fact that C3 cleavage is required for both activation and amplification of the alternative pathway, and thus most rates and kinetic parameters are dependent on the amount of C3 in the system. Next, sensitivity analysis was performed in order to identify the critical kinetic parameters for the output of the model. The results are shown in Fig 10, and indicate that while responses on both host cells and pathogens are mildly influenced by most of the kinetic parameters, the association rate constant of properdin (P*) to the surface of pathogens and the rate at which neutrophils release properdin (P*), are greatly important for the activation and amplification of the alternative pathway on pathogen surfaces. In addition, complement response on host cells was more sensitive to parameters involving fluid-phase reactions compared to surface-bound reactions. These rates include association rate constant of Factor H to fluid C3(H_2_O), formation of fC3b from nfC3b (half-life of the thioester bond), and the Michaelis-Menten constants that govern the cleavage rate of C3 by the fluid phase convertase, C3(H_2_O)Bb. Thus, the response of the system is sensitive to the parameters $k_{{\text{P}*}_{\text{surface}}}^{+}$, $k_{{\text{p}*}_{\text{released}}}^{+}$,$k_{\text{C}3\left({\text{H}}_{2}\text{O}\right)}^{+}$, $k_{\text{fC}3\text{b}}^{+}$, and *k*_cat_, and *K*_m_ of C3(H_2_O)Bb.

**Table 1:** Results of multi-parametric sensitivity analysis to initial complement concentrations. In both pathogens and host cells, C3 strongly influenced the model output. Large values of K-S (Kolmogorov-Smirnov) test indicate that the model output is sensitive to the given parameter variations.

Complement proteins	K-S: pathogen	K-S: host
C3	1.0	1.0
C5	0.024	0.026
C6	0.046	0.039
C7	0.025	0.020
C8	0.036	0.031
C9	0.031	0.0
Factor B	0.031	0.031
Factor D	0.036	0.036
Factor I	0.020	0.022
Properdin (P)	0.023	0.0
Properdin* (P*, neutrophil-secreted)	0.041	0.037
Factor H	0.035	0.024
CR1	0.037	0.037
DAF	0.058	0.0
Vitronectin	0.057	0.029
Clusterin	0.022	0.032
CD59	0.0	0.0

## Discussion

We have developed a mathematical model that describes the dynamics of the alternative pathway of the complement system, initiating in fluid phase with activation of C3, surface-bound amplification of C3b and C3b-contaning convertase formation, and terminating at surface-bound MAC (Fig 1). We modeled two surfaces, a host cell and a bacterial cell surface, using an erythrocyte and an *E coli* cell as examples for quantification. Cells were used as a basis for extracting dimensions, without accounting for cell surface markers and chemical compositions. We have also incorporated in the model complement regulation in fluid phase and on host cells. The mathematical model consists of a system of 107 ordinary differential equations, describing the time course of the concentrations of 107 proteins, protein fragments, or protein complexes, and we have generated graphs of their concentration time profiles. We have asked questions of the type: (i) What are the quantitative contributions of different modules of the alternative pathway propagation, such as fluid phase activation, the amplification loop, and terminal cascade beyond C3 and C5, in generating opsonins, inflammatory proteins C3a/C5a, and MAC? (ii) What is the difference in complement deposition on pathogen and host cells? (iii) What are the subtleties of negative and positive complement regulation in inhibiting or promoting complement deposition on host and pathogen cells, respectively?

We present key differences in complement function on pathogen and host surfaces, demonstrating the significance of negative complement regulation on host surfaces. We also present the tremendous influence of properdin (P*) in positive complement regulation on pathogen surfaces, and discriminate the contribution of different types of properdin (P, P*) molecules in eventually promoting MAC formation.

We have shown the efficiency of the alternative pathway in saturating the surface of pathogen with complement proteins, such as C3/C5 convertases, C3b, iC3b, iC3bP (pathogen), C3dg, properdin P*, and MAC, while regulation on host cells limits complement component deposition to less than 1 percent (Fig 2). The response generated on host cells is expected due to the function of surface-bound or circulating regulatory proteins to ensure inhibition on host cells, while not hindering activation on pathogens. Convertases play a critical role in the propagation and amplification of the alternative pathway. The initial alternative pathway convertase, C3(H_2_O)Bb, is a fluid phase convertase that cleaves C3 into C3a and C3b [28]. This nascent C3b, nC3b, is a very reactive complement protein due to its exposed internal thioester bond that is capable of indiscriminately binding to pathogens and host cells via covalent bond [29–31]. However, the short half-life of the thioester (60 μsec) ensures most of the nC3b produced will interact with water and form the fluid phase C3b, fC3b, that never attaches to a surface [32]. This fC3b can also form a convertase that is capable of cleaving C3 in the fluid phase. Due to the dangers these fluid phase convertases present if left unregulated, complement regulatory proteins promote their rapid decay, both in fluid phase and on host cell surfaces. In Fig 3, we see the concentration of fC3bBb is smaller than that of C3(H_2_O)Bb. This is because only one complement protein, Factor H, regulates C3(H_2_O)Bb, while fC3bBb is regulated by Factor H and CR1 [28,33–39]. However, the possibility for C3(H_2_O)Bb regulation by CR1 cannot be excluded because it has been shown that CR1 can bind to C3(H_2_O) [40]. In addition, we must exercise caution in interpreting the results for C3(H_2_O)Bb activity, as sensitivity analysis showed that complement activation on host cells is influenced by the Michaelis-Menten kinetic parameters of this enzyme. In any case, CR1 and Factor H regulate AP by (i) competitively binding to C3b, (ii) inhibiting Factor B binding, and (iii) by accelerating the decay rate of convertases.

If the nascent C3b reacts and forms a covalent bond on either the pathogen or host surface, it will associate with Factor B, which becomes cleaved by Factor D to yield the membrane-bound C3 convertase, C3bBb [41–44]. Fig 4 shows difference in the amount of area these convertases occupy on the host cells and pathogens. The destructive effects of complement activation on host cells are tightly regulated through surface-bound regulators such as CR1, DAF, and plasma proteins such as, Factor H in conjunction with Factor I [33,36,37,39,45–47]. However, these regulators leave complement activation unchecked on the surface of pathogens. Furthermore, it is also evident from Fig 4 that C3bBbP* is the more dominant convertase than C3bBb and C3bBbP. (Note that the asterisk denotes properdin released from neutrophils which binds to the surface first and acts as an initiator of *de novo* formation of convertase, whereas lack of asterisk means that C3b binds first to the surface and initiates convertase formation, subsequently stabilized by properdin.) While C3bBb is relatively unstable with a half-life of about 90 seconds, the positive regulatory protein, properdin, associates with C3bBb [41,48], which stabilizes and increases the half-life of the convertase by about 10 fold. However, it has been shown that properdin bound to a surface can initiate *in situ* complement activation by forming C3bBbP* [14–22]. And unlike the standard AP activation through nC3b attaching to a surface and initiate complement, properdin (P*) can attach to a surface and recruit nC3b or fC3b to form C3 convertase.

The cleavage of C3 and C5 by their respective convertases also produces complement fragments C3a and C5a. They belong to a family of anaphylatoxins that are released into the fluid phase during complement activation [49,50] (Fig 5). But there are differences in amount released, with C3a being more abundant than C5a (Fig 5A). This stems from having more C3 convertases on the surface of the pathogen/host cell than C5 convertases that are responsible for generating C5a. In addition, the large number of C3 cleaving enzymes can be attributed to properdin (P/P*) stabilizing preformed C3bBb, and also by neutrophil-secreted properdin (P*) that can accelerate the association of C3b and Factor B to subsequently form a stabilized convertase, C3bBbP*. However, C5 can also be cleaved by the monomeric C3 convertase (C3bBb) but has been shown to have a weak affinity for C5 (K_m_ = 24 μM) [51]. In the absence of control factors, the monomeric C3 convertase will cleave 9000 C3 molecules for every C5 molecule cleaved [52,53]. While both fragments play an important role in mediating inflammatory response, C5a is a more potent mediator than C3a. Furthermore, C5a is also a potent chemoattractant for neutrophils, T cells, and tonsillar B cells [54–56]. It is likely that because of the strong C5a effect in inducing a more pronounced inflammatory response compared to C3a, less of C5a is released during complement activation to achieve the desired response (Fig 5B).

In Fig 6A, we present the response of the inactive derivative of C3b known as iC3b on the surface of pathogens and host cells. Surface-bound regulator CR1 binds to C3b and acts as a cofactor for Factor I cleavage of C3b into iC3b [37–39]. In addition, CR1 is the main cofactor for Factor I mediated enzymatic breakdown of membrane-bound iC3b into C3dg (Fig 6B). However, CR1 is unique in its ability to also function extrinsically (on surrounding cells), from the cell in which it is expressed [25,57,58]. This explains that even though pathogens lack membrane-bound complement inhibitor CR1, the inactive derivative iC3b and subsequently C3dg are present on pathogen surface. Although iC3b and C3dg are inactive in forming convertases and contributing to the propagation of complement activation, their deposition on pathogen cell surfaces is critical for efficient phagocytic clearance and enhancement of adaptive immune response.

Continued activation of the alternative pathway leads to the terminal cascade that is responsible for the production of MAC. This terminal multi-component C5b-9_18_ complex forms pores on the surface of a pathogen/host cell, which causes lysis due to disruption of the osmotic balance inside/outside the cell. The MAC pores occupy significantly less than 1 percent on the surface of host cell because of regulators that are present in fluid phase and also bound to the surface (Fig 7). For instance, vitronectin and clusterin are fluid phase regulators that bind to C5b-7 and inhibit its insertion into the lipid bilayer [59,60]. Surface-bound regulators such as CD59 inhibit polymerization of C9, while DAF and CR1 accelerate the decay of C5 convertase by altering the interaction between C3bBb [27,45–47,61]. However, the absence of these regulatory proteins on pathogens leads to unchecked terminal cascade activation that results in 3.3 percent occupation of the pathogen surface by MAC pores (Fig 7).

An important aspect of our study is the re-emergence of the role of properdin in complement function. Properdin is more than a stabilizer of the C3/C5 convertase by its role in initiating *in situ* complement activation on different surfaces. The role of properdin as an instigator of the alternative pathway was first described by Louis Pillemer and his associates over 50 years ago [62]. This idea of properdin-directed activation was challenged by Robert Nelson, who suggested the role of antibodies in explaining the observed complement activation [63]. By eliminating the surface binding ability of neutrophil-secreted properdin (which also inhibits C3b recruitment) and keeping its convertase stabilizing role, we observe that complement activation products occupy significantly less than 1 percent of pathogen surfaces, as shown in Fig 8A–8C. Our results show that eliminating properdin’s (P*) surface binding and C3b recruitment abilities effectively reduced propagation of the alternative pathway. In addition, results from the sensitivity analysis showed that the response of the system is sensitive to the surface binding ability of properdin (Fig 10). Binding to surfaces is soon followed by the recruitment of C3b, to form convertases. This sets in motion the amplification loop, which is very important for the propagation of alternative pathway. The key substrate that makes this step possible is C3, and is also indicated by the results of the sensitivity analysis as the most important complement component (Table 1). Thus, the widely-accepted role of properdin as just a stabilizer of convertases is not sufficient to support robust propagation of the alternative pathway. To highlight this point, a study by Gupta-Bansal et al [64] showed that anti-properdin MoAbs that blocked binding of properdin to C3b caused a dose-dependent inhibition of AP in vitro.

It is interesting to note that studies performed in serum-derived properdin, in which long term storage, freezing, and thawing were involved, formed highly activated properdin aggregates that not only stabilized convertases but also activated complement in solution [21,65]. However, a recent finding showed serum-derived properdin (without aggregates) was able to attach to activated platelets and recruit both C3b and C3(H_2_O) to generate C3 convertases (C3bBb and C3(H_2_O)Bb), thus initiating complement activation on the surface [20]. In addition, properdin bound to activated platelets was 3- and 2-fold more capable of recruiting C3b and C3(H_2_O), respectively, compared with activated platelets alone. The same study also showed platelets that first received C3(H_2_O) or C3b, only formed convertases after properdin was recruited. Although these studies used serum properdin, our results are consistent with their data because robust propagation of AP (by forming convertases) was achieved only after properdin that had the ability to bind to surfaces and recruit C3b was added. This event was considered in our system for neutrophil-derived properdin (P*), while serum-properdin (P) only stabilized convertases. Our multi-parametric sensitivity analysis indicates that kinetic parameters associated with C3(H_2_O) and C3b are sensitive to variations (Fig 10), thus making the fluid phase a pivotal battlefront for protecting/shielding host cells.

Gupta-Bansal et al [64] also used the anti-properdin antibody to inhibit activation of AP and reduce neutrophil and platelet activation in their cardiopulmonary bypass model. We believe the reduction of neutrophil activation is an important step because after neutrophils are stimulated they have the ability to not only release properdin (P*) but also release C3, Factor B, C6, and C7 [22,66–68]. In the presence of Factor D, properdin (P*) and the released C3, Factor B, C6, and C7 constitute all the components needed for the activation of the alternative pathway and terminal cascade with the generation of C3 and C5 convertases. This was shown on activated platelets after the release of properdin (P*) from neutrophils [20]. Conceivably, this observation may explain why Gupta-Bansal et al [64] saw reduced neutrophil activation and near complete inhibition of the C3a/C5b-9 formation with the anti-properdin antibody.

It is worth noting that in a standard AP hemolytic assay, T cell-derived properdin was shown to be 100 times more functionally active than serum-derived properdin [69]. Just as nC3b is an active form of C3 that must be constantly regulated to prevent unnecessary complement activation, the more active properdin (P/P*) must also be regulated. In light of the aforementioned recent findings, we speculate that the body constantly regulates properdin (P*) by only releasing it at sites of infection (inflammatory response) or by protease cleavage of functional sites that are involved in surface binding/C3b recruitment. Even serum properdin (P), was only able to bind to activated platelets (present during inflammation) and not resting platelets. However, to the best of our knowledge, there are no studies that describe how the body regulates properdin (P/P*).

The notion of properdin as a positive regulator of AP by stabilizing convertases must be re-examined. We speculate, in conjunction with experimental data cited above, that for the occurrence of robust activation and propagation of the alternative pathway, properdin either derived from serum or neutrophils must satisfy three criteria: (i) ability to bind to surfaces, (ii) ability to recognize selective patterns (activated *versus* nonactivated platelets), (iii) recruitment C3b/C3(H2O) and Factor B by properdin-bound surface to initiate complement activity. We believe all three criteria must be met and the sole role of stabilizing convertases is not enough to warrant properdin as a positive regulator of the alternative pathway. This is predicted by our model and is highlighted in the literature cited above. This notion also is consistent with what Pillemer and his colleagues observed over 50 years ago.

## Conclusions

We have presented a comprehensive mathematical model that describes the dynamics of the alternative pathway of the complement system, including activation, amplification, termination, and regulation. Our study quantitatively explains many known features of the complement system, but also reveals some less obvious or unexpected features. We have shown the profound differences of complement deposition on pathogen surfaces and MAC pore formation, compared to host surfaces. This result demonstrates the fine balance between activation and negative regulation during homeostasis, and the significant role of positive regulation in augmenting activation on pathogen surfaces. Complement deposition refers to C3b, iC3b, iC3bP (pathogen), C3dg, C3/C5 convertases, P*, MAC, and intermediates such as C3bB, C3bBP, C5b6-7, C5b6-8, and others. We have also shown many other features, described below. The initial C3 convertase of the alternative pathway, C3(H_2_O)Bb, has higher influence in the propagation of the alternative pathway, compared to the fluid phase C3 convertase, fC3bBb, suggesting the importance of the tick-over reaction in not only initiating, but also dominating the fluid state. Significantly higher concentrations of C3a are produced during the alternative pathway than C5a. The amounts of various forms of fluid phase or surface-bound C3b (nfC3b, nhC3b, npC3b, fC3b), and surface (pathogen or host) bound iC3b and C3dg are miniscule compared to those of fluid phase iC3b and fluid phase C3dg. Finally, neutrophil-secreted properdin (P*) re-emerges as an intriguing factor of complement activation, in addition to existing serum properdin (P) that is a C3/C5 convertase stabilizer. Properdin P* is a function enhancer by recruiting C3b ligand and FB to form a stabilized convertase, C3bBbP*, and also stabilize preformed C3/C5 convertases. The function of C3b as an opsonin is well-recognized, but our model highlights an alternative opsonin comprised of properdin (P*) and C3b (C3bP*), that may also serve as a pathogen tag. Our study suggests that the role of properdin (P*) in complement activation and positive regulation requires more experimental attention.

In conclusion, expanding on a characterization of C3 by a *Nature* Editorial [70], we consider the complement system as a sophisticated and well regulated “bloodstream patrol” that aims at attacking and possibly eliminating foreign bodies, while being controlled from harming self-tissues. A quantitative understanding of the intricacies and sophistication of the dynamics of complement activation, function, and regulation, as well as the mechanisms of inhibition by pathogens, will be an indispensable aid in the development of therapeutics targeting pathogenic infections, autoimmune and inflammatory diseases, and incompatibilities with biomaterial surfaces in the case of prosthetics and devices.

## Methods

### Numerical methods

Reactions in the alternative pathway of the complement system were formulated based on known interactions and kinetic parameters from the literature (Fig 1 and S1 Table). Based on these reactions, we generated a system of 107 ordinary differential equations and 74 kinetic parameters that describes the alternative pathway of the complement system (S1 Text). We have organized the biochemical reactions, equations, and subsequent discussion into four parts, which describe the following four modules of complement system activation and propagation: (i) initiation (fluid phase), Eqs 1–8 in S1 Text; (ii) amplification (pathogen), Eqs 1–18 in S1 Text; (iii) termination (pathogen), Eqs 1–19 in S1 Text; and (iv) regulation (host cell and fluid phase), Eqs 1–42 in S1 Text. In addition, we have grouped all the complement proteins present in fluid phase and bound on host cells into a fifth module, Eqs 1–20 in S1 Text. Law of mass action was used to assemble the equations. Enzymatic reactions were based on the Michaelis–Menten kinetics, and substrate competitions for enzymes such as C3 convertases was also taken into consideration. Known kinetic parameters were acquired from literature, while unknown parameters were attained via several different approaches. First, we implemented parameters estimated from Korotaevskiy et al [12], which used optimization procedure (combines solving of direct and inverse optimization problems) to estimate rate constants. Some parameters were implemented based on interactions of structurally or functionally homologous proteins, for which parameters are already known. For example, the rate constants of the interaction between C3b and Factor B are known, while those of C3(H_2_O) and Factor B are not. However, we assumed the same rate constants for the second case because C3(H_2_O) is known to be a C3b-like molecule that also has C3b-like functions. Remaining unknown parameters were estimated based on association and dissociation constants. For instance, since *K*_D_ *= k*^-^ */ k*^*+*^, if the *K*_D_ was known, we implemented a combination of *k*^-^ and *k*^+^ that would generate the given *K*_D_ based on physically relevant ranges of parameters (*k*^+^: 10^3^ to 10^9^ M^-1^s^-1^, *k*^-^: 10^−1^ to 10^−5^ s^-1^) [71–73]. These parameter combinations were tested and determined their effects on the dynamics of the system to be negligible in all cases (Fig 10). The remaining (9) parameters were completely unknown, and values were estimated based on functional data for other complexes and constrained according to physiological ranges of association and dissociation rate constants. Two of these parameters have a profound effect on the response of the system, and are discussed in the Results and Discussion sections. Initial concentrations of soluble complement components were obtained from literature, based on known homeostatic concentrations of these factors in human plasma (S2 Table). Furthermore, properdin released from neutrophils has been shown to be more active by initiating the alternative pathway through recruitment of C3b and Factor B, while the plasma properdin does not. Therefore, we have included neutrophil-secreted properdin in our model, referred with an asterisk (properdin P*). In addition, neutrophil-derived properdin (P*) concentration was estimated based on [22,74]. Cell-bound complement protein concentrations were estimated based on [75]. Lastly, a delay of 10 minutes was incorporated into our model (for neutrophil-secreted properdin) to account for neutrophil transmigration from the vasculature to the site of infection [76].

Production of complement components was neglected in our model (with the exception of properdin P*), since we examined only the short time frame following pathogen recognition. All equations were solved using the ode15s solver in Matlab (Mathworks, Natick, MA). Since complement activation is localized on cell surfaces, we included representative pathogen and host cell species in our model. We used human erythrocytes as a model host cell, due to the availability of information regarding cell dimensions [77] and densities of complement receptors [75]. *Escherichia coli* was used as the representative pathogen cell. Cell number densities were selected based on the homeostatic number of erythrocytes per milliliter of blood (5×10^9^ per ml) and the typical number of bacteria found in blood during sepsis (10^2^–10^5^ per ml) [78,79]. In order to model complement deposition on cell surfaces, we first calculated a theoretical maximum number of binding sites for complement proteins and complexes, based on the dimensions of cells and the various complement species. For instance, we acquired the area that C3b would occupy on the surface from literature and used this area as basis to estimate the area occupied by C5b-7 (due to the lack of literature data) [80]. The number of binding sites was translated into a concentration, by multiplying the cell number density. Reaction rate constants for complement species with available cell binding sites were derived based on rates of diffusion in blood and species inactivation (i.e. C3b thioester hydrolysis and C5b-7 spontaneous micelle formation) (S2 Text). Furthermore, in order to account for complement amplification and deposition on pathogen surfaces, an apparent concentration of nascent C3b and cell binding sites was calculated, based on the hemispheric region in which C3b molecules can diffuse before thioester hydrolysis (the radius and corresponding volume of this region was calculated based on thioester half life). In this hemispheric region, scaling factors were calculated so that in this way a nascent C3b molecule from a surface-bound convertase is much more likely to attach to the cell surface compared to a nascent C3b molecule from a fluid phase convertase. Nascent C5b-7 was treated in the same way, according to the half life of the C5b-7 hydrophilic-amphipathic transition. All the parameter data can be found in S1 Table. Reaction rate constants that were acquired from literature are cited in S1 Table, including those determined through optimization procedure. Parameters attained through structural/functional homologous interactions and association/dissociation constants are marked in S1 Table as estimation, while parameters that are completely unknown are marked as assumptions. Lastly, reaction rate constants calculated through the diffusion/species inactivation are also marked as such.

### Model development

#### Initiation (fluid phase)

Activation of the alternative pathway initiates in plasma by the spontaneous hydrolysis and activation of the complement component C3. Turnover of C3 is the critical step in the activation of the alternative pathway. The tick-over of C3 generates C3(H_2_O), a C3b-like molecule that has a hydrolyzed internal thioester bond (Fig 1). This molecule then associates with Factor B to produce C3(H_2_O)B. This initial convertase complex is prone to enzymatic cleavage by Factor D, resulting in the production of C3(H_2_O)Bb. This protease is a short-lived fluid phase convertase that cleaves C3 into a smaller fragment C3a and larger fragment C3b (Fig 1). C3a plays an important role in mediating inflammatory responses including vasodilation, histamine release from mast cells, smooth muscle contraction, and also antimicrobial activity during complement activation [81,82], and is one of the terminal complement activation products in our model. The larger product of C3 cleavage, nascent fluid-phase C3b (nfC3b), is a very reactive protein due to its exposed internal thioester bond that is capable of indiscriminately binding to different surfaces via covalent bond [29–31]. However, the short half-life of the thioester (60 μsec) ensures most of the C3b produced interacts with water and form the fluid phase C3b (fC3b), which loses its ability to covalently attach to cell surfaces [32].

#### Amplification (pathogen surface)

If the nascent C3b reacts and forms a covalent bond on the pathogen surface (pC3b), it also follows the same reaction scheme of the fluid phase C3(H_2_O) by associating with Factor B and leading to the cleavage by Factor D to yield the surface-bound C3 convertase, C3bBb. This complement enzyme sets in motion the amplification loop of the alternative pathway by cleaving native C3 and releasing C3a and nascent C3b fragments. Since surface-bound convertases produce C3b near the cell surface, these nascent C3b molecules (npC3b) have a high propensity for binding back to the same cell surface. The newly produced C3b recruits Factor B and Factor D, further building C3bBb convertases on the surface of pathogens (Fig 1). However, the C3bBb complex is rather unstable and has a half-life of about 90 seconds [41]. A positive regulatory protein known as properdin (P), associates with C3bBb, thus stabilizing and increasing the half-life of the convertase 10-fold [83]. Properdin has however recently reemerged as more than a stabilizing factor for the C3 convertase by the multiple roles it plays during complement activity. It has been demonstrated that properdin not only binds to formed alternative pathway C3 convertase but also once bound to a surface could accelerate the association of C3b and Factor B, subsequently forming the properdin stabilized convertase C3bBbP [18]. The latter study has also shown a model in which properdin, a dimer, trimer, or tetramer, bound to C3b through one site, can use its other vacant binding sites to recruit C3b, C3bB, and C3bBb, thus directing complement activation. Furthermore, properdin has been shown to be a pattern-recognition molecule that distinguishably binds to specific cell surfaces and serves as a platform for de novo convertase assembly to initiate the alternative pathway C3bBbP [14–22]. However, for our studies we focused on neutrophil-secreted properdin that has been shown to bind on the surface of the neutrophil [22,66]. Although these findings demonstrate properdin as a molecule that distinguishably binds to specific cell surfaces, purified physiological forms of properdin do not bind to certain surfaces [17,84]. To compensate for the differences, our model has included two kinds of properdin, in which the neutrophil-secreted properdin (P*) has the ability of attaching to surfaces and recruit C3b, while plasma properdin (P) does not. In addition, our model incorporates a delay of 10 minutes for P* release, to account for neutrophil transmigration from the vasculature to the site of infection [76].

#### Termination (pathogen)

The accumulation of C3b molecules on the surface of a pathogen leads to opsonization, thus rendering the pathogen subject to phagocytosis. However, C3b also binds with the C3 convertase to form a C5 convertase (C3b_2_Bb) of the alternative pathway. This protein complex cleaves C5, resulting to C5a and C5b on which the latter product remains loosely bound to the convertase while C5a is released in the fluid phase. Like C3a, belonging to a group of complement anaphylatoxins, C5a is a potent mediator of inflammatory responses [85–87], and is considered a terminal complement activation product in our model. C5b initiates a cascade of biochemical reactions that lead to the formation of the membrane attack complex (MAC). Following C5 cleavage, C5b undergoes a conformational transition, during which C6 binds. The half-life of C5b was shown to be 2.3 minutes before it irreversibly loses binding capabilities to C6 [88]. C5 convertase-bound C5b-6 may then bind C7, at which point the C5b-7 complex dissociates and undergoes a hydrophilic-amphiphilic transition, generating a membrane binding site. The formed metastable C5b-7 is released into the fluid phase and has the ability to insert itself into the lipid bilayer but can also form inactive protein micelles in the absence of membranes [85,86,89]. Once C5b-7 attaches to a membrane, it establishes a binding site for C8 [90–92]. Complement protein C8 will then inserts itself into the lipid bilayer of the pathogen and also induce the oligomerization of C9 to form a MAC complex, which is responsible for cell lysis [85,93].

#### Regulation (host cell and fluid phase)

The complement system is tightly regulated to prevent damage to host cells. Several soluble and cell-bound regulator proteins bind to complement proteins and complexes, inhibiting activation at several points in the activation cascade. Complement amplification is regulated through inhibition of convertase formation (mediated by FH and CR1), dissociation of existing convertases (mediated by FH, CR1, and DAF), cleavage of C3b into iC3b (mediated by FI using CR1 or FH as a cofactor), and subsequent cleavage of iC3b to C3dg (mediated by FI using CR1 as a cofactor). In addition, the terminal pathway is regulated by soluble vitronectin (Vn) and clusterin (Cn), and cell-bound (CD59) complement regulators. MCP, another cell-bound C3b inactivator, is not considered in our model, since it is not expressed on erythrocyte surfaces.

### Sensitivity analysis

Sensitivity analysis was implemented using the multi-parametric sensitivity analysis (MPSA) method [94]. The procedure of MPSA is described in the following steps. First, we selected all parameters to be tested. This includes 17 initial concentrations and 74 kinetic parameters. Second, the range of each selected parameter was set large enough to cover all feasible variations (increasing and decreasing by a factor of 10 for the rate constants and by factor 5 for the concentrations). The parameter ranges used for the sensitivity can be found in S3 and S4 Tables. Third, for each selected parameter, a series of independent random numbers were generated with a uniform distribution within the defined range. The Latin hypercube sampling method was used to sample 2000 random parameter vectors due to the large number of rate constants. This ensures maximal sampling through each parameter dimension while also computationally managing large number of rate constants [94,95]. The Matlab function ‘lhsdesign’ was used to produce the 2000 parameter vectors. Next, the model was simulated for each chosen set of parameter values to calculate the objective function values. The objective function is defined as the sum of squared errors between the observed and perturbed system output values

$$f_{obj}\left(k\right)=\sum_{i}^{n}\;{\left(x_{obj}\left(i\right)-x_{cal}\left(i,k\right)\right)}^{2}$$

where *x*_*obj*_*(i)* and *x*_*cal*_*(i*,*k)* denote the observed values calculated from the reference parameter values at the *i*th sampling time and perturbed system output values at the *i*th sampling time for the parameter variation set *k*, respectively. The output values denote the surface occupation (pathogen/host cell) by complement proteins and *n* is the sampling time point, which is set to be each time point through the simulations. Then it was determined whether the parameter set is acceptable or unacceptable by comparing the objective function value to a given threshold. If the objective function value is greater than or less than the threshold, the parameter set of values is classified as unacceptable or acceptable, respectively. Since MPSA results are not affected by the choice of subjective threshold [94,96], here we used a 50% divisions of 2000 sorted objective functions. Lastly, we statistically evaluated the parameter sensitivity by quantitatively comparing the distributions of acceptable and unacceptable values. The cumulative frequency is computed for each selected parameter with both acceptable and unacceptable cases. Sensitivity was evaluated by the measure of separation between the two cumulative frequency distributions of acceptable and unacceptable cases using Kolmogorov-Smirnov (K-S) statistic

$$K-S=\begin{array}{c} max\\ x \end{array}\left|C_{a}\left(x\right)-C_{u}\left(x\right)\;\right|$$

where *C*_a_ and *C*_u_ correspond to the cumulative frequency functions that are acceptable and unacceptable cases, and *x* is the given parameter. Large values of K-S test indicate the model output is sensitive to the given parameter variations.

## Supporting Information

S1 Fig Extended time profile for complement deposition on pathogen and host surfaces. This figure is similar to Fig 2, but the time profile is shown at the extended timeframe of 180 minutes.
(TIF)

S2 Fig Extended time profile for the concentration of assembled fluid phase convertases, C3(H_2_O)Bb and C3bBb, of the alternative pathway. This figure is similar to Fig 3, but the time profile is shown at the extended timeframe of 180 minutes.
(TIF)

S3 Fig Extended time profile for the formation of C3 cleaving enzymes on the surface of host cells and pathogens. This figure is similar to Fig 4, but the time profile is shown at the extended timeframe of 180 minutes.
(TIF)

S4 Fig Extended time profile for the production of C3a and C5a. This figure is similar to Fig 5, but the time profile is shown at the extended timeframe of 180 minutes.
(TIF)

S5 Fig Extended time profile for the formation of MAC pores is characterized by three phases: lag phase, production phase, and a steady state phase. This figure is similar to Fig 7, but the time profile is shown at the extended timeframe of 180 minutes.
(TIF)

S6 Fig Extended time profiles for complement regulators Factor H, CR1, DAF, vitronectin, clusterin, and CD59. This figure is similar to Fig 9, but the time profiles are shown at the extended timeframe of 180 minutes.
(TIF)

S1 Table Kinetic Rate Constants. (PDF)

S2 Table Complement Protein Concentrations and Molecular Masses. (PDF)

S3 Table Range of Complement Protein Concentrations Implemented in Sensitivity Analysis. (PDF)

S4 Table Range of Kinetic Rate Constants Implemented in Sensitivity Analysis. (PDF)

S1 Text Mathematical Model. System of 107 Ordinary Differential Equations.
(PDF)

S2 Text Modeling of Complement Amplification on Cell Surfaces. (PDF)
