# Supplementary figures and images for: Quantitative Modeling of the Alternative Pathway of the Complement System
Source: PLoS One. 2016 Mar 31;11(3):e0152337. doi: 10.1371/journal.pone.0152337 (PMC4816337; doi:10.1371/journal.pone.0152337)

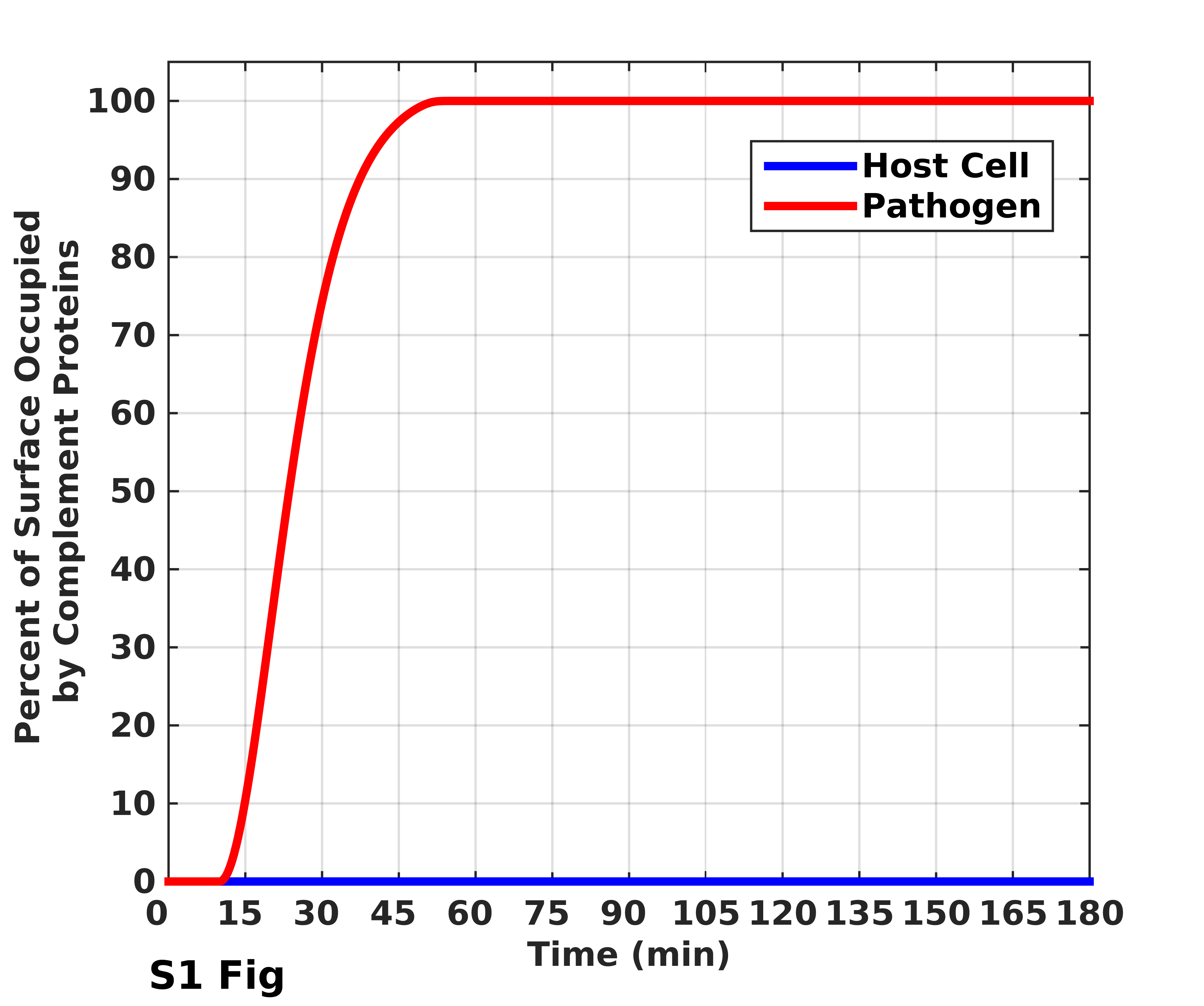

Supplement: S1 Fig — This figure is similar to Fig 2, but the time profile is shown at the extended timeframe of 180 minutes. (TIF) [file pone.0152337.s001.tif]

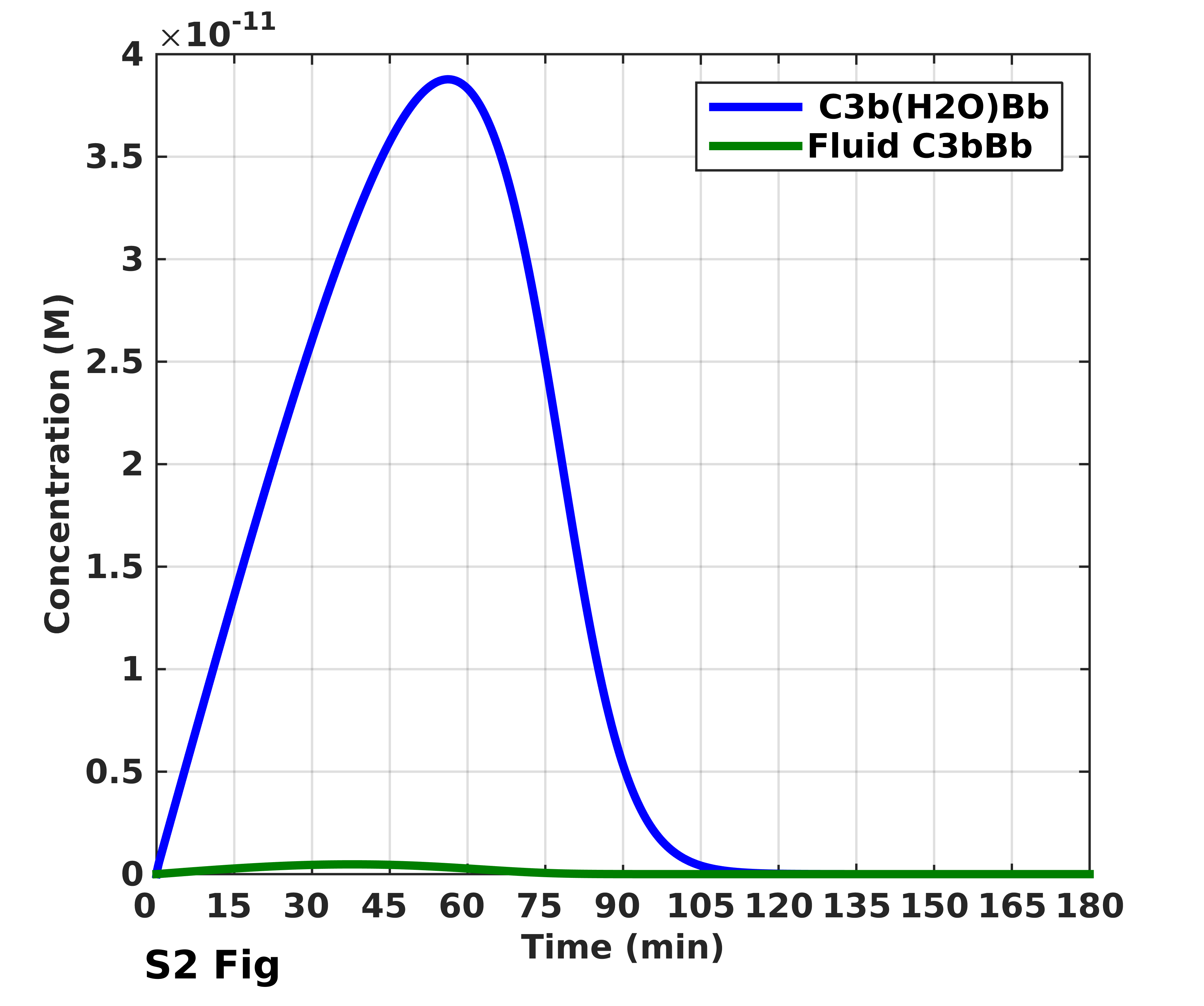

Supplement: S2 Fig — This figure is similar to Fig 3, but the time profile is shown at the extended timeframe of 180 minutes. (TIF) [file pone.0152337.s002.tif]

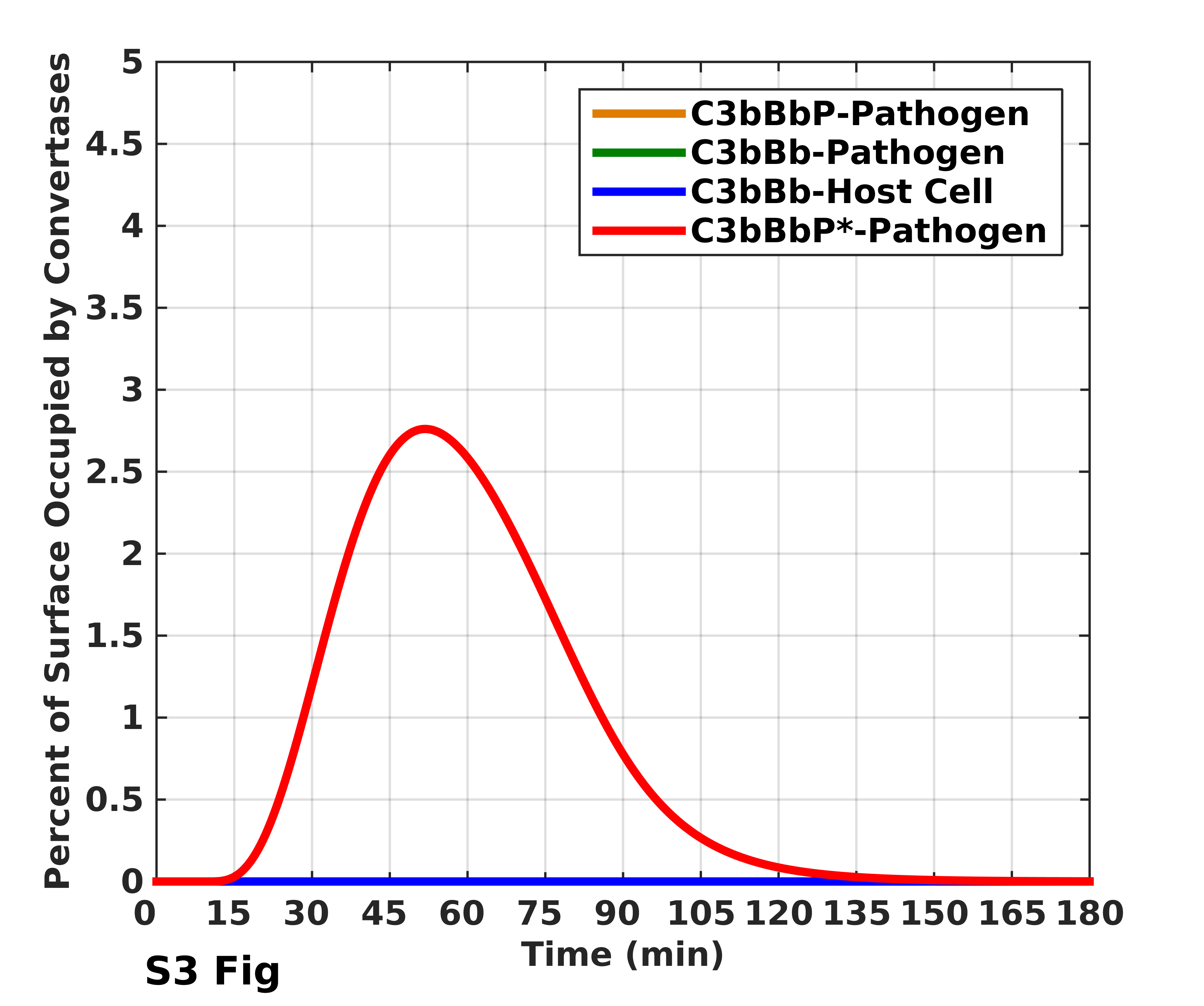

Supplement: S3 Fig — This figure is similar to Fig 4, but the time profile is shown at the extended timeframe of 180 minutes. (TIF) [file pone.0152337.s003.tif]

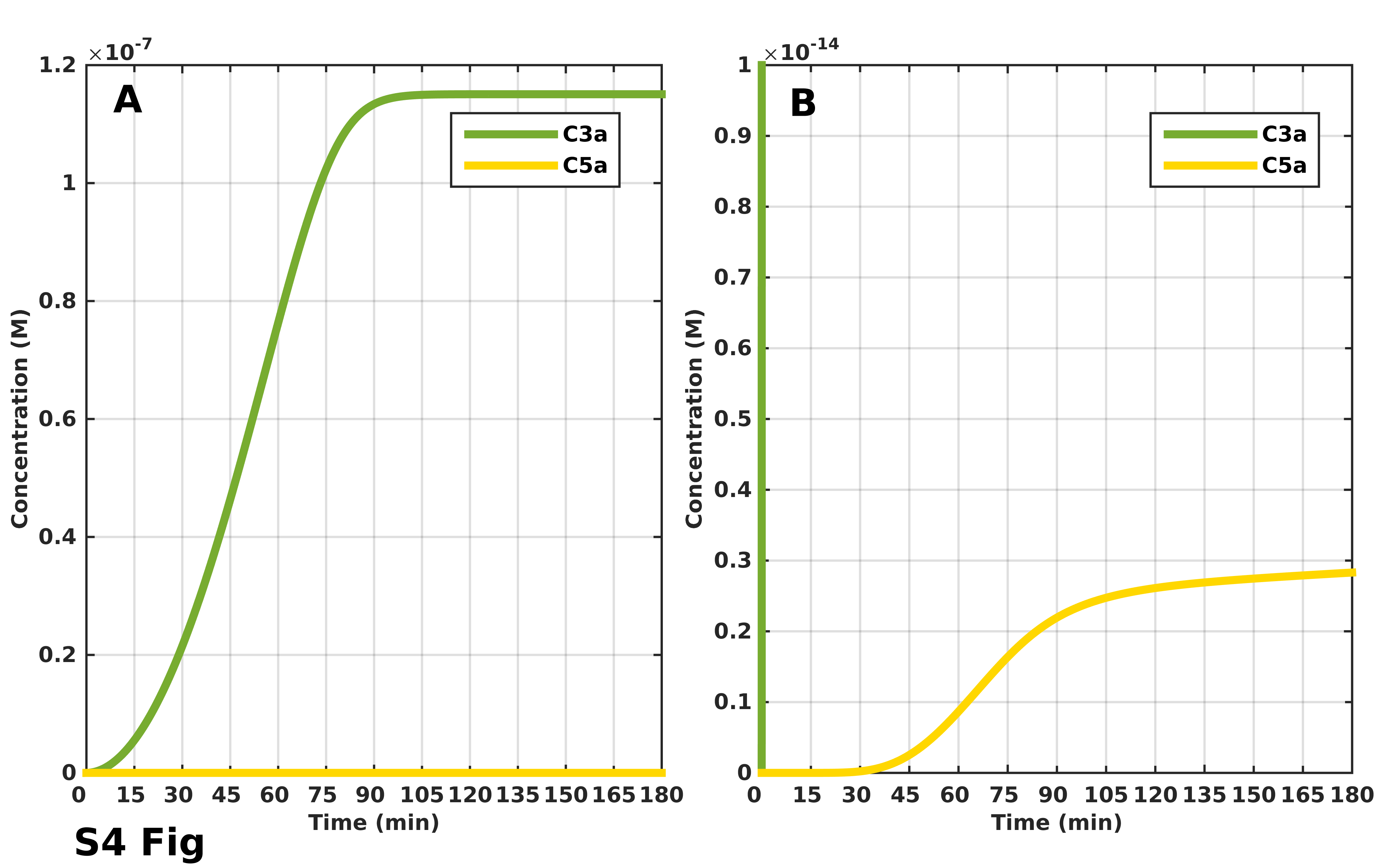

Supplement: S4 Fig — This figure is similar to Fig 5, but the time profile is shown at the extended timeframe of 180 minutes. (TIF) [file pone.0152337.s004.tif]

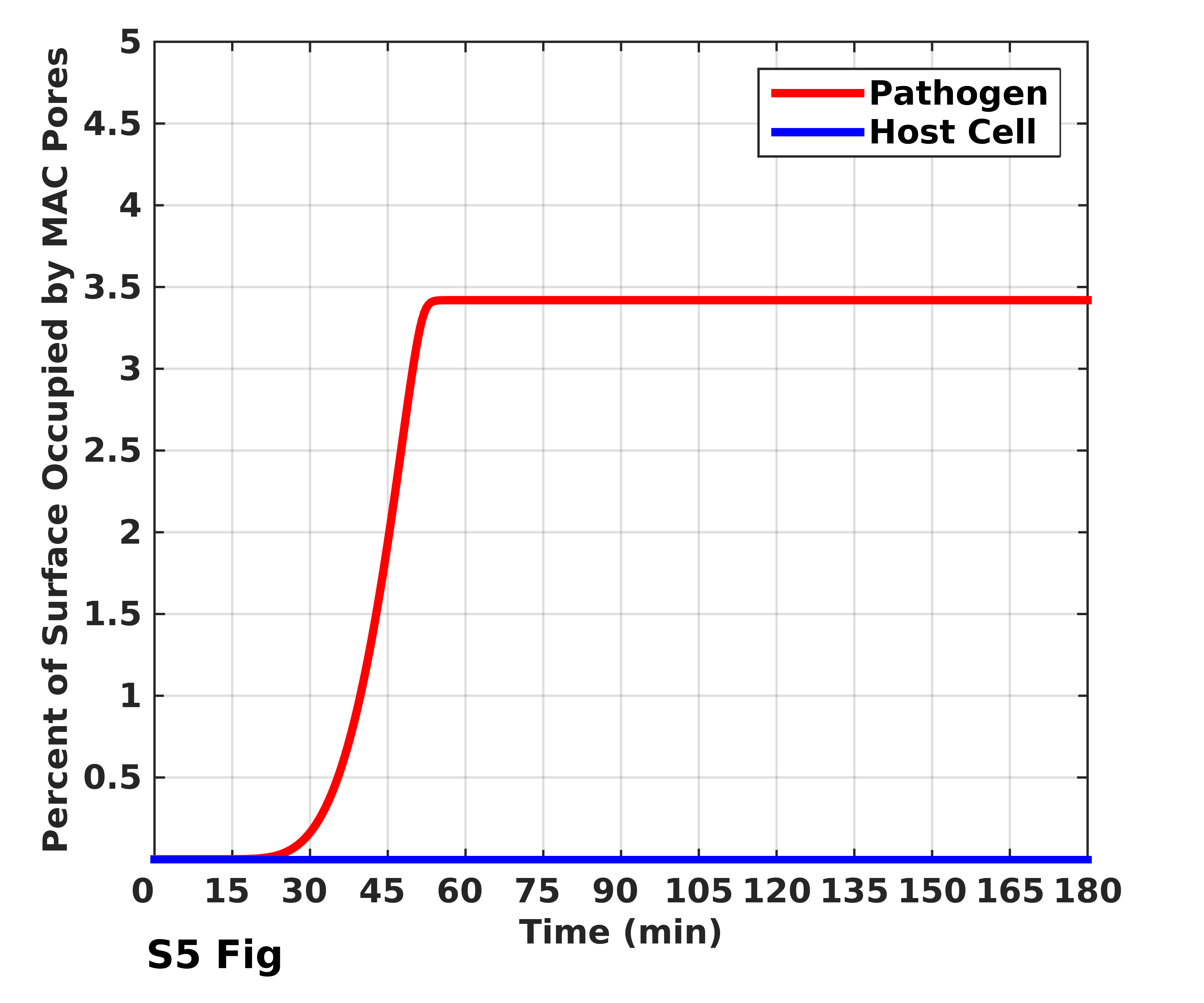

Supplement: S5 Fig — This figure is similar to Fig 7, but the time profile is shown at the extended timeframe of 180 minutes. (TIF) [file pone.0152337.s005.tif]

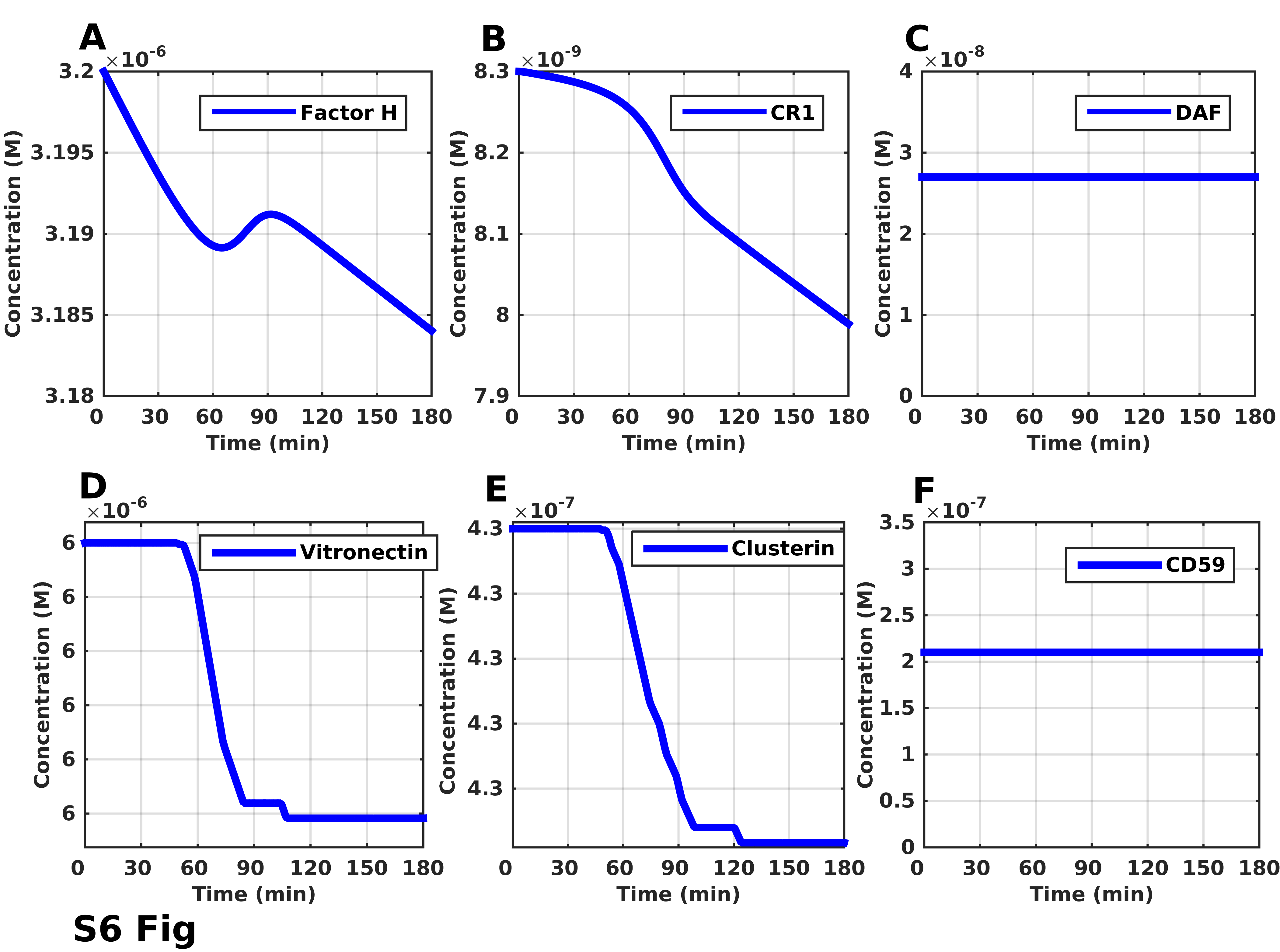

Supplement: S6 Fig — This figure is similar to Fig 9, but the time profiles are shown at the extended timeframe of 180 minutes. (TIF) [file pone.0152337.s006.tif]
