# Supplementary material for: Quantitative Modeling of the Alternative Pathway of the Complement System
Source: PLoS One. 2016 Mar 31;11(3):e0152337. doi: 10.1371/journal.pone.0152337 (PMC4816337; doi:10.1371/journal.pone.0152337)
Supplement: S1 Table — (PDF) [file pone.0152337.s007.pdf]

S1 Table. Kinetic Rate Constants.

**Biochemical Reaction**	**Rate Constant**	**Value**	**Source**[a]
Hydrolysis of $C3(H_2O)$	$k^{+}_{\mathrm{C3(H_2O)}}$	8.3×10^{-7} s^{-1}	[1]
Association of Factor B to $C3(H_2O)$	$k^{+}_{\mathrm{C3(H_2O)B}}$	21.3×10^{4} $M^{-1}s^{-1}$	Estimation structurally/functionally homologous proteins
Dissociation of complex $C3(H_2O)B$	$k^{-}_{\mathrm{C3(H_2O)B}}$	15.5×10^{-2} s^{-1}	Estimation structurally/functionally homologous proteins
Association of Factor H to $C3(H_2O)$	$k^{+}_{\mathrm{C3(H_2O)H}}$	5.2×10^{6} $M^{-1}s^{-1}$	Estimation structurally/functionally homologous proteins
Dissociation of complex $C3(H_2O)H$	$k^{-}_{\mathrm{C3(H_2O)H}}$	32.5 s^{-1}	Estimation structurally/functionally homologous proteins
Dissociation of complex $C3(H_2O)Bb$	$k^{-}_{\mathrm{C3(H_2O)Bb}}$	9.0×10^{-3} s^{-1}	[2]
Association of Factor B to C3b	k^{+}_{C3bB}	21.3×10^{4} $M^{-1}s^{-1}$	[3]
Dissociation of complex C3bB	k^{-}_{C3bB}	15.5×10^{-2} s^{-1}	[3]
Dissociation of complex C3bBb	k^{-}_{C3bBb}	7.7×10^{-3} s^{-1}	[2]
Dissociation of complex C3bBbP on pathogen	k^{-}_{C3bBbP}	7.7×10^{-4} s^{-1}	[4]
Association of properdin* to C3b on pathogen	k^{+}_{C3bP}	3.0×10^{6} $M^{-1}s^{-1}$	[5] Optimization

Dissociation of complex C3bP* on pathogen	k^{-}_{C3bP}	5.0×10^{-4} s^{-1}	[4]
Association of npC3b to properdin* on pathogen	k^{+}_{C3bP}	3.0×10^{6} $M^{-1}s^{-1}$	[5] Optimization
Dissociation of complex npC3bP* on pathogen	k^{-}_{C3bP}	5.0×10^{-4} s^{-1}	[4]
Attachment of nfC3b to host cell and pathogen	$k^{+}_{\mathrm{C3b_{surface}}}$	4.2×10^{8} $M^{-1}s^{-1}$	Calculated based on rates of diffusion in blood
Association of nfC3b, nhC3b, and npC3b to water	k^{+}_{fC3b}	4.2×10^{8} $M^{-1}s^{-1}$	Calculated based on rates of diffusion in blood
Attachment of nhC3b to host cell	k^{+}_{hC3b}	4.2×10^{8} $M^{-1}s^{-1}$	Calculated based on rates of diffusion in blood
Attachment of npC3b to pathogen	k^{+}_{pC3b}	4.2×10^{8} $M^{-1}s^{-1}$	Calculated based on rates of diffusion in blood
Rate of release of properdin* from neutrophil	$k^{+}_{\mathrm{p*_{released}}}$	1.0×10^{-3} s^{-1}	Assumption
Attachment of properdin* to pathogen	$k^{+}_{\mathrm{P*_{surface}}}$	3.0×10^{6} $M^{-1}s^{-1}$	Assumption
Dissociation of properdin* from pathogen	$k^{-}_{\mathrm{P*_{surface}}}$	5.0×10^{-4} s^{-1}	Assumption
Association of properdin to iC3b on pathogen	k^{+}_{iC3bP}	3.0×10^{6} $M^{-1}s^{-1}$	[5] Optimization
Dissociation of complex from iC3b on pathogen	k^{-}_{iC3bP}	3.8×10^{-4} s^{-1}	[4]
Association of Factor H to fluid C3b	k^{+}_{C3bH}	5.2×10^{6} $M^{-1}s^{-1}$	[6]

Dissociation of complex C3bH in fluid	k^{-}_{C3bH}	32.5 s^{-1}	[6]
Association of Factor H to C3b on host cell	k^{+}_{C3bH}	5.2×10^{6} $M^{-1}s^{-1}$	[6]
Dissociation of complex C3bH on host cell	k^{-}_{C3bH}	3.25 s^{-1}	[6]
Association of CR1 to C3b	k^{+}_{C3bCR1}	1.2×10^{4} $M^{-1}s^{-1}$	Estimation based on association constant (0.5×10^{6} — 2×10^{6} M^{-1}) [7–9]
Dissociation of complex C3bCR1	k^{-}_{C3bCR1}	1.0×10^{-2} s^{-1}	Estimation based on association constant (0.5×10^{6} — 2×10^{6} M^{-1}) [7–9]
Association of CR1 to $C3(H_2O)$	$k^{+}_{\mathrm{C3(H_2O)CR1}}$	1.2×10^{4} $M^{-1}s^{-1}$	Estimation structurally/functionally homologous proteins
Dissociation of complex $C3(H_2O)CR1$	$k^{-}_{\mathrm{C3(H_2O)CR1}}$	1.0×10^{-2} s^{-1}	Estimation structurally/functionally homologous proteins
Association of DAF to C3 convertase on host cell	$k^{+}_{\mathrm{C3bBbDAF}}$	2.0×10^{3} $M^{-1}s^{-1}$	Estimation based on dissociation constant (10^{-5} M^{-1}) [10]
Decay of C3 convertase by inhibitor DAF on host cell	$k^{-}_{\mathrm{C3bBbDAF}}$	7.7×10^{-2} s^{-1}	Assumptions
Decay of C3 convertase by inhibitor CR1 on host cell	$k^{-}_{\mathrm{C3bBbCR1}}$	7.7×10^{-2} s^{-1}	Assumption
Decay of C3 convertase by inhibitor Factor H on host cell	k^{-}_{C3bBbH}	7.7×10^{-2} s^{-1}	Assumption

Association of iC3b to CR1	k^{+}_{iC3bCR1}	2.0×10^{3} $M^{-1}s^{-1}$	Estimation<br>based on association constant<br>(2×10^{5} M^{-1})<br>[8,11]
Dissociation of complex iC3bCR1	k^{-}_{iC3bCR1}	1.0×10^{-2} s^{-1}	Estimation<br>based on association constant<br>(2×10^{5} M^{-1})<br>[8,11]
Association of C3b to C3bBb	$k^{+}_{\mathrm{C3bBbC3b}}$	3.5×10^{6} $M^{-1}s^{-1}$	[5]<br>Optimization
Dissociation of complex C3bBbC3b	$k^{-}_{\mathrm{C3bBbC3b}}$	3.8×10^{-3} s^{-1}	[12]
Association of C5 to C3bBbC3b	$k^{+}_{\mathrm{C3bBbC3bC5}}$	5.0×10^{6} $M^{-1}s^{-1}$	[13]
Dissociation of complex C3bBbC3bC5	$k^{-}_{\mathrm{C3bBbC3bC5}}$	1.0×10^{-2} s^{-1}	[13]
Dissociation of complex C3bBbC3bC5b	k^{-}_{C5b}	3.8×10^{-2} s^{-1}	[14]
Association of C6 to C3bBbC3bC5b	$k^{+}_{\mathrm{C3bBbC3bC5C6}}$	6.0×10^{4} $M^{-1}s^{-1}$	[5,15]
Dissociation of complex C3bBbC3bC5bC6	$k^{-}_{\mathrm{C3bBbC3bC5bC6}}$	9×10^{-8} s^{-1}	[5,15]
Association of C7 to C3bBbC3bC5bC6	k^{+}_{C5b7}	7.3×10^{5} $M^{-1}s^{-1}$	[5,15,16]
Dissociation of complex C3bBbC3bC5bC6C7	k^{-}_{C5b7}	1.5×10^{-6} — 2.1×10^{-7} s^{-1}	[5,15,16]
Attachment of C5b7 to host cell and pathogen	$k^{+}_{\mathrm{C5b7}_{\mathrm{surface}}}$	4.2×10^{8} $M^{-1}s^{-1}$	Calculated<br>based on rates of diffusion in blood

Formation of C5b7 micelle in fluid	k^{+}_{micelle}	69.3 s^{-1}	[16]
Association of C8 to C5b7	k^{+}_{C5b8}	1.1×10^{6} $M^{-1}s^{-1}$	[5,15,16]
Dissociation of complex C5b8	k^{-}_{C5b8}	9.8×10^{-7} s^{-1}	[5,15,16]
Association of C9 to C5b8	k^{+}_{C5b9}	2.8×10^{6} $M^{-1}s^{-1}$	[5,15,16]
Dissociation of complex C5b9	k^{-}_{C5b9}	2.8×10^{-6} — 1.4×10^{-7} s^{-1}	[5,15,16]
Association of Cn to C5b7	k^{+}_{CnC5b7}	4.1×10^{5} $M^{-1}s^{-1}$	Estimation structurally/functionally homologous proteins
Dissociation of complex CnC5b7	k^{-}_{CnC5b7}	4.0×10^{-3} s^{-1}	Estimation structurally/functionally homologous proteins
Association of Cn to C5b8	k^{+}_{CnC5b8}	4.1×10^{5} $M^{-1}s^{-1}$	[5] Optimization
Dissociation of complex CnC5b8	k^{-}_{CnC5b8}	4.0×10^{-3} s^{-1}	[5] Optimization
Association of Vn to C5b7	k^{+}_{VnC5b7}	2.4×10^{5} $M^{-1}s^{-1}$	[5,17]
Dissociation of complex VnC5b7	k^{-}_{VnC5b7}	2.0×10^{-3} s^{-1}	Assumption
Association of CD59 to C5b9	$k^{+}_{\mathrm{CD59C5b9}}$	1.0×10^{6} $M^{-1}s^{-1}$	Assumption
Dissociation of complex CD59C5b9	$k^{-}_{\mathrm{CD59C5b9}}$	2.0×10^{-4} s^{-1}	Assumption

Cleavage of C3 by C3 convertase, $C3(H_20)Bb$	k_{cat} $C3(H_2O)Bb$<br>K_M $C3(H_2O)Bb$	1.8 s^{-1}<br>5.9×10^{-6} M	Estimation structurally/functionally homologous proteins
Cleavage of C3 by C3 convertase, C3bBb	k_{cat} C3bBb<br>K_M C3bBb	1.8 s^{-1}<br>5.9×10^{-6} M	[2]
Cleavage of C3 by C3 convertase, C3bBbP	k_{cat} C3bBbP<br>K_M C3bBbP	3.1 s^{-1}<br>1.8×10^{-6} M	[5] Optimization
Activation of complex C3bB by enzyme Factor D	k_{cat} C3bB<br>K_M C3bB	2.1 s^{-1}<br>0.1×10^{-6} M	[5] Optimization
Activation of complex $C3(H_2O)B$ by enzyme Factor D	k_{cat} $C3(H_2O)B$<br>K_M $C3(H_2O)B$	2.1 s^{-1}<br>0.1×10^{-6} M	Estimation structurally/functionally homologous proteins
Cleavage of C3b by inhibitor Factor I	k_{cat} C3bH<br>K_M C3bH	1.3 s^{-1}<br>2.5×10^{-7} M	[6]
Cleavage of C5 by the C5 convertase, C3bBbC3b and C3bBbC3bP	k_{cat} C3bBbC3b<br>K_M C3bBbC3b	4.8 s^{-1}<br>1.8×10^{-6} M	[5,18]

[a]Details on estimations and assumptions are given in Methods.

References

1. Pangburn MK, Schreiber RD, Müller-Eberhard HJ. Formation of the initial C3 convertase of the alternative complement pathway. Acquisition of C3b-like activities by spontaneous hydrolysis of the putative thioester in native C3. J Exp Med. 1981;154: 856–867.
2. Pangburn MK, Müller-Eberhard HJ. The C3 convertase of the alternative pathway of human complement. Enzymic properties of the bimolecular proteinase. Biochem J. 1986;235: 723–730.
3. Chen H, Ricklin D, Hammel M, Garcia BL, McWhorter WJ, Sfyroera G, et al. Allosteric inhibition of complement function by a staphylococcal immune evasion protein. Proc Natl Acad Sci. 2010;107: 17621–17626. doi:10.1073/pnas.1003750107

4. Hourcade DE. The Role of Properdin in the Assembly of the Alternative Pathway C3 Convertases of Complement. J Biol Chem. 2006;281: 2128–2132. doi:10.1074/jbc.M508928200
5. Korotaevskiy AA, Hanin LG, Khanin MA. Non-linear dynamics of the complement system activation. Math Biosci. 2009;222: 127–143. doi:10.1016/j.mbs.2009.10.003
6. Pangburn MK, Mueller-Eberhard HJ. Kinetic and thermodynamic analysis of the control of C3b by the complement regulatory proteins factors H and I. Biochemistry (Mosc). 1983;22: 178–185. doi:10.1021/bi00270a026
7. Grattone ML, Villiers CL, Villiers M-B, Drouet C, Marche PN. Co-operation between human CR1 (CD35) and CR2 (CD21) in internalization of their C3b and iC3b ligands by murine-transfected fibroblasts. Immunology. 1999;98: 152–157. doi:10.1046/j.1365-2567.1999.00839.x
8. Becherer JD, Lambris JD. Identification of the C3b receptor-binding domain in third component of complement. J Biol Chem. 1988;263: 14586–14591.
9. Arnaout MA, Dana N, Melamed J, Medicus R, Colten HR. Low ionic strength or chemical cross-linking of monomeric C3b increases its binding affinity to the human complement C3b receptor. Immunology. 1983;48: 229–237.
10. Claire L Harris DMP. Decay-accelerating factor must bind both components of the complement alternative pathway C3 convertase to mediate efficient decay. J Immunol Baltim Md 1950. 2007;178: 352–9. doi:10.4049/jimmunol.178.1.352
11. Gordon DL, Johnson GM, Hostetter MK. Characteristics of iC3b binding to human polymorphonuclear leucocytes. Immunology. 1987;60: 553–558.
12. Muller-Eberhard HJ. The Membrane Attack Complex of Complement. Annu Rev Immunol. 1986;4: 503–528. doi:10.1146/annurev.iy.04.040186.002443
13. Rawal N, Pangburn MK. Functional Role of the Noncatalytic Subunit of Complement C5 Convertase. J Immunol. 2000;164: 1379–1385. doi:10.4049/jimmunol.164.3.1379
14. Cooper NR, Müller-Eberhard HJ. The reaction mechanism of human C5 in immune hemolysis. J Exp Med. 1970;132: 775–793.
15. Li CKN, Levine RP. Rate process in the final stage of complement hemolysis. Immunochemistry. 1977;14: 421–428. doi:10.1016/0019-2791(77)90167-7
16. Podack ER, Biesecker G, Kolb WP, Müller-Eberhard HJ. The C5b-6 complex: reaction with C7, C8, C9. J Immunol Baltim Md 1950. 1978;121: 484–490.
17. McDonald JF, Nelsestuen GL. Potent inhibition of terminal complement assembly by clusterin: characterization of its impact on C9 polymerization. Biochemistry (Mosc). 1997;36: 7464–7473. doi:10.1021/bi962895r
18. Rawal N, Pangburn MK. Formation of High-Affinity C5 Convertases of the Alternative Pathway of Complement. J Immunol. 2001;166: 2635–2642. doi:10.4049/jimmunol.166.4.2635
