# Supplementary material for: Quantitative Modeling of the Alternative Pathway of the Complement System
Source: PLoS One. 2016 Mar 31;11(3):e0152337. doi: 10.1371/journal.pone.0152337 (PMC4816337; doi:10.1371/journal.pone.0152337)
Supplement: S2 Table — (PDF) [file pone.0152337.s008.pdf]

S2 Table. Complement Protein Concentrations and Molecular Masses.

Complement Proteins	Molecular Mass (kDa)	Concentration (µM)	Source
C3	185	5.4	[1,2]
C5	191	0.37	[3]
C6	120	0.54	[3]
C7	110	0.50	[3]
C8	151	0.36	[3]
C9	71	0.90	[3]
Factor B	93	2.2	[1]
Factor D	24	0.083	[1]
Factor I	88	0.4	[1]
Properdin	53	0.47	[1]
Properdin* (Neutrophil-Secreted)	53	0.0009 Estimation	[4]
Factor H	155	3.2	[1]
CR1	190	0.0083	[1,5]
DAF	70	0.027	[1,5]
Vitronectin	83	6.0	[3]
Clusterin	80	0.43	[6]
CD59	18	0.21	[7]

References

1. Zipfel PF. Complement: Alternative Pathway. eLS. John Wiley & Sons, Ltd; 2001. Available: http://onlinelibrary.wiley.com/doi/10.1038/npg.els.0000509/abstract

2. Morgan BP, editor. The Complement System:An Overview - Springer. Humana Press; 2000. Available: http://link.springer.com/protocol/10.1385%2F1-59259-056-X%3A1

3. Muller-Eberhard HJ. The Membrane Attack Complex of Complement. Annu Rev Immunol. 1986;4: 503–528. doi:10.1146/annurev.iy.04.040186.002443

4. Wirthmueller U, Dewald B, Thelen M, Schäfer MK, Stover C, Whaley K, et al. Properdin, a positive regulator of complement activation, is released from secondary granules of stimulated peripheral blood neutrophils. J Immunol Baltim Md 1950. 1997;158: 4444–4451.

5. Seya T. Human Regulator of Complement Activation (RCA) Gene Family Proteins and Their Relationship to Microbial Infection. Microbiol Immunol. 1995;39: 295–305. doi:10.1111/j.1348-0421.1995.tb02205.x

6. Murphy BF, Kirszbaum L, Walker ID, d' Apice AJ. SP-40,40, a newly identified normal human serum protein found in the SC5b-9 complex of complement and in the immune deposits in glomerulonephritis. J Clin Invest. 1988;81: 1858–1864.

7. Meri S, Morgan BP, Davies A, Daniels RH, Olavesen MG, Waldmann H, et al. Human protectin (CD59), an 18,000-20,000 MW complement lysis restricting factor, inhibits C5b-8 catalysed insertion of C9 into lipid bilayers. Immunology. 1990;71: 1–9.
