# Supplementary material for: Quantitative Modeling of the Alternative Pathway of the Complement System
Source: PLoS One. 2016 Mar 31;11(3):e0152337. doi: 10.1371/journal.pone.0152337 (PMC4816337; doi:10.1371/journal.pone.0152337)
Supplement: S3 Table — (PDF) [file pone.0152337.s009.pdf]

S3 Table. Range of Complement Protein Concentrations Implemented in Sensitivity Analysis.

**Complement Proteins**	**Initial Concentration (μM)**	**Range of Variation (μM)**
C3	5.4	1.2 − 27.0
C5	0.37	0.074 − 1.9
C6	0.54	0.12 − 2.7
C7	0.50	0.10 − 2.5
C8	0.36	0.072 − 1.8
C9	0.90	0.18 − 4.5
Factor B	2.2	0.44 − 11
Factor D	0.083	0.017 − 0.42
Factor I	0.4	0.08 − 2.0
Properdin	0.47	0.094 − 2.4
Properdin* (Neutrophil-Secreted)	0.0009 Estimation	0.00018 − 0.005
Factor H	3.2	0.64 − 16.0
CR1	0.0083	0.002 − 0.042
DAF	0.027	0.005 − 0.14
Vitronectin	6.0	1.2 − 30
Clusterin	0.43	0.086 − 2.2
CD59	0.21	0.042 − 1.2
