# Supplementary material for: Quantitative Modeling of the Alternative Pathway of the Complement System
Source: PLoS One. 2016 Mar 31;11(3):e0152337. doi: 10.1371/journal.pone.0152337 (PMC4816337; doi:10.1371/journal.pone.0152337)
Supplement: S4 Table — (PDF) [file pone.0152337.s010.pdf]

S4 Table. Range of Kinetic Rate Constants Implemented in Sensitivity Analysis.

Biochemical Reaction	Rate Constant	Reference Value	Range of Variation
Hydrolysis of $C3(H_2O)$	$k^{+}_{\mathrm{C3(H_2O)}}$	8.3×10^{-7} s^{-1}	$8.3\times10^{-8} - 8.3\times10^{-6}$ s^{-1}
Association of Factor B to $C3(H_2O)$	$k^{+}_{\mathrm{C3(H_2O)B}}$	21.3×10^{4} $M^{-1}s^{-1}$	$21.3\times10^{3} - 21.3\times10^{5}$ $M^{-1}s^{-1}$
Dissociation of complex $C3(H_2O)B$	$k^{-}_{\mathrm{C3(H_2O)B}}$	15.5×10^{-2} s^{-1}	$15.5\times10^{-3} - 15.5\times10^{-1}$ s^{-1}
Association of Factor H to $C3(H_2O)$	$k^{+}_{\mathrm{C3(H_2O)H}}$	5.2×10^{6} $M^{-1}s^{-1}$	$5.2\times10^{5} - 5.2\times10^{7}$ $M^{-1}s^{-1}$
Dissociation of complex $C3(H_2O)H$	$k^{-}_{\mathrm{C3(H_2O)H}}$	32.5 s^{-1}	3.25 – 325 s^{-1}
Dissociation of complex $C3(H_2O)Bb$	$k^{-}_{\mathrm{C3(H_2O)Bb}}$	9.0×10^{-3} s^{-1}	$9.0\times10^{-4} - 9.0\times10^{-2}$ s^{-1}
Association of Factor B to C3b	k^{+}_{C3bB}	21.3×10^{4} $M^{-1}s^{-1}$	$21.3\times10^{3} - 21.3\times10^{5}$ $M^{-1}s^{-1}$
Dissociation of complex C3bB	k^{-}_{C3bB}	15.5×10^{-2} s^{-1}	$15.5\times10^{-3} - 15.5\times10^{-1}$ s^{-1}
Dissociation of complex C3bBb	k^{-}_{C3bBb}	7.7×10^{-3} s^{-1}	$7.7\times10^{-4} - 7.7\times10^{-2}$ s^{-1}
Dissociation of complex C3bBbP on pathogen	k^{-}_{C3bBbP}	7.7×10^{-4} s^{-1}	$7.7\times10^{-5} - 7.7\times10^{-3}$ s^{-1}
Association of properdin* to C3b on pathogen	k^{+}_{C3bP}	3.0×10^{6} $M^{-1}s^{-1}$	$3.0\times10^{5} - 3.0\times10^{7}$ $M^{-1}s^{-1}$

Dissociation of complex C3bP* on pathogen	k^{-}_{C3bP}	5.0×10^{-4} s^{-1}	$5.0\times10^{-5} - 5.0\times10^{-3}$ s^{-1}
Association of npC3b to properdin* on pathogen	k^{+}_{C3bP}	3.0×10^{6} $M^{-1}s^{-1}$	$3.0\times10^{5} - 3.0\times10^{7}$ $M^{-1}s^{-1}$
Dissociation of complex npC3bP* on pathogen	k^{-}_{C3bP}	5.0×10^{-4} s^{-1}	$5.0\times10^{-5} - 5.0\times10^{-3}$ s^{-1}
Attachment of nfC3b to host cell and pathogen	$k^{+}_{\mathrm{C3b_{surface}}}$	4.2×10^{8} $M^{-1}s^{-1}$	$4.2\times10^{7} - 4.2\times10^{9}$ $M^{-1}s^{-1}$
Association of nfC3b, nhC3b, and npC3b to water	k^{+}_{fC3b}	4.2×10^{8} $M^{-1}s^{-1}$	$4.2\times10^{7} - 4.2\times10^{9}$ $M^{-1}s^{-1}$
Attachment of nhC3b to host cell	k^{+}_{hC3b}	4.2×10^{8} $M^{-1}s^{-1}$	$4.2\times10^{7} - 4.2\times10^{9}$ $M^{-1}s^{-1}$
Attachment of npC3b to pathogen	k^{+}_{pC3b}	4.2×10^{8} $M^{-1}s^{-1}$	$4.2\times10^{7} - 4.2\times10^{9}$ $M^{-1}s^{-1}$
Rate of release of properdin* from neutrophil	$k^{+}_{\mathrm{p*released}}$	1.0×10^{-3} s^{-1}	$1.0\times10^{-4} - 1.0\times10^{-2}$ s^{-1}
Attachment of properdin* to pathogen	$k^{+}_{\mathrm{P*surface}}$	3.0×10^{6} $M^{-1}s^{-1}$	$3.0\times10^{5} - 3.0\times10^{7}$ $M^{-1}s^{-1}$
Dissociation of properdin* from pathogen	$k^{-}_{\mathrm{P*surface}}$	5.0×10^{-4} s^{-1}	$5.0\times10^{-5} - 5.0\times10^{-3}$ s^{-1}
Association of properdin to iC3b on pathogen	k^{+}_{iC3bP}	3.0×10^{6} $M^{-1}s^{-1}$	$3.0\times10^{5} - 3.0\times10^{7}$ $M^{-1}s^{-1}$
Dissociation of complex from iC3b on pathogen	k^{-}_{iC3bP}	3.8×10^{-4} s^{-1}	$3.8\times10^{-5} - 3.8\times10^{-3}$ s^{-1}
Association of Factor H to fluid C3b	k^{+}_{C3bH}	5.2×10^{6} $M^{-1}s^{-1}$	$5.2\times10^{5} - 5.2\times10^{7}$ $M^{-1}s^{-1}$

Dissociation of complex C3bH in fluid	k^{-}_{C3bH}	32.5 s^{-1}	3.25 – 325 s^{-1}
Association of Factor H to C3b on host cell	k^{+}_{C3bH}	5.2×10^{6} $M^{-1}s^{-1}$	$5.2 \times 10^{5} - 5.2 \times 10^{7}$ $M^{-1}s^{-1}$
Dissociation of complex C3bH on host cell	k^{-}_{C3bH}	3.25 s^{-1}	0.325 – 32.5 s^{-1}
Association of CR1 to C3b	k^{+}_{C3bCR1}	1.2×10^{4} $M^{-1}s^{-1}$	$1.2 \times 10^{3} - 1.2 \times 10^{5}$ $M^{-1}s^{-1}$
Dissociation of complex C3bCR1	k^{-}_{C3bCR1}	1.0×10^{-2} s^{-1}	$1.0 \times 10^{-3} - 1.0 \times 10^{-1}$ s^{-1}
Association of CR1 to $C3(H_2O)$	$k^{+}_{\mathrm{C3(H_2O)CR1}}$	1.2×10^{4} $M^{-1}s^{-1}$	$1.2 \times 10^{3} - 1.2 \times 10^{5}$ $M^{-1}s^{-1}$
Dissociation of complex $C3(H_2O)CR1$	$k^{-}_{\mathrm{C3(H_2O)CR1}}$	1.0×10^{-2} s^{-1}	$1.0 \times 10^{-3} - 1.0 \times 10^{-1}$ s^{-1}
Association of DAF to C3 convertase on host cell	$k^{+}_{\mathrm{C3bBbDAF}}$	2.0×10^{3} $M^{-1}s^{-1}$	$2.0 \times 10^{2} - 2.0 \times 10^{4}$ $M^{-1}s^{-1}$
Decay of C3 convertase by inhibitor DAF on host cell	$k^{-}_{\mathrm{C3bBbDAF}}$	7.7×10^{-2} s^{-1}	$7.7 \times 10^{-3} - 7.7 \times 10^{-1}$ s^{-1}
Decay of C3 convertase by inhibitor CR1 on host cell	$k^{-}_{\mathrm{C3bBbCR1}}$	7.7×10^{-2} s^{-1}	$7.7 \times 10^{-3} - 7.7 \times 10^{-1}$ s^{-1}
Decay of C3 convertase by inhibitor Factor H on host cell	k^{-}_{C3bBbH}	7.7×10^{-2} s^{-1}	$7.7 \times 10^{-3} - 7.7 \times 10^{-1}$ s^{-1}

Association of iC3b to CR1	k^{+}_{iC3bCR1}	2.0×10^{3} $M^{-1}s^{-1}$	$2.0\times10^{2} - 2.0\times10^{4}$ $M^{-1}s^{-1}$
Dissociation of complex iC3bCR1	k^{-}_{iC3bCR1}	1.0×10^{-2} s^{-1}	$1.0\times10^{-3} - 1.0\times10^{-1}$ s^{-1}
Association of C3b to C3bBb	$k^{+}_{\mathrm{C3bBbC3b}}$	3.5×10^{6} $M^{-1}s^{-1}$	$3.5\times10^{5} - 3.5\times10^{7}$ $M^{-1}s^{-1}$
Dissociation of complex C3bBbC3b	$k^{-}_{\mathrm{C3bBbC3b}}$	3.8×10^{-3} s^{-1}	$3.8\times10^{-4} - 3.8\times10^{-2}$ s^{-1}
Association of C5 to C3bBbC3b	$k^{+}_{\mathrm{C3bBbC3bC5}}$	5.0×10^{6} $M^{-1}s^{-1}$	$5.0\times10^{5} - 5.0\times10^{7}$ $M^{-1}s^{-1}$
Dissociation of complex C3bBbC3bC5	$k^{-}_{\mathrm{C3bBbC3bC5}}$	1.0×10^{-2} s^{-1}	$1.0\times10^{-3} - 1.0\times10^{-1}$ s^{-1}
Dissociation of complex C3bBbC3bC5b	k^{-}_{C5b}	3.8×10^{-2} s^{-1}	$3.8\times10^{-3} - 3.8\times10^{-1}$ s^{-1}
Association of C6 to C3bBbC3bC5b	$k^{+}_{\mathrm{C3bBbC3bC5C}}$	6.0×10^{4} $M^{-1}s^{-1}$	$6.0\times10^{3} - 6.0\times10^{5}$ $M^{-1}s^{-1}$
Dissociation of complex C3bBbC3bC5bC6	$k^{-}_{\mathrm{C3bBbC3bC5b}}$	9×10^{-8} s^{-1}	$9\times10^{-9} - 9\times10^{-7}$ s^{-1}
Association of C7 to C3bBbC3bC5bC6	k^{+}_{C5b7}	7.3×10^{5} $M^{-1}s^{-1}$	$7.3\times10^{4} - 7.3\times10^{6}$ $M^{-1}s^{-1}$
Dissociation of complex C3bBbC3bC5bC6C7	k^{-}_{C5b7}	1.5×10^{-6} s^{-1}	$1.5\times10^{-7} - 1.5\times10^{-5}$ s^{-1}
Attachment of C5b7 to host cell and pathogen	$k^{+}_{\mathrm{C5b7}_{\mathrm{surface}}}$	4.2×10^{8} $M^{-1}s^{-1}$	$4.2\times10^{7} - 4.2\times10^{9}$ $M^{-1}s^{-1}$

Formation of C5b7 micelle in fluid	k^{+}_{micelle}	69.3 s^{-1}	6.93 – 693 s^{-1}
Association of C8 to C5b7	k^{+}_{C5b8}	1.1×10^{6} $M^{-1}s^{-1}$	$1.1\times10^{5} - 1.1\times10^{7}$ $M^{-1}s^{-1}$
Dissociation of complex C5b8	k^{-}_{C5b8}	9.8×10^{-7} s^{-1}	$9.8\times10^{-8} - 9.8\times10^{-6}$ s^{-1}
Association of C9 to C5b8	k^{+}_{C5b9}	2.8×10^{6} $M^{-1}s^{-1}$	$2.8\times10^{5} - 2.8\times10^{7}$ $M^{-1}s^{-1}$
Dissociation of complex C5b9	k^{-}_{C5b9}	1.4×10^{-7} s^{-1}	$1.4\times10^{-8} - 1.4\times10^{-6}$ s^{-1}
Association of Cn to C5b7	k^{+}_{CnC5b7}	4.1×10^{5} $M^{-1}s^{-1}$	$4.1\times10^{4} - 4.1\times10^{6}$ $M^{-1}s^{-1}$
Dissociation of complex CnC5b7	k^{-}_{CnC5b7}	4.0×10^{-3} s^{-1}	$4.0\times10^{-4} - 4.0\times10^{-2}$ s^{-2}
Association of Cn to C5b8	k^{+}_{CnC5b8}	4.1×10^{5} $M^{-1}s^{-1}$	$4.1\times10^{4} - 4.1\times10^{6}$ $M^{-1}s^{-1}$
Dissociation of complex CnC5b8	k^{-}_{CnC5b8}	4.0×10^{-3} s^{-1}	$4.0\times10^{-4} - 4.0\times10^{-2}$ s^{-1}
Association of Vn to C5b7	k^{+}_{VnC5b7}	2.4×10^{5} $M^{-1}s^{-1}$	$2.4\times10^{4} - 2.4\times10^{6}$ $M^{-1}s^{-1}$
Dissociation of complex VnC5b7	k^{-}_{VnC5b7}	2.0×10^{-3} s^{-1}	$2.0\times10^{-4} - 2.0\times10^{-2}$ s^{-1}
Association of CD59 to C5b9	k^{+}_{CD59C5b9}	1.0×10^{6} $M^{-1}s^{-1}$	$1.0\times10^{5} - 1.0\times10^{7}$ $M^{-1}s^{-1}$
Dissociation of complex CD59C5b9	k^{-}_{CD59C5b9}	2.0×10^{-4} s^{-1}	$2.0\times10^{-5} - 2.0\times10^{-3}$ s^{-1}

Cleavage of C3 by C3 convertase, $C3(H_20)Bb$	k_{cat} $C3(H_2O)Bb$<br>K_m $C3(H_2O)Bb$	1.8 s^{-1}<br>5.9×10^{-6} M	$0.18 - 18$ s^{-1}<br>$5.9\times10^{-5} - 5.9\times10^{-4}$ M
Cleavage of C3 by C3 convertase, C3bBb	k_{cat} C3bBb<br>K_m C3bBb	1.8 s^{-1}<br>5.9×10^{-6} M	$0.18 - 18$ s^{-1}<br>$5.9\times10^{-5} - 5.9\times10^{-4}$ M
Cleavage of C3 by C3 convertase, C3bBbP	k_{cat} C3bBbP<br>K_m C3bBbP	3.1 s^{-1}<br>1.8×10^{-6} M	$0.18 - 18$ s^{-1}<br>$1.8\times10^{-5} - 1.8\times10^{-4}$ M
Activation of complex C3bB by enzyme Factor D	k_{cat} C3bB<br>K_m C3bB	2.1 s^{-1}<br>0.1×10^{-6} M	$0.21 - 21$ s^{-1}<br>$0.1\times10^{-5} - 0.1\times10^{-4}$ M
Activation of complex $C3(H_2O)B$ by enzyme Factor D	k_{cat} $C3(H_2O)B$<br>K_m $C3(H_2O)B$	2.1 s^{-1}<br>0.1×10^{-6} M	$0.21 - 21$ s^{-1}<br>$0.1\times10^{-5} - 0.1\times10^{-4}$ M
Cleavage of C3b by inhibitor Factor I	k_{cat} C3bH<br>K_m C3bH	1.3 s^{-1}<br>2.5×10^{-7} M	$0.13 - 13$ s^{-1}<br>$2.5\times10^{-8} - 2.5\times10^{-6}$ M
Cleavage of C5 by the C5 convertase, C3bBbC3b and C3bBbC3bP	k_{cat} C3bBbC3b<br>K_m C3bBbC3b	4.8 s^{-1}<br>1.8×10^{-6} M	$0.48 - 48$ s^{-1}<br>$1.8\times10^{-7} - 1.8\times10^{-5}$ M
