# Supplementary material for: Quantitative Modeling of the Alternative Pathway of the Complement System
Source: PLoS One. 2016 Mar 31;11(3):e0152337. doi: 10.1371/journal.pone.0152337 (PMC4816337; doi:10.1371/journal.pone.0152337)
Supplement: S1 Text — System of 107 Ordinary Differential Equations. (PDF) [file pone.0152337.s011.pdf]

S1 Text. Mathematical Model. System of 107 Ordinary Differential Equations.

(i) Initiation (fluid phase)

1

$$
\begin{aligned}
\frac{d[\text{C3(H}_2\text{O)}_{\text{fluid}}]}{dt} &= k^{+}_{\text{C3(H}_2\text{O)}}[\text{C3}] - k^{+}_{\text{C3(H}_2\text{O)B}}[\text{C3(H}_2\text{O)}_{\text{fluid}}][\text{B}] + k^{-}_{\text{C3(H}_2\text{O)B}}[\text{C3(H}_2\text{O)B}_{\text{fluid}}] - k^{+}_{\text{C3bH}}[\text{C3(H}_2\text{O)}_{\text{fluid}}][\text{H}] \\
&+ k^{-}_{\text{C3bH}}[\text{C3(H}_2\text{O)H}_{\text{fluid}}] - k^{+}_{\text{C3bCR1}}[\text{C3(H}_2\text{O)}_{\text{fluid}}][\text{CR1}] + k^{-}_{\text{C3bCR1}}[\text{C3(H}_2\text{O)CR1}_{\text{fluid}}] + k^{-}_{\text{C3(H}_2\text{O)Bb}}[\text{C3(H}_2\text{O)Bb}_{\text{fluid}}] \\
&+ k^{-}_{\text{C3bBbH}_{\text{decay}}}[\text{C3(H}_2\text{O)BbH}_{\text{fluid}}]
\end{aligned}
$$

2

$$
\begin{aligned}
\frac{d[\text{C3(H}_2\text{O)B}_{\text{fluid}}]}{dt} &= k^{+}_{\text{C3(H}_2\text{O)B}}[\text{C3(H}_2\text{O)}_{\text{fluid}}][\text{B}] - k^{-}_{\text{C3(H}_2\text{O)B}}[\text{C3(H}_2\text{O)B}_{\text{fluid}}] \\
&- \frac{k^{\text{D}}_{\text{catC3(H}_2\text{O)B}}[\text{D}][\text{C3(H}_2\text{O)B}_{\text{fluid}}]}{K^{\text{D}}_{\text{mC3(H}_2\text{O)B}} + [\text{C3bB}_{\text{fluid}}] + [\text{C3(H}_2\text{O)B}_{\text{fluid}}] + [\text{C3bB}_{\text{host}}] + [\text{C3bB}_{\text{pathogen}}] + [\text{C3bBP}_{\text{pathogen}}] + [\text{C3bBP*}_{\text{pathogen}}]}
\end{aligned}
$$

3

$$
\begin{aligned}
\frac{d[\text{C3(H}_2\text{O)Bb}_{\text{fluid}}]}{dt} &= -k^{-}_{\text{C3(H}_2\text{O)Bb}}[\text{C3(H}_2\text{O)Bb}_{\text{fluid}}] - k^{+}_{\text{C3bH}}[\text{C3(H}_2\text{O)Bb}_{\text{fluid}}][\text{H}] \\
&+ \frac{k^{\text{D}}_{\text{catC3(H}_2\text{O)B}}[\text{D}][\text{C3(H}_2\text{O)B}_{\text{fluid}}]}{K^{\text{D}}_{\text{mC3(H}_2\text{O)B}} + [\text{C3bB}_{\text{fluid}}] + [\text{C3(H}_2\text{O)B}_{\text{fluid}}] + [\text{C3bB}_{\text{host}}] + [\text{C3bB}_{\text{pathogen}}] + [\text{C3bBP}_{\text{pathogen}}] + [\text{C3bBP*}_{\text{pathogen}}]}
\end{aligned}
$$

4

$$
\begin{aligned}
\frac{d[\text{fC3b}]}{dt} &= k^{+}_{\text{fC3b}}[\text{nfC3b}][\text{H}_2\text{O}] + k^{+}_{\text{fC3b}}[\text{npC3b}][\text{H}_2\text{O}] + k^{+}_{\text{fC3b}}[\text{nhC3b}][\text{H}_2\text{O}] - k^{+}_{\text{C3bB}}[\text{C3b}_{\text{fluid}}][\text{B}] + k^{-}_{\text{C3bB}}[\text{C3bB}_{\text{fluid}}] - k^{+}_{\text{C3bH}}[\text{C3b}_{\text{fluid}}][\text{H}] \\
&+ k^{-}_{\text{C3bH}}[\text{C3bH}_{\text{fluid}}] - k^{+}_{\text{C3bP}}[\text{P*}_{\text{pathogen}}][\text{fC3b}] + k^{-}_{\text{C3bP}}[\text{C3bP*}_{\text{surface}}] - k^{+}_{\text{C3bCR1}}[\text{C3b}_{\text{fluid}}][\text{CR1}] + k^{-}_{\text{C3bCR1}}[\text{C3bCR1}_{\text{fluid}}] + k^{-}_{\text{C3bBb}}[\text{C3bBb}_{\text{fluid}}] \\
&+ k^{-}_{\text{C3bBbH}_{\text{decay}}}[\text{C3bBbH}_{\text{fluid}}] + k^{-}_{\text{C3bBbCR1}_{\text{decay}}}[\text{C3bBbCR1}_{\text{fluid}}] - k^{+}_{\text{C3bBbC3b}}[\text{C3bBb}_{\text{host}}][\text{fC3b}] - k^{+}_{\text{C3bBbC3b}}[\text{C3bBb}_{\text{pathogen}}][\text{fC3b}] \\
&- k^{+}_{\text{C3bBbC3b}}[\text{C3bBbP}_{\text{pathogen}}][\text{fC3b}] - k^{+}_{\text{C3bBbC3b}}[\text{C3bBbP*}_{\text{pathogen}}][\text{fC3b}] + k^{-}_{\text{C3bBbC3b}}[\text{C3bBbC3b}_{\text{host}}] + k^{-}_{\text{C3bBbC3b}}[\text{C3bBbC3b}_{\text{pathogen}}] \\
&+ k^{-}_{\text{C3bBbCR1}_{\text{decay}}}[\text{C3bBbC3bCR1}_{\text{host}}] + k^{-}_{\text{C3bBbDAF}_{\text{decay}}}[\text{C3bBbC3bDAF}_{\text{host}}] + k^{-}_{\text{C3bBbH}_{\text{decay}}}[\text{C3bBbC3bH}_{\text{host}}] + k^{-}_{\text{P*}_{\text{surface}}}[\text{C3bP*}_{\text{pathogen}}] \\
&+ k^{-}_{\text{P*}_{\text{surface}}}[\text{C3bBP*}_{\text{pathogen}}] + k^{-}_{\text{P*}_{\text{surface}}}[\text{C3bBbP*}_{\text{pathogen}}] + k^{-}_{\text{P*}_{\text{surface}}}[\text{C3bBbC3bP*}_{\text{pathogen}}] + k^{-}_{\text{P*}_{\text{surface}}}[\text{C3bBbC3bC5P*}_{\text{pathogen}}] \\
&+ k^{-}_{\text{P*}_{\text{surface}}}[\text{C3bBbC3bC5bP*}_{\text{pathogen}}] + k^{-}_{\text{P*}_{\text{surface}}}[\text{C3bBbC3bC5bPC6*}_{\text{pathogen}}]
\end{aligned}
$$

5

$$
\begin{aligned}
\frac{d[\text{C3bB}_{\text{fluid}}]}{dt} &= k^{+}_{\text{C3bB}}[\text{C3b}_{\text{fluid}}][\text{B}] - k^{-}_{\text{C3bB}}[\text{C3bB}_{\text{fluid}}] \\
&- \frac{k^{\text{D}}_{\text{catC3bB}}[\text{D}][\text{C3bB}_{\text{fluid}}]}{K^{\text{D}}_{\text{mC3(H}_2\text{O)B}} + [\text{C3bB}_{\text{fluid}}] + [\text{C3(H}_2\text{O)B}_{\text{fluid}}] + [\text{C3bB}_{\text{host}}] + [\text{C3bB}_{\text{pathogen}}] + [\text{C3bBP}_{\text{pathogen}}] + [\text{C3bBP*}_{\text{pathogen}}]}
\end{aligned}
$$

6

$$\frac{d[\mathrm{C3bBb_{fluid}}]}{dt} = -k^{-}_{\mathrm{C3bBb}}[\mathrm{C3bBb_{fluid}}] - k^{+}_{\mathrm{C3bH}}[\mathrm{C3bBb_{fluid}}][\mathrm{H}] - k^{+}_{\mathrm{C3bCR1}}[\mathrm{C3bBb_{fluid}}][\mathrm{CR1}]$$

$$+ \frac{k^{\mathrm{D}}_{\mathrm{catC3bB}}[\mathrm{D}][\mathrm{C3bB_{fluid}}]}{K^{\mathrm{D}}_{\mathrm{mC3(H_2O)B}} + [\mathrm{C3bB_{fluid}}] + [\mathrm{C3(H_2O)B_{fluid}}] + [\mathrm{C3bB_{host}}] + [\mathrm{C3bB_{pathogen}}] + [\mathrm{C3bBP_{pathogen}}] + [\mathrm{C3bBP*_{pathogen}}]}$$

7

$$\frac{d[\mathrm{nfC3b}]}{dt} = \frac{k^{\mathrm{C3}}_{\mathrm{catC3(H_2O)Bb}}[\mathrm{C3}][\mathrm{C3(H_2O)Bb_{fluid}}]}{K^{\mathrm{C3}}_{\mathrm{mC3(H_2O)Bb}} + [\mathrm{C3}]} + \frac{k^{\mathrm{C3}}_{\mathrm{catC3bBb}}[\mathrm{C3}][\mathrm{C3bBb_{fluid}}]}{K^{\mathrm{C3}}_{\mathrm{mC3bBb}} + [\mathrm{C3}]} - k^{+}_{\mathrm{fC3b}}[\mathrm{nfC3b}][\mathrm{H_2O}] - k^{+}_{\mathrm{C3bP}}[\mathrm{P*_{surface}}][\mathrm{nfC3b}]$$

$$- k^{+}_{\mathrm{C3b_{surface}}}[\mathrm{nfC3b}][\mathrm{Surface_{host}}] - k^{+}_{\mathrm{C3b_{surface}}}[\mathrm{nfC3b}][\mathrm{Surface_{pathogen}}]$$

8

$$\frac{d[\mathrm{H_2O}]}{dt} = -k^{+}_{\mathrm{fC3b}}[\mathrm{nfC3b}][\mathrm{H_2O}] - k^{+}_{\mathrm{fC3b}}[\mathrm{npC3b}][\mathrm{H_2O}] - k^{+}_{\mathrm{fC3b}}[\mathrm{nhC3b}][\mathrm{H_2O}]$$

(ii) Amplification (pathogen)

1

$$\frac{d[\mathrm{P*_{neutrophil}}]}{dt} = k^{+}_{\mathrm{P*\ released}}[\mathrm{P*_{neutrophil}}]$$

2

$$\frac{d[\mathrm{Surface_{pathogen}}]}{dt} = -k^{+}_{\mathrm{C3b_{surface}}}[\mathrm{nfC3b}][\mathrm{Surface_{pathogen}}] - k^{+}_{\mathrm{pC3b}}[\mathrm{npC3b}][\mathrm{Surface_{pathogen}}]\mathrm{x}(3.52\times 10^{10})$$

$$- k^{+}_{\mathrm{P*_{surface}}}[\mathrm{P*}][\mathrm{Surface_{pathogen}}] - k^{+}_{\mathrm{C5b7_{surface}}}[\mathrm{pC5b7_{fluid}}]\times 4\times(3.10\times 10^{9})$$

3

$$\frac{d[\mathrm{C3b_{pathogen}}]}{dt} = k^{+}_{\mathrm{C3b_{surface}}}[\mathrm{nfC3b}][\mathrm{Surface_{pathogen}}] + k^{+}_{\mathrm{pC3b}}[\mathrm{npC3b}][\mathrm{Surface_{pathogen}}]\mathrm{x}(3.52*10^{10}) - k^{+}_{\mathrm{C3bB}}[\mathrm{C3b_{pathogen}}][\mathrm{B}]$$

$$+ k^{-}_{\mathrm{C3bB}}[\mathrm{C3bB_{pathogen}}] - k^{+}_{\mathrm{C3bP}}[\mathrm{C3b_{pathogen}}][\mathrm{P*}] + k^{-}_{\mathrm{C3bP}}[\mathrm{C3bP*_{pathogen}}] + k^{-}_{\mathrm{C3bBb}}[\mathrm{C3bBb_{pathogen}}] + k^{-}_{\mathrm{C3bBbP}}[\mathrm{C3bBbP_{pathogen}}]$$

$$- k^{+}_{\mathrm{C3bCR1}}[\mathrm{C3b_{pathogen}}][\mathrm{CR1}] + k^{-}_{\mathrm{C3bCR1}}[\mathrm{C3bCR1_{pathogen}}]$$

4

$$\frac{d[\mathrm{P^*}_{\mathrm{pathogen}}]}{dt} = k^{+}_{\mathrm{P^*}_{\mathrm{surface}}}[\mathrm{P^*}][\mathrm{Surface}_{\mathrm{pathogen}}] - k^{-}_{\mathrm{P^*}_{\mathrm{surface}}}[\mathrm{PSurface^*}_{\mathrm{pathogen}}] - k^{+}_{\mathrm{C3bP}}[\mathrm{fC3b}][\mathrm{P^*}_{\mathrm{surface}}] - k^{+}_{\mathrm{C3bP}}[\mathrm{nfC3b}][\mathrm{P^*}_{\mathrm{surface}}]$$
$$- k^{+}_{\mathrm{C3bP}}[\mathrm{npC3b}][\mathrm{P^*}_{\mathrm{surface}}] + k^{-}_{\mathrm{C3bP}}[\mathrm{C3bP^*}_{\mathrm{surface}}] + k^{-}_{\mathrm{C3bP}}[\mathrm{C3bBP^*}_{\mathrm{pathogen}}] + k^{-}_{\mathrm{C3bP}}[\mathrm{C3bBbP^*}_{\mathrm{pathogen}}] + k^{-}_{\mathrm{C3bP}}[\mathrm{C3bBbC3bP^*}_{\mathrm{pathogen}}]$$
$$+ k^{-}_{\mathrm{C3bP}}[\mathrm{C3bBbC3bC5P^*}_{\mathrm{pathogen}}] + k^{-}_{\mathrm{C3bP}}[\mathrm{C3bBbC3bC5bP^*}_{\mathrm{pathogen}}] + k^{-}_{\mathrm{C3bP}}[\mathrm{C3bBbC3bC5bC6P^*}_{\mathrm{pathogen}}]$$

5

$$\frac{d[\mathrm{npC3b}]}{dt} = \frac{k^{\mathrm{C3}}_{\mathrm{catC3bBb}}[\mathrm{C3}][\mathrm{C3bBb}_{\mathrm{pathogen}}]}{K^{\mathrm{C3}}_{\mathrm{mC3bBb}} + [\mathrm{C3}]} + \frac{k^{\mathrm{C3}}_{\mathrm{catC3bBbP}}[\mathrm{C3}][\mathrm{C3bBbP}_{\mathrm{pathogen}}]}{K^{\mathrm{C3}}_{\mathrm{mC3bBbP}} + [\mathrm{C3}]} - k^{+}_{\mathrm{fC3b}}[\mathrm{npC3b}][\mathrm{H_2O}]$$
$$- k^{+}_{\mathrm{pC3b}}[\mathrm{npC3b}][\mathrm{Surface}_{\mathrm{pathogen}}] \times (3.52 \times 10^{10}) - k^{+}_{\mathrm{C3bP}}[\mathrm{npC3b}][\mathrm{P^*}_{\mathrm{pathogen}}] + k^{-}_{\mathrm{C3bP}}[\mathrm{npC3bP^*}_{\mathrm{pathogen}}]$$
$$- k^{+}_{\mathrm{C3bBbC3b}}[\mathrm{C3bBb}_{\mathrm{pathogen}}][\mathrm{npC3b}] - k^{+}_{\mathrm{C3bBbC3b}}[\mathrm{C3bBbP}_{\mathrm{pathogen}}][\mathrm{npC3b}] - k^{+}_{\mathrm{C3bBb}}[\mathrm{C3bBbP^*}_{\mathrm{pathogen}}][\mathrm{npC3b}]$$

6

$$\frac{d[\mathrm{C3bB}_{\mathrm{pathogen}}]}{dt} = k^{+}_{\mathrm{C3bB}}[\mathrm{C3b}_{\mathrm{pathogen}}][\mathrm{B}] - k^{-}_{\mathrm{C3bB}}[\mathrm{C3bB}_{\mathrm{pathogen}}] - k^{+}_{\mathrm{C3bP}}[\mathrm{C3bB}_{\mathrm{pathogen}}][\mathrm{P^*}] + k^{-}_{\mathrm{C3bP}}[\mathrm{C3bBP^*}_{\mathrm{pathogen}}]$$
$$- \frac{k^{\mathrm{D}}_{\mathrm{catC3bB}}[\mathrm{D}][\mathrm{C3bB}_{\mathrm{pathogen}}]}{K^{\mathrm{D}}_{\mathrm{mC3(H_2O)B}} + [\mathrm{C3bB}_{\mathrm{fluid}}] + [\mathrm{C3(H_2O)B}_{\mathrm{fluid}}] + [\mathrm{C3bB}_{\mathrm{host}}] + [\mathrm{C3bB}_{\mathrm{pathogen}}] + [\mathrm{C3bBP}_{\mathrm{pathogen}}] + [\mathrm{C3bBP^*}_{\mathrm{pathogen}}]}$$

7

$$\frac{d[\mathrm{C3bBb}_{\mathrm{pathogen}}]}{dt} = -k^{+}_{\mathrm{C3bP}}[\mathrm{C3bBb}_{\mathrm{pathogen}}][\mathrm{P}] - k^{+}_{\mathrm{C3bP}}[\mathrm{C3bBb}_{\mathrm{pathogen}}][\mathrm{P^*}] + k^{-}_{\mathrm{C3bBb}}[\mathrm{C3bBb}_{\mathrm{pathogen}}] - k^{+}_{\mathrm{C3bBbC3b}}[\mathrm{C3bBb}_{\mathrm{pathogen}}][\mathrm{fC3b}]$$
$$- k^{+}_{\mathrm{C3bBbC3b}}[\mathrm{C3bBb}_{\mathrm{pathogen}}][\mathrm{npC3b}] + k^{-}_{\mathrm{C3bBbC3b}}[\mathrm{C3bBbC3b}_{\mathrm{pathogen}}]$$
$$+ \frac{k^{\mathrm{D}}_{\mathrm{catC3bB}}[\mathrm{D}][\mathrm{C3bB}_{\mathrm{pathogen}}]}{K^{\mathrm{D}}_{\mathrm{mC3(H_2O)B}} + [\mathrm{C3bB}_{\mathrm{fluid}}] + [\mathrm{C3(H_2O)B}_{\mathrm{fluid}}] + [\mathrm{C3bB}_{\mathrm{host}}] + [\mathrm{C3bB}_{\mathrm{pathogen}}] + [\mathrm{C3bBP}_{\mathrm{pathogen}}] + [\mathrm{C3bBP^*}_{\mathrm{pathogen}}]}$$

8

$$\frac{d[\mathrm{C3bP}_{\mathrm{pathogen}}]}{dt} = k^{+}_{\mathrm{C3bP}}[\mathrm{C3b}_{\mathrm{pathogen}}][\mathrm{P^*}] - k^{-}_{\mathrm{C3bP}}[\mathrm{C3bP^*}_{\mathrm{pathogen}}] - k^{+}_{\mathrm{C3bBP}}[\mathrm{C3bP^*}_{\mathrm{pathogen}}][\mathrm{B}] + k^{-}_{\mathrm{C3bP}}[\mathrm{C3bBP^*}_{\mathrm{pathogen}}]$$

9

$$\frac{d[\mathrm{C3bBP}_{\mathrm{pathogen}}]}{dt} = k^{+}_{\mathrm{C3bBP}}[\mathrm{C3bP^*}_{\mathrm{pathogen}}][\mathrm{B}] + k^{+}_{\mathrm{C3bP}}[\mathrm{C3bB}_{\mathrm{pathogen}}][\mathrm{P^*}] - k^{-}_{\mathrm{C3bP}}[\mathrm{C3bBP^*}_{\mathrm{pathogen}}]$$
$$- \frac{k^{\mathrm{D}}_{\mathrm{catC3bB}}[\mathrm{D}][\mathrm{C3bBP}_{\mathrm{pathogen}}]}{K^{\mathrm{D}}_{\mathrm{mC3(H_2O)B}} + [\mathrm{C3bB}_{\mathrm{fluid}}] + [\mathrm{C3(H_2O)B}_{\mathrm{fluid}}] + [\mathrm{C3bB}_{\mathrm{host}}] + [\mathrm{C3bB}_{\mathrm{pathogen}}] + [\mathrm{C3bBP}_{\mathrm{pathogen}}] + [\mathrm{C3bBP^*}_{\mathrm{pathogen}}]}$$

10

$$\frac{d[\mathrm{C3bBbP}_{\mathrm{pathogen}}]}{dt} = k^{+}_{\mathrm{C3bP}}[\mathrm{C3bBb}_{\mathrm{pathogen}}][\mathrm{P}] + k^{+}_{\mathrm{C3bP}}[\mathrm{C3bBb}_{\mathrm{pathogen}}][\mathrm{P}^{*}] - k^{-}_{\mathrm{C3bBbP}}[\mathrm{C3bBbP}_{\mathrm{pathogen}}]$$

$$- k^{+}_{\mathrm{C3bBbC3b}}[\mathrm{C3bBbP}_{\mathrm{pathogen}}][\mathrm{fC3b}] - k^{+}_{\mathrm{C3bBbC3b}}[\mathrm{C3bBbP}_{\mathrm{pathogen}}][\mathrm{npC3b}]$$

$$+ \frac{k^{\mathrm{D}}_{\mathrm{catC3bB}}[\mathrm{D}][\mathrm{C3bBP}_{\mathrm{pathogen}}]}{K^{\mathrm{D}}_{\mathrm{mC3(H_2O)B}} + [\mathrm{C3bB}_{\mathrm{fluid}}] + [\mathrm{C3(H_2O)B}_{\mathrm{fluid}}] + [\mathrm{C3bB}_{\mathrm{host}}] + [\mathrm{C3bB}_{\mathrm{pathogen}}] + [\mathrm{C3bBP}_{\mathrm{pathogen}}] + [\mathrm{C3bBP}^{*}_{\mathrm{pathogen}}]}$$

11

$$\frac{d[\mathrm{C3bP}^{*}_{\mathrm{pathogen}}]}{dt} = k^{+}_{\mathrm{C3bP}}[\mathrm{P}^{*}_{\mathrm{surface}}][\mathrm{fC3b}] + k^{+}_{\mathrm{C3bP}}[\mathrm{P}^{*}_{\mathrm{surface}}][\mathrm{npC3b}] + k^{+}_{\mathrm{C3bP}}[\mathrm{P}^{*}_{\mathrm{surface}}][\mathrm{nfC3b}] - k^{+}_{\mathrm{C3bBP}}[\mathrm{C3bP}^{*}_{\mathrm{pathogen}}][\mathrm{B}]$$

$$- k^{-}_{\mathrm{C3bP}}[\mathrm{C3bP}^{*}_{\mathrm{pathogen}}] - k^{-}_{\mathrm{P}^{*}_{\mathrm{surface}}}[\mathrm{C3bP}^{*}_{\mathrm{pathogen}}]$$

12

$$\frac{d[\mathrm{C3bBP}^{*}_{\mathrm{pathogen}}]}{dt} = k^{+}_{\mathrm{C3bBP}}[\mathrm{C3bP}^{*}_{\mathrm{pathogen}}][\mathrm{B}] - k^{-}_{\mathrm{C3bP}}[\mathrm{C3bBP}^{*}_{\mathrm{pathogen}}] - k^{-}_{\mathrm{P}^{*}_{\mathrm{surface}}}[\mathrm{C3bBP}^{*}_{\mathrm{pathogen}}]$$

$$- \frac{k^{\mathrm{D}}_{\mathrm{catC3bB}}[\mathrm{D}][\mathrm{C3bBP}_{\mathrm{pathogen}}]}{K^{\mathrm{D}}_{\mathrm{mC3(H_2O)B}} + [\mathrm{C3bB}_{\mathrm{fluid}}] + [\mathrm{C3(H_2O)B}_{\mathrm{fluid}}] + [\mathrm{C3bB}_{\mathrm{host}}] + [\mathrm{C3bB}_{\mathrm{pathogen}}] + [\mathrm{C3bBP}_{\mathrm{pathogen}}] + [\mathrm{C3bBP}^{*}_{\mathrm{pathogen}}]}$$

13

$$\frac{d[\mathrm{C3bBbP}^{*}_{\mathrm{pathogen}}]}{dt} = - k^{-}_{\mathrm{C3bBbP}}[\mathrm{C3bBbP}^{*}_{\mathrm{pathogen}}] - k^{+}_{\mathrm{C3bBbC3b}}[\mathrm{C3bBbP}^{*}_{\mathrm{pathogen}}][\mathrm{fC3b}] - k^{+}_{\mathrm{C3bBbC3b}}[\mathrm{C3bBbP}^{*}_{\mathrm{pathogen}}][\mathrm{npC3b}]$$

$$- k^{-}_{\mathrm{P}^{*}_{\mathrm{surface}}}[\mathrm{C3bBbP}^{*}_{\mathrm{pathogen}}]$$

$$+ \frac{k^{\mathrm{D}}_{\mathrm{catC3bB}}[\mathrm{D}][\mathrm{C3bBP}^{*}_{\mathrm{pathogen}}]}{K^{\mathrm{D}}_{\mathrm{mC3(H_2O)B}} + [\mathrm{C3bB}_{\mathrm{fluid}}] + [\mathrm{C3(H_2O)B}_{\mathrm{fluid}}] + [\mathrm{C3bB}_{\mathrm{host}}] + [\mathrm{C3bB}_{\mathrm{pathogen}}] + [\mathrm{C3bBP}_{\mathrm{pathogen}}] + [\mathrm{C3bBP}^{*}_{\mathrm{pathogen}}]}$$

14

$$\frac{d[\mathrm{C3bCR1}_{\mathrm{pathogen}}]}{dt} = k^{+}_{\mathrm{C3bCR1}}[\mathrm{C3b}_{\mathrm{pathogen}}][\mathrm{CR1}] - k^{-}_{\mathrm{C3bCR1}}[\mathrm{C3bCR1}_{\mathrm{pathogen}}]$$

$$- \frac{k^{\mathrm{FI}}_{\mathrm{catC3bH}}[\mathrm{I}][\mathrm{C3bCR1}_{\mathrm{pathogen}}]}{K^{\mathrm{FI}}_{\mathrm{mC3bH}} + [\mathrm{C3bH}_{\mathrm{fluid}}] + [\mathrm{C3bCR1}_{\mathrm{fluid}}] + [\mathrm{iC3bCR1}_{\mathrm{fluid}}] + [\mathrm{C3bH}_{\mathrm{host}}] + [\mathrm{C3bCR1}_{\mathrm{host}}] + [\mathrm{iC3bCR1}_{\mathrm{host}}] + [\mathrm{iC3bCR1}_{\mathrm{pathogen}}] + [\mathrm{C3bCR1}_{\mathrm{pathogen}}]}$$

15

$$\frac{d[\mathrm{iC3b}_{\mathrm{pathogen}}]}{dt} = - k^{+}_{\mathrm{iC3bCR1}}[\mathrm{iC3b}_{\mathrm{pathogen}}][\mathrm{CR1}] + k^{-}_{\mathrm{iC3bCR1}}[\mathrm{iC3bCR1}_{\mathrm{pathogen}}] - k^{+}_{\mathrm{C3bP}}[\mathrm{iC3b}_{\mathrm{pathogen}}][\mathrm{P}] + k^{-}_{\mathrm{iC3bP}}[\mathrm{iC3bP}_{\mathrm{pathogen}}]$$

$$+ \frac{k^{\mathrm{FI}}_{\mathrm{catC3bH}}[\mathrm{I}][\mathrm{C3bCR1}_{\mathrm{pathogen}}]}{K^{\mathrm{FI}}_{\mathrm{mC3bH}} + [\mathrm{C3bH}_{\mathrm{fluid}}] + [\mathrm{C3bCR1}_{\mathrm{fluid}}] + [\mathrm{iC3bCR1}_{\mathrm{fluid}}] + [\mathrm{C3bH}_{\mathrm{host}}] + [\mathrm{C3bCR1}_{\mathrm{host}}] + [\mathrm{iC3bCR1}_{\mathrm{host}}] + [\mathrm{iC3bCR1}_{\mathrm{pathogen}}] + [\mathrm{C3bCR1}_{\mathrm{pathogen}}]}$$

16

$$\frac{d[\text{iC3bCR1}_{\text{pathogen}}]}{dt} = k^{+}_{\text{iC3bCR1}}[\text{iC3b}_{\text{pathogen}}][\text{CR1}] - k^{-}_{\text{iC3bCR1}}[\text{iC3bCR1}_{\text{pathogen}}]$$

$$- \frac{k^{\text{FI}}_{\text{catC3bH}}[\text{I}][\text{iC3bCR1}_{\text{pathogen}}]}{K^{\text{FI}}_{\text{mC3bH}} + [\text{C3bH}_{\text{fluid}}] + [\text{C3bCR1}_{\text{fluid}}] + [\text{iC3bCR1}_{\text{fluid}}] + [\text{C3bH}_{\text{host}}] + [\text{C3bCR1}_{\text{host}}] + [\text{iC3bCR1}_{\text{host}}] + [\text{iC3bCR1}_{\text{pathogen}}] + [\text{C3bCR1}_{\text{pathogen}}]}$$

17

$$\frac{d[\text{C3dg}_{\text{pathogen}}]}{dt} =$$

$$\frac{k^{\text{FI}}_{\text{catC3bH}}[\text{I}][\text{iC3bCR1}_{\text{pathogen}}]}{K^{\text{FI}}_{\text{mC3bH}} + [\text{C3bH}_{\text{fluid}}] + [\text{C3bCR1}_{\text{fluid}}] + [\text{iC3bCR1}_{\text{fluid}}] + [\text{C3bH}_{\text{host}}] + [\text{C3bCR1}_{\text{host}}] + [\text{iC3bCR1}_{\text{host}}] + [\text{iC3bCR1}_{\text{pathogen}}] + [\text{C3bCR1}_{\text{pathogen}}]}$$

18

$$\frac{d[\text{iC3bP}_{\text{pathogen}}]}{dt} = k^{+}_{\text{iC3bP}}[\text{iC3b}_{\text{pathogen}}][\text{P}] - k^{-}_{\text{iC3bP}}[\text{iC3bP}_{\text{pathogen}}]$$

(iii) Termination (pathogen)

1

$$\frac{d[\text{C3bBbC3b}_{\text{pathogen}}]}{dt} = k^{+}_{\text{C3bBbC3b}}[\text{C3bBb}_{\text{pathogen}}][\text{npC3b}] + k^{+}_{\text{C3bBbC3b}}[\text{C3bBb}_{\text{pathogen}}][\text{fC3b}] + k^{-}_{\text{C3bBbC3b}}[\text{C3bBbC3b}_{\text{pathogen}}]$$

$$- k^{+}_{\text{C3bP}}[\text{C3bBbC3b}_{\text{pathogen}}][\text{P}] - k^{+}_{\text{C3bP}}[\text{C3bBbC3b}_{\text{pathogen}}][\text{P*}] - k^{+}_{\text{C3bBbC3bC5}}[\text{C3bBbC3b}_{\text{pathogen}}][\text{C5}] + k^{-}_{\text{C3bBbC3bC5}}[\text{C3bBbC3bC5}_{\text{pathogen}}]$$

$$+ k^{-}_{\text{C5b}}[\text{C3bBbC3bC5b}_{\text{pathogen}}] + k^{-}_{\text{C3bP}}[\text{C3bBbC3bP}_{\text{pathogen}}] + k^{+}_{\text{C5b7}}[\text{C3bBbC3bC5bC6}_{\text{pathogen}}][\text{C7}]$$

2

$$\frac{d[\text{C3bBbC3bC5}_{\text{pathogen}}]}{dt} = k^{+}_{\text{C3bBbC3bC5}}[\text{C3bBbC3b}_{\text{pathogen}}][\text{C5}] - k^{-}_{\text{C3bBbC3bC5}}[\text{C3bBbC3bC5}_{\text{pathogen}}] + k^{-}_{\text{C3bP}}[\text{C3bBbC3bPC5}_{\text{pathogen}}]$$

$$- k^{\text{kcat}}_{\text{C3bBbC3bC5}}[\text{C3bBbC3bC5}_{\text{pathogen}}]$$

3

$$\frac{d[\text{C3bBbC3bC5b}_{\text{pathogen}}]}{dt} = k^{\text{kcat}}_{\text{C3bBbC3bC5}}[\text{C3bBbC3bC5}_{\text{pathogen}}] - k^{-}_{\text{C5b}}[\text{C3bBbC3bC5b}_{\text{pathogen}}] + k^{-}_{\text{C3bP}}[\text{C3bBbC3bPC5b}_{\text{pathogen}}]$$

$$- k^{+}_{\text{C3bBbC3bC5bC6}}[\text{C3bBbC3bC5b}_{\text{pathogen}}][\text{C6}] + k^{-}_{\text{C3bBbC3bC5bC6}}[\text{C3bBbC3bC5bC6}_{\text{pathogen}}]$$

4

$$\frac{d[\mathrm{C3bBbC3bC5bC6}_{\mathrm{pathogen}}]}{dt} = k^{+}_{\mathrm{C3bBbC3bC5bC6}}[\mathrm{C3bBbC3bC5b}_{\mathrm{pathogen}}][\mathrm{C6}] - k^{-}_{\mathrm{C3bBbC3bC5bC6}}[\mathrm{C3bBbC3bC5bC6}_{\mathrm{pathogen}}]$$

$$+ k^{-}_{\mathrm{C3bP}}[\mathrm{C3bBbC3bPC5bC6}_{\mathrm{pathogen}}] - k^{+}_{\mathrm{C5b7}}[\mathrm{C3bBbC3bC5bC6}_{\mathrm{pathogen}}][\mathrm{C7}]$$

5

$$\frac{d[\mathrm{C3bBbC3bP}_{\mathrm{pathogen}}]}{dt} = k^{+}_{\mathrm{C3bBbC3b}}[\mathrm{C3bBbP}_{\mathrm{pathogen}}][\mathrm{fC3b}] + k^{+}_{\mathrm{C3bP}}[\mathrm{C3bBbC3b}_{\mathrm{pathogen}}][\mathrm{P}] + k^{+}_{\mathrm{C3bP}}[\mathrm{C3bBbC3b}_{\mathrm{pathogen}}][\mathrm{P*}]$$

$$+ k^{+}_{\mathrm{C3bBbC3b}}[\mathrm{C3bBbP}_{\mathrm{pathogen}}][\mathrm{npC3b}] + k^{+}_{\mathrm{C5b7}}[\mathrm{C3bBbC3bPC5bC6}_{\mathrm{pathogen}}][\mathrm{C7}] - k^{-}_{\mathrm{C3bP}}[\mathrm{C3bBbC3bP}_{\mathrm{pathogen}}]$$

$$+ k^{-}_{\mathrm{C5b}}[\mathrm{C3bBbC3bPC5b}_{\mathrm{pathogen}}] - k^{+}_{\mathrm{C3bBbC3bC5}}[\mathrm{C3bBbC3bP}_{\mathrm{pathogen}}][\mathrm{C5}] + k^{-}_{\mathrm{C3bBbC3bC5}}[\mathrm{C3bBbC3bPC5}_{\mathrm{pathogen}}]$$

6

$$\frac{d[\mathrm{C3bBbC3bP*}_{\mathrm{pathogen}}]}{dt} = k^{+}_{\mathrm{C3bBbC3b}}[\mathrm{C3bBbP*}_{\mathrm{pathogen}}][\mathrm{npC3b}] + k^{+}_{\mathrm{C3bBbC3b}}[\mathrm{C3bBbP*}_{\mathrm{pathogen}}][\mathrm{fC3b}] - k^{-}_{\mathrm{C3bP}}[\mathrm{C3bBbC3bP}_{\mathrm{pathogen}}]$$

$$- k^{+}_{\mathrm{C3bBbC3bC5}}[\mathrm{C3bBbC3bP*}_{\mathrm{pathogen}}][\mathrm{C5}] + k^{-}_{\mathrm{C3bBbC3bC5}}[\mathrm{C3bBbC3bPC5*}_{\mathrm{pathogen}}] + k^{-}_{\mathrm{C5b}}[\mathrm{C3bBbC3bPC5b*}_{\mathrm{pathogen}}]$$

$$+ k^{+}_{\mathrm{C5b7}}[\mathrm{C3bBbC3bPC5bC6*}_{\mathrm{pathogen}}][\mathrm{C7}] - k^{-}_{\mathrm{P*}_{\mathrm{surface}}}[\mathrm{C3bBbC3bP*}_{\mathrm{pathogen}}]$$

7

$$\frac{d[\mathrm{C3bBbC3bPC5}_{\mathrm{pathogen}}]}{dt} = k^{+}_{\mathrm{C3bBbC3bC5}}[\mathrm{C3bBbC3bP}_{\mathrm{pathogen}}][\mathrm{C5}] - k^{-}_{\mathrm{C3bBbC3bC5}}[\mathrm{C3bBbC3bPC5}_{\mathrm{pathogen}}]$$

$$- k^{\mathrm{kcat}}_{\mathrm{C3bBbC3bC5}}[\mathrm{C3bBbC3bPC5}_{\mathrm{pathogen}}] - k^{-}_{\mathrm{C3bP}}[\mathrm{C3bBbC3bPC5}_{\mathrm{pathogen}}]$$

8

$$\frac{d[\mathrm{C3bBbC3bPC5*}_{\mathrm{pathogen}}]}{dt} = k^{+}_{\mathrm{C3bBbC3bC5}}[\mathrm{C3bBbC3bP*}_{\mathrm{pathogen}}][\mathrm{C5}] - k^{-}_{\mathrm{C3bBbC3bC5}}[\mathrm{C3bBbC3bPC5*}_{\mathrm{pathogen}}]$$

$$- k^{\mathrm{kcat}}_{\mathrm{C3bBbC3bC5}}[\mathrm{C3bBbC3bPC5*}_{\mathrm{pathogen}}] - k^{-}_{\mathrm{P*}_{\mathrm{surface}}}[\mathrm{C3bBbC3bPC5*}_{\mathrm{pathogen}}]$$

9

$$\frac{\mathrm{d}[\mathrm{C3bBbC3bPC5b}_{\mathrm{pathogen}}]}{dt} = k^{\mathrm{kcat}}_{\mathrm{C3bBbC3bC5}}[\mathrm{C3bBbC3bP}_{\mathrm{pathogen}}][\mathrm{C5}] - k^{-}_{\mathrm{C5b}}[\mathrm{C3bBbC3bPC5b}_{\mathrm{pathogen}}] - k^{-}_{\mathrm{C3bP}}[\mathrm{C3bBbC3bPC5b}_{\mathrm{pathogen}}]$$

$$- k^{+}_{\mathrm{C3bBbC3bC5bC6}}[\mathrm{C3bBbC3bPC5b}_{\mathrm{pathogen}}][\mathrm{C6}] + k^{-}_{\mathrm{C3bBbC3bC5bC6}}[\mathrm{C3bBbC3bPC5bC6}_{\mathrm{pathogen}}]$$

10

$$\frac{d[\mathrm{C3bBbC3bPC5b*}_{\mathrm{pathogen}}]}{dt} = k^{\mathrm{kcat}}_{\mathrm{C3bBbC3bC5}}[\mathrm{C3bBbC3bP*}_{\mathrm{pathogen}}][\mathrm{C5}] - k^{-}_{\mathrm{C5b}}[\mathrm{C3bBbC3bPC5b*}_{\mathrm{pathogen}}]$$

$$- k^{+}_{\mathrm{C3bBbC3bC5bC6}}[\mathrm{C3bBbC3bPC5b*}_{\mathrm{pathogen}}][\mathrm{C6}] + k^{-}_{\mathrm{C3bBbC3bC5bC6}}[\mathrm{C3bBbC3bPC5bC6*}_{\mathrm{pathogen}}]$$

$$- k^{-}_{\mathrm{P*}_{\mathrm{surface}}}[\mathrm{C3bBbC3bPC5b*}_{\mathrm{pathogen}}]$$

11

$$\frac{d[\mathrm{C3bBbC3bPC5bC6}_{\mathrm{pathogen}}]}{dt} = k^{+}_{\mathrm{C3bBbC3bC5bC6}}[\mathrm{C3bBbC3bPC5b}_{\mathrm{pathogen}}][\mathrm{C6}] - k^{-}_{\mathrm{C3bBbC3bC5bC6}}[\mathrm{C3bBbC3bPC5bC6}_{\mathrm{pathogen}}]$$
$$- k^{+}_{\mathrm{C5b7}}[\mathrm{C3bBbC3bPC5bC6}_{\mathrm{pathogen}}][\mathrm{C7}] - k^{-}_{\mathrm{C3bP}}[\mathrm{C3bBbC3bPC5C6}_{\mathrm{pathogen}}]$$

12

$$\frac{d[\mathrm{C3bBbC3bPC5bC6*}_{\mathrm{pathogen}}]}{dt} = k^{+}_{\mathrm{C3bBbC3bC5bC6}}[\mathrm{C3bBbC3bPC5b*}_{\mathrm{pathogen}}][\mathrm{C6}] - k^{-}_{\mathrm{C3bBbC3bC5bC6}}[\mathrm{C3bBbC3bPC5bC6*}_{\mathrm{pathogen}}]$$
$$- k^{+}_{\mathrm{C5b7}}[\mathrm{C3bBbC3bPC5bC6*}_{\mathrm{pathogen}}][\mathrm{C7}] - k^{-}_{\mathrm{P*}_{\mathrm{surface}}}[\mathrm{C3bBbC3bPC5bC6*}_{\mathrm{pathogen}}]$$

13

$$\frac{d[\mathrm{pC5b7}_{\mathrm{fluid}}]}{dt} = k^{+}_{\mathrm{C5b7}}[\mathrm{C3bBbC3bC5bC6}_{\mathrm{pathogen}}][\mathrm{C7}] + k^{+}_{\mathrm{C5b7}}[\mathrm{C3bBbC3bPC5bC6}_{\mathrm{pathogen}}][\mathrm{C7}] + k^{+}_{\mathrm{C5b7}}[\mathrm{C3bBbC3bPC5bC6*}_{\mathrm{pathogen}}][\mathrm{C7}]$$
$$- k^{-}_{\mathrm{C5b7}}[\mathrm{pC5b7}_{\mathrm{fluid}}] - k^{+}_{\mathrm{VnC5b7}}[\mathrm{pC5b7}_{\mathrm{fluid}}][\mathrm{Vn}] + k^{-}_{\mathrm{VnC5b7}}[\mathrm{VnpC5b7}_{\mathrm{fluid}}] - k^{+}_{\mathrm{CnC5b7}}[\mathrm{pC5b7}_{\mathrm{fluid}}][\mathrm{Cn}] + k^{-}_{\mathrm{CnC5b7}}[\mathrm{CnpC5b7}_{\mathrm{fluid}}]$$
$$- k^{+}_{\mathrm{C5b8}_{\mathrm{fluid}}}[\mathrm{pC5b7}_{\mathrm{fluid}}][\mathrm{C8}] + k^{-}_{\mathrm{C5b8}}[\mathrm{C5b8}_{\mathrm{fluid}}] - k^{+}_{\mathrm{C5b7}_{\mathrm{surface}}}[\mathrm{pC5b7}_{\mathrm{fluid}}][\mathrm{Surface}_{\mathrm{pathogen}}] \times 4 \times (3.10 \times 10^{9}) - k^{+}_{\mathrm{C5b7}_{\mathrm{micelle}}}[\mathrm{pC5b7}_{\mathrm{fluid}}]$$

14

$$\frac{d[\mathrm{C5b7}_{\mathrm{micelle}}]}{dt} = k^{+}_{\mathrm{C5b7}_{\mathrm{micelle}}}[\mathrm{pC5b7}_{\mathrm{fluid}}] + k^{+}_{\mathrm{C5b7}_{\mathrm{micelle}}}[\mathrm{hC5b7}_{\mathrm{fluid}}]$$

15

$$\frac{d[\mathrm{C5b8}_{\mathrm{fluid}}]}{dt} = k^{+}_{\mathrm{C5b8}}[\mathrm{hC5b7}_{\mathrm{fluid}}][\mathrm{C8}] + k^{+}_{\mathrm{C5b8}}[\mathrm{pC5b7}_{\mathrm{fluid}}][\mathrm{C8}] - k^{-}_{\mathrm{C5b8}}[\mathrm{C5b8}_{\mathrm{fluid}}] - k^{+}_{\mathrm{C5b9}}[\mathrm{C5b8}_{\mathrm{fluid}}][\mathrm{C9}_{1}] + k^{-}_{\mathrm{C5b9}_{\mathrm{fluid}}}[\mathrm{C5b9}_{1\mathrm{fluid}}]$$

16

$$\frac{d[\mathrm{C5b9}_{1\mathrm{fluid}}]}{dt} = k^{+}_{\mathrm{C5b9}_{\mathrm{fluid}}}[\mathrm{C5b8}_{\mathrm{fluid}}][\mathrm{C9}_{1}] - k^{-}_{\mathrm{C5b9}_{\mathrm{fluid}}}[\mathrm{C5b9}_{1\mathrm{fluid}}]$$

17

$$\frac{d[\mathrm{C5b7}_{\mathrm{pathogen}}]}{dt} = k^{+}_{\mathrm{C5b7}_{\mathrm{surface}}}[\mathrm{pC5b7}_{\mathrm{fluid}}][\mathrm{Surface}_{\mathrm{pathogen}}] \times 4 \times (3.10 \times 10^{9}) - k^{+}_{\mathrm{C5b8}}[\mathrm{C5b7}_{\mathrm{pathogen}}][\mathrm{C8}]$$

18

$$\frac{d[\mathrm{C5b8}_{\mathrm{pathogen}}]}{dt} = k^{+}_{\mathrm{C5b8}}[\mathrm{C5b7}_{\mathrm{pathogen}}][\mathrm{C8}] - k^{+}_{\mathrm{C5b9}}[\mathrm{C5b8}_{\mathrm{pathogen}}][\mathrm{C9}_{18}]$$

19

$$\frac{d[\mathrm{MAC}_{\mathrm{pathogen}}]}{dt} = k^{+}_{\mathrm{C5b9}}[\mathrm{C5b8}_{\mathrm{pathogen}}][\mathrm{C9}_{18}]$$

(iv) Regulation (host cell and fluid state)

1

$$\frac{d[\mathrm{C3(H_2O)H_{fluid}}]}{dt} = k^{+}_{\mathrm{C3bH}}[\mathrm{C3(H_2O)_{fluid}}][\mathrm{H}] + k^{-}_{\mathrm{C3bH}}[\mathrm{C3(H_2O)H_{fluid}}]$$

2

$$\frac{d[\mathrm{C3(H_2O)CR1_{fluid}}]}{dt} = k^{+}_{\mathrm{C3bCR1}}[\mathrm{C3(H_2O)_{fluid}}][\mathrm{CR1}] + k^{-}_{\mathrm{C3bCR1}}[\mathrm{C3(H_2O)CR1_{fluid}}]$$

3

$$\frac{d[\mathrm{C3(H_2O)BbH_{fluid}}]}{dt} = k^{+}_{\mathrm{C3bH}}[\mathrm{C3(H_2O)Bb_{fluid}}][\mathrm{H}] - k^{-}_{\mathrm{C3bBbH_{decay}}}[\mathrm{C3(H_2O)BbH_{fluid}}]$$

4

$$\frac{d[\mathrm{nhC3b}]}{dt} = \frac{k^{\mathrm{C3}}_{\mathrm{catC3bBb}}[\mathrm{C3}][\mathrm{C3bBb_{host}}]}{\mathrm{K}^{\mathrm{C3}}_{\mathrm{mC3bBb}} + [\mathrm{C3}]} - k^{+}_{\mathrm{fC3b}}[\mathrm{nhC3b}][\mathrm{H_2O}] - k^{+}_{\mathrm{hC3b}}[\mathrm{nhC3b}][\mathrm{Surface_{host}}]*15.6 - k^{+}_{\mathrm{C3bBbC3b}}[\mathrm{C3bBb_{host}}][\mathrm{nhC3b}]$$

5

$$\frac{d[\mathrm{Surface_{host}}]}{dt} = -k^{+}_{\mathrm{C3b_{surface}}}[\mathrm{nfC3b}][\mathrm{Surface_{host}}] - k^{+}_{\mathrm{C3b_{surface}}}[\mathrm{nhC3b}][\mathrm{Surface_{host}}]\times 15.6$$
$$- k^{+}_{\mathrm{C5b7_{surface}}}[\mathrm{hC5b7_{fluid}}][\mathrm{Surface_{host}}]\times 4\times 1.4$$

6

$$\frac{d[\mathrm{C3b_{host}}]}{dt} = k^{+}_{\mathrm{C3b_{surface}}}[\mathrm{nfC3b}][\mathrm{Surface_{host}}] + k^{+}_{\mathrm{C3b_{surface}}}[\mathrm{nhC3b}][\mathrm{Surface_{host}}]*15.6 - k^{+}_{\mathrm{C3bCR1}}[\mathrm{C3b_{host}}][\mathrm{CR1}] - k^{-}_{\mathrm{C3bCR1}}[\mathrm{C3bCR1_{host}}]$$
$$- k^{+}_{\mathrm{C3bH}}[\mathrm{C3b_{host}}][\mathrm{H}] + k^{-}_{\mathrm{C3bH}}[\mathrm{C3bH_{host}}] - k^{+}_{\mathrm{C3bB}}[\mathrm{C3b_{host}}][\mathrm{B}] + k^{-}_{\mathrm{C3bB}}[\mathrm{C3bB_{host}}] + k^{-}_{\mathrm{C3bBbH_{decay}}}[\mathrm{C3bBbH_{host}}]$$
$$+ k^{-}_{\mathrm{C3bBbCR1_{decay}}}[\mathrm{C3bBbCR1_{host}}] + k^{-}_{\mathrm{C3bBbDAF_{decay}}}[\mathrm{C3bBbDAF_{host}}] + k^{-}_{\mathrm{C3bBbH_{decay}}}[\mathrm{C3bBbC3bH_{host}}] + k^{-}_{\mathrm{C3bBbC3b}}[\mathrm{C3bBbC3b_{host}}]$$
$$+ k^{-}_{\mathrm{C3bBbCR1_{decay}}}[\mathrm{C3bBbC3bCR1_{host}}] + k^{-}_{\mathrm{C3bBbDAF_{decay}}}[\mathrm{C3bBbC3bDAF_{host}}]$$

7

$$\frac{d[\mathrm{C3bB_{host}}]}{dt} = k^{+}_{\mathrm{C3bB}}[\mathrm{C3b_{host}}][\mathrm{B}] - k^{-}_{\mathrm{C3bB}}[\mathrm{C3bB_{host}}]$$
$$- \frac{k^{\mathrm{D}}_{\mathrm{catC3bB}}[\mathrm{D}][\mathrm{C3bB_{host}}]}{K^{\mathrm{D}}_{\mathrm{mC3(H_2O)B}} + [\mathrm{C3bB_{fluid}}] + [\mathrm{C3(H_2O)B_{fluid}}] + [\mathrm{C3bB_{host}}] + [\mathrm{C3bB_{pathogen}}] + [\mathrm{C3bBP_{pathogen}}] + [\mathrm{C3bBP*_{pathogen}}]}$$

8

$$\frac{d[\mathrm{C3bBb_{host}}]}{dt} = -k^{+}_{\mathrm{C3bBbC3b}}[\mathrm{C3bBb_{host}}][\mathrm{fC3b}] - k^{+}_{\mathrm{C3bBbC3b}}[\mathrm{C3bBb_{host}}][\mathrm{nhC3b}] - k^{+}_{\mathrm{C3bH}}[\mathrm{C3bBb_{host}}][\mathrm{H}] - k^{+}_{\mathrm{C3bCR1}}[\mathrm{C3bBb_{host}}][\mathrm{CR1}]$$

$$- k^{+}_{\mathrm{C3bBbDAF}}[\mathrm{C3bBb_{host}}][\mathrm{DAF}]$$

$$+ \frac{k^{\mathrm{D}}_{\mathrm{catC3bB}}[\mathrm{D}][\mathrm{C3bB_{host}}]}{K^{\mathrm{D}}_{\mathrm{mC3(H_2O)B}} + [\mathrm{C3bB_{fluid}}] + [\mathrm{C3(H_2O)B_{fluid}}] + [\mathrm{C3bB_{host}}] + [\mathrm{C3bB_{pathogen}}] + [\mathrm{C3bBP_{pathogen}}] + [\mathrm{C3bBP^{*}_{pathogen}}]}$$

9

$$\frac{d[\mathrm{C3bBbH_{host}}]}{dt} = k^{+}_{\mathrm{C3bH}}[\mathrm{C3bBb_{host}}][\mathrm{H}] - k^{-}_{\mathrm{C3bBbH_{decay}}}[\mathrm{C3bBbH_{host}}]$$

10

$$\frac{d[\mathrm{C3bBbCR1_{host}}]}{dt} = k^{+}_{\mathrm{C3bCR1}}[\mathrm{C3bBb_{host}}][\mathrm{CR1}] - k^{-}_{\mathrm{C3bBbCR1_{decay}}}[\mathrm{C3bBbCR1_{host}}]$$

11

$$\frac{d[\mathrm{C3bBbDAF_{host}}]}{dt} = k^{+}_{\mathrm{C3bBbDAF}}[\mathrm{C3bBb_{host}}][\mathrm{DAF}] - k^{-}_{\mathrm{C3bBbDAF_{decay}}}[\mathrm{C3bBbDAF_{host}}]$$

12

$$\frac{d[\mathrm{C3bCR1_{host}}]}{dt} = k^{+}_{\mathrm{C3bCR1}}[\mathrm{C3b_{host}}][\mathrm{CR1}] - k^{-}_{\mathrm{C3bCR1}}[\mathrm{C3bCR1_{host}}]$$

$$- \frac{k^{\mathrm{FI}}_{\mathrm{catC3bH}}[\mathrm{I}][\mathrm{C3bCR1_{host}}]}{K^{\mathrm{FI}}_{\mathrm{mC3bH}} + [\mathrm{C3bH_{fluid}}] + [\mathrm{C3bCR1_{fluid}}] + [\mathrm{iC3bCR1_{fluid}}] + [\mathrm{C3bH_{host}}] + [\mathrm{C3bCR1_{host}}] + [\mathrm{iC3bCR1_{host}}] + [\mathrm{iC3bCR1_{pathogen}}] + [\mathrm{C3bCR1_{pathogen}}]}$$

13

$$\frac{d[\mathrm{iC3b_{host}}]}{dt} = -k^{+}_{\mathrm{iC3bCR1}}[\mathrm{iC3b_{host}}][\mathrm{CR1}] + k^{-}_{\mathrm{iC3bCR1}}[\mathrm{iC3bCR1_{host}}]$$

$$+ \frac{k^{\mathrm{FI}}_{\mathrm{catC3bH}}[\mathrm{I}][\mathrm{C3bH_{host}}]}{K^{\mathrm{FI}}_{\mathrm{mC3bH}} + [\mathrm{C3bH_{fluid}}] + [\mathrm{C3bCR1_{fluid}}] + [\mathrm{iC3bCR1_{fluid}}] + [\mathrm{C3bH_{host}}] + [\mathrm{C3bCR1_{host}}] + [\mathrm{iC3bCR1_{host}}] + [\mathrm{iC3bCR1_{pathogen}}] + [\mathrm{C3bCR1_{pathogen}}]}$$

$$+ \frac{k^{\mathrm{FI}}_{\mathrm{catC3bH}}[\mathrm{I}][\mathrm{C3bCR1_{host}}]}{K^{\mathrm{FI}}_{\mathrm{mC3bH}} + [\mathrm{C3bH_{fluid}}] + [\mathrm{C3bCR1_{fluid}}] + [\mathrm{iC3bCR1_{fluid}}] + [\mathrm{C3bH_{host}}] + [\mathrm{C3bCR1_{host}}] + [\mathrm{iC3bCR1_{host}}] + [\mathrm{iC3bCR1_{pathogen}}] + [\mathrm{C3bCR1_{pathogen}}]}$$

14

$$\frac{d[\mathrm{C3bH_{host}}]}{dt} = k^{+}_{\mathrm{C3bH}}[\mathrm{C3b_{host}}][\mathrm{H}] - k^{-}_{\mathrm{C3bH_{host}}}[\mathrm{C3bH_{host}}]$$

$$- \frac{k^{\mathrm{FI}}_{\mathrm{catC3bH}}[\mathrm{I}][\mathrm{C3bH_{host}}]}{K^{\mathrm{FI}}_{\mathrm{mC3bH}} + [\mathrm{C3bH_{fluid}}] + [\mathrm{C3bCR1_{fluid}}] + [\mathrm{iC3bCR1_{fluid}}] + [\mathrm{C3bH_{host}}] + [\mathrm{C3bCR1_{host}}] + [\mathrm{iC3bCR1_{host}}] + [\mathrm{iC3bCR1_{pathogen}}] + [\mathrm{C3bCR1_{pathogen}}]}$$

15

$$\frac{d[\text{iC3bCR1}_{\text{host}}]}{dt} = k^{+}_{\text{iC3bCR1}}[\text{iC3b}_{\text{host}}][\text{CR1}] - k^{-}_{\text{iC3bCR1}}[\text{iC3bCR1}_{\text{host}}]$$
$$- \frac{k^{\text{FI}}_{\text{catC3bH}}[\text{I}][\text{iC3bCR1}_{\text{host}}]}{K^{\text{FI}}_{\text{mC3bH}} + [\text{C3bH}_{\text{fluid}}] + [\text{C3bCR1}_{\text{fluid}}] + [\text{iC3bCR1}_{\text{fluid}}] + [\text{C3bH}_{\text{host}}] + [\text{C3bCR1}_{\text{host}}] + [\text{iC3bCR1}_{\text{host}}] + [\text{iC3bCR1}_{\text{pathogen}}] + [\text{C3bCR1}_{\text{pathogen}}]}$$

16

$$\frac{d[\text{C3dg}_{\text{host}}]}{dt} =$$
$$\frac{k^{\text{FI}}_{\text{catC3bH}}[\text{I}][\text{iC3bCR1}_{\text{host}}]}{K^{\text{FI}}_{\text{mC3bH}} + [\text{C3bH}_{\text{fluid}}] + [\text{C3bCR1}_{\text{fluid}}] + [\text{iC3bCR1}_{\text{fluid}}] + [\text{C3bH}_{\text{host}}] + [\text{C3bCR1}_{\text{host}}] + [\text{iC3bCR1}_{\text{host}}] + [\text{iC3bCR1}_{\text{pathogen}}] + [\text{C3bCR1}_{\text{pathogen}}]}$$

17

$$\frac{d[\text{C3bH}_{\text{fluid}}]}{dt} = k^{+}_{\text{C3bH}}[\text{C3b}_{\text{fluid}}][\text{H}] - k^{-}_{\text{C3bH}}[\text{C3bH}_{\text{fluid}}]$$
$$- \frac{k^{\text{FI}}_{\text{catC3bH}}[\text{I}][\text{C3bH}_{\text{fluid}}]}{K^{\text{FI}}_{\text{mC3bH}} + [\text{C3bH}_{\text{fluid}}] + [\text{C3bCR1}_{\text{fluid}}] + [\text{iC3bCR1}_{\text{fluid}}] + [\text{C3bH}_{\text{host}}] + [\text{C3bCR1}_{\text{host}}] + [\text{iC3bCR1}_{\text{host}}] + [\text{iC3bCR1}_{\text{pathogen}}] + [\text{C3bCR1}_{\text{pathogen}}]}$$

18

$$\frac{d[\text{iC3b}_{\text{fluid}}]}{dt} = -k^{+}_{\text{iC3bCR1}}[\text{iC3b}_{\text{fluid}}][\text{CR1}] + k^{-}_{\text{iC3bCR1}}[\text{iC3bCR1}_{\text{fluid}}]$$
$$+ \frac{k^{\text{FI}}_{\text{catC3bH}}[\text{I}][\text{C3bH}_{\text{fluid}}]}{K^{\text{FI}}_{\text{mC3bH}} + [\text{C3bH}_{\text{fluid}}] + [\text{C3bCR1}_{\text{fluid}}] + [\text{iC3bCR1}_{\text{fluid}}] + [\text{C3bH}_{\text{host}}] + [\text{C3bCR1}_{\text{host}}] + [\text{iC3bCR1}_{\text{host}}] + [\text{iC3bCR1}_{\text{pathogen}}] + [\text{C3bCR1}_{\text{pathogen}}]}$$
$$+ \frac{k^{\text{FI}}_{\text{catC3bH}}[\text{I}][\text{C3bCR1}_{\text{fluid}}]}{K^{\text{FI}}_{\text{mC3bH}} + [\text{C3bH}_{\text{fluid}}] + [\text{C3bCR1}_{\text{fluid}}] + [\text{iC3bCR1}_{\text{fluid}}] + [\text{C3bH}_{\text{host}}] + [\text{C3bCR1}_{\text{host}}] + [\text{iC3bCR1}_{\text{host}}] + [\text{iC3bCR1}_{\text{pathogen}}] + [\text{C3bCR1}_{\text{pathogen}}]}$$

19

$$\frac{d[\text{C3bCR1}_{\text{fluid}}]}{dt} = k^{+}_{\text{C3bCR1}}[\text{C3b}_{\text{fluid}}][\text{CR1}] - k^{-}_{\text{C3bCR1}}[\text{C3bCR1}_{\text{fluid}}]$$
$$- \frac{k^{\text{FI}}_{\text{catC3bH}}[\text{I}][\text{C3bCR1}_{\text{fluid}}]}{K^{\text{FI}}_{\text{mC3bH}} + [\text{C3bH}_{\text{fluid}}] + [\text{C3bCR1}_{\text{fluid}}] + [\text{iC3bCR1}_{\text{fluid}}] + [\text{C3bH}_{\text{host}}] + [\text{C3bCR1}_{\text{host}}] + [\text{iC3bCR1}_{\text{host}}] + [\text{iC3bCR1}_{\text{pathogen}}] + [\text{C3bCR1}_{\text{pathogen}}]}$$

20

$$\frac{d[\text{iC3bCR1}_{\text{fluid}}]}{dt} = k^{+}_{\text{iC3bCR1}}[\text{iC3b}_{\text{fluid}}][\text{CR1}] - k^{-}_{\text{iC3bCR1}}[\text{iC3bCR1}_{\text{fluid}}]$$
$$- \frac{k^{\text{FI}}_{\text{catC3bH}}[\text{I}][\text{iC3bCR1}_{\text{fluid}}]}{K^{\text{FI}}_{\text{mC3bH}} + [\text{C3bH}_{\text{fluid}}] + [\text{C3bH}_{\text{host}}] + [\text{C3bCR1}_{\text{host}}] + [\text{iC3bCR1}_{\text{host}}] + [\text{iC3bCR1}_{\text{pathogen}}] + [\text{C3bCR1}_{\text{pathogen}}] + [\text{C3bCR1}_{\text{fluid}}] + [\text{iC3bCR1}_{\text{fluid}}]}$$

21

$$\frac{d[\mathrm{C3dg}_{\mathrm{fluid}}]}{dt} = \frac{k^{\mathrm{FI}}_{\mathrm{catC3bH}}[\mathrm{I}][\mathrm{iC3bCR1}_{\mathrm{fluid}}]}{K^{\mathrm{FI}}_{\mathrm{mC3bH}} + [\mathrm{C3bH}_{\mathrm{fluid}}] + [\mathrm{C3bCR1}_{\mathrm{fluid}}] + [\mathrm{iC3bCR1}_{\mathrm{fluid}}] + [\mathrm{C3bH}_{\mathrm{host}}] + [\mathrm{C3bCR1}_{\mathrm{host}}] + [\mathrm{iC3bCR1}_{\mathrm{host}}] + [\mathrm{iC3bCR1}_{\mathrm{pathogen}}] + [\mathrm{C3bCR1}_{\mathrm{pathogen}}]}$$

22

$$\frac{d[\mathrm{C3bBbH}_{\mathrm{fluid}}]}{dt} = k^{+}_{\mathrm{C3bH}}[\mathrm{C3bBb}_{\mathrm{fluid}}][\mathrm{H}] - k^{-}_{\mathrm{C3bBbH}_{\mathrm{decay}}}[\mathrm{C3bBbH}_{\mathrm{fluid}}]$$

23

$$\frac{d[\mathrm{C3bBbCR1}_{\mathrm{fluid}}]}{dt} = k^{+}_{\mathrm{C3bCR1}}[\mathrm{C3bBb}_{\mathrm{fluid}}][\mathrm{CR1}] - k^{-}_{\mathrm{C3bBbCR1}_{\mathrm{decay}}}[\mathrm{C3bBbCR1}_{\mathrm{fluid}}]$$

24

$$\frac{d[\mathrm{C3bBbC3b}_{\mathrm{host}}]}{dt} = k^{+}_{\mathrm{C3bBbC3b}}[\mathrm{C3bBb}_{\mathrm{host}}][\mathrm{nhC3b}] + k^{+}_{\mathrm{C3bBbC3b}}[\mathrm{C3bBb}_{\mathrm{host}}][\mathrm{fC3b}] - k^{-}_{\mathrm{C3bBbC3b}}[\mathrm{C3bBbC3b}_{\mathrm{host}}]$$
$$- k^{+}_{\mathrm{C3bCR1}}[\mathrm{C3bBbC3b}_{\mathrm{host}}][\mathrm{CR1}] - k^{+}_{\mathrm{C3bBbDAF}}[\mathrm{C3bBbC3b}_{\mathrm{host}}][\mathrm{DAF}] - k^{+}_{\mathrm{C3bH}}[\mathrm{C3bBbC3b}_{\mathrm{host}}][\mathrm{H}] - k^{+}_{\mathrm{C3bBbC3bC5}}[\mathrm{C3bBbC3b}_{\mathrm{host}}][\mathrm{C5}]$$
$$+ k^{-}_{\mathrm{C3bBbC3bC5}}[\mathrm{C3bBbC3bC5}_{\mathrm{host}}] + k^{-}_{\mathrm{C5b}}[\mathrm{C3bBbC3bC5b}_{\mathrm{host}}] + k^{+}_{\mathrm{C5bC6C7}}[\mathrm{C3bBbC3bC5bC6}_{\mathrm{host}}][\mathrm{C7}]$$

25

$$\frac{d[\mathrm{C3bBbC3bH}_{\mathrm{host}}]}{dt} = k^{+}_{\mathrm{C3bH}}[\mathrm{C3bBbC3b}_{\mathrm{host}}][\mathrm{H}] - k^{-}_{\mathrm{C3bBbH}_{\mathrm{decay}}}[\mathrm{C3bBbC3bH}_{\mathrm{host}}]$$

26

$$\frac{d[\mathrm{C3bBbC3bCR1}_{\mathrm{host}}]}{dt} = k^{+}_{\mathrm{C3bCR1}}[\mathrm{C3bBbC3b}_{\mathrm{host}}][\mathrm{CR1}] - k^{-}_{\mathrm{C3bBbCR1}_{\mathrm{decay}}}[\mathrm{C3bBbC3bCR1}_{\mathrm{host}}]$$

27

$$\frac{d[\mathrm{C3bBbC3bDAF}_{\mathrm{host}}]}{dt} = k^{+}_{\mathrm{C3bBbDAF}}[\mathrm{C3bBbC3b}_{\mathrm{host}}][\mathrm{DAF}] - k^{-}_{\mathrm{C3bBbDAF}_{\mathrm{decay}}}[\mathrm{C3bBbC3bDAF}_{\mathrm{host}}]$$

28

$$\frac{d[\mathrm{C3bBbC3bC5}_{\mathrm{host}}]}{dt} = k^{+}_{\mathrm{C3bBbC3bC5}}[\mathrm{C3bBbC3b}_{\mathrm{host}}][\mathrm{C5}] - k^{-}_{\mathrm{C3bBbC3bC5}}[\mathrm{C3bBbC3bC5}_{\mathrm{host}}]$$
$$- k^{\mathrm{kcat}}_{\mathrm{C3bBbC3bC5}}[\mathrm{C3bBbC3bC5}_{\mathrm{host}}]$$

29

$$\frac{d[\mathrm{C3bBbC3bC5b}_{\mathrm{host}}]}{dt} = k^{\mathrm{kcat}}_{\mathrm{C3bBbC3bC5}}[\mathrm{C3bBbC3bC5}_{\mathrm{host}}] - k^{-}_{\mathrm{C3bBbC3bC5b}}[\mathrm{C3bBbC3bC5b}_{\mathrm{host}}]$$
$$- k^{+}_{\mathrm{C3bBbC3bC5bC6}}[\mathrm{C3bBbC3bC5b}_{\mathrm{host}}][\mathrm{C6}] + k^{-}_{\mathrm{C3bBbC3bC5bC6}}[\mathrm{C3bBbC3bC5bC6}_{\mathrm{host}}]$$

30

$$\frac{d[\mathrm{C3bBbC3bC5bC6}_{\mathrm{host}}]}{dt} = k^{+}_{\mathrm{C3bBbC3bC5bC6}}[\mathrm{C3bBbC3bC5b}_{\mathrm{host}}][\mathrm{C6}] - k^{-}_{\mathrm{C3bBbC3bC5bC6}}[\mathrm{C3bBbC3bC5bC6}_{\mathrm{host}}]$$
$$- k^{+}_{\mathrm{C5b7}}[\mathrm{C3bBbC3bC5bC6}_{\mathrm{host}}][\mathrm{C7}]$$

31

$$\frac{d[\mathrm{hC5b7}_{\mathrm{fluid}}]}{dt} = k^{+}_{\mathrm{C5b7}}[\mathrm{C3bBbC3bC5bC6}_{\mathrm{host}}][\mathrm{C7}] - k^{+}_{\mathrm{C5b7}_{\mathrm{surface}}}[\mathrm{hC5b7}_{\mathrm{fluid}}][\mathrm{Surface}_{\mathrm{host}}] \times 4 \times 1.4 - k^{+}_{\mathrm{C5b7}_{\mathrm{micelle}}}[\mathrm{hC5b7}_{\mathrm{fluid}}]$$
$$- k^{+}_{\mathrm{C5b8}}[\mathrm{hC5b7}_{\mathrm{fluid}}][\mathrm{C8}] + k^{-}_{\mathrm{C5b8}}[\mathrm{C5b8}_{\mathrm{fluid}}] - k^{+}_{\mathrm{CnC5b7}}[\mathrm{hC5b7}_{\mathrm{fluid}}][\mathrm{Cn}] + k^{-}_{\mathrm{CnC5b7}}[\mathrm{CnhC5b7}_{\mathrm{fluid}}] - k^{+}_{\mathrm{VnC5b7}}[\mathrm{hC5b7}_{\mathrm{fluid}}][\mathrm{Vn}]$$
$$+ k^{-}_{\mathrm{VnC5b7}}[\mathrm{VnhC5b7}_{\mathrm{fluid}}]$$

32

$$\frac{d[\mathrm{C5b7}_{\mathrm{host}}]}{dt} = k^{+}_{\mathrm{C5b7}_{\mathrm{surface}}}[\mathrm{hC5b7}_{\mathrm{fluid}}][\mathrm{Surface}_{\mathrm{host}}] \times 4 \times 1.4 - k^{+}_{\mathrm{C5b8}}[\mathrm{C5b7}_{\mathrm{host}}][\mathrm{C8}]$$

33

$$\frac{d[\mathrm{C5b8}_{\mathrm{host}}]}{dt} = k^{+}_{\mathrm{C5b8}}[\mathrm{C5b7}_{\mathrm{host}}][\mathrm{C8}] - k^{+}_{\mathrm{C5b9}}[\mathrm{C5b8}_{\mathrm{host}}][\mathrm{C9}_{1}]$$

34

$$\frac{d[\mathrm{C5b9}_{1\mathrm{host}}]}{dt} = k^{+}_{\mathrm{C5b9}}[\mathrm{C5b8}_{\mathrm{host}}][\mathrm{C9}_{1}] - k^{+}_{\mathrm{C5b9}}[\mathrm{C5b9}_{1\mathrm{host}}][\mathrm{C9}_{17}] - k^{+}_{\mathrm{CD59C5b9}}[\mathrm{C5b9}_{1\mathrm{host}}][\mathrm{CD59}] + k^{-}_{\mathrm{CD59C5b9}}[\mathrm{CD59C5b9}_{1\mathrm{host}}]$$

35

$$\frac{d[\mathrm{MAC}_{\mathrm{host}}]}{dt} = k^{+}_{\mathrm{C5b9}}[\mathrm{C5b9}_{1\mathrm{host}}][\mathrm{C9}_{17}]$$

36

$$\frac{d[\mathrm{CD59C5b9}_{1\mathrm{host}}]}{dt} = k^{+}_{\mathrm{CD59C5b9}}[\mathrm{C5b9}_{1\mathrm{host}}][\mathrm{CD59}] - k^{-}_{\mathrm{CD59C5b9}}[\mathrm{CD59C5b9}_{1\mathrm{host}}]$$

37

$$\frac{d[\mathrm{VnC5b7}_{\mathrm{fluid}}]}{dt} = k^{+}_{\mathrm{VnC5b7}}[\mathrm{hC5b7}_{\mathrm{fluid}}][\mathrm{Vn}] + k^{+}_{\mathrm{VnC5b7}}[\mathrm{pC5b7}_{\mathrm{fluid}}][\mathrm{Vn}] - k^{-}_{\mathrm{VnC5b7}}[\mathrm{VnC5b7}_{\mathrm{fluid}}] - k^{+}_{\mathrm{VnC5b8}}[\mathrm{VnC5b7}_{\mathrm{fluid}}][\mathrm{C8}]$$
$$+ k^{-}_{\mathrm{VnC5b8}}[\mathrm{VnC5b8}_{\mathrm{fluid}}]$$

38

$$\frac{d[\mathrm{VnC5b8}_{\mathrm{fluid}}]}{dt} = k^{+}_{\mathrm{VnC5b8}}[\mathrm{VnC5b7}_{\mathrm{fluid}}][\mathrm{C8}] - k^{-}_{\mathrm{VnC5b8}}[\mathrm{VnC5b8}_{\mathrm{fluid}}] - k^{+}_{\mathrm{VnC5b9}}[\mathrm{VnC5b8}_{\mathrm{fluid}}][\mathrm{C9}_{1}] + k^{-}_{\mathrm{VnC5b9}}[\mathrm{VnC5b9}_{1\mathrm{fluid}}]$$

39

$$\frac{d[\mathrm{VnC5b9}_{1\mathrm{fluid}}]}{dt} = k^{+}_{\mathrm{VnC5b9}}[\mathrm{VnC5b8}_{\mathrm{fluid}}][\mathrm{C9}_{1}] - k^{-}_{\mathrm{VnC5b9}}[\mathrm{VnC5b9}_{1\mathrm{fluid}}]$$

40

$$\frac{d[\mathrm{CnC5b7}_{\mathrm{fluid}}]}{dt} = k^{+}_{\mathrm{CnC5b7}}[\mathrm{hC5b7}_{\mathrm{fluid}}][\mathrm{Cn}] + k^{+}_{\mathrm{CnC5b7}}[\mathrm{pC5b7}_{\mathrm{fluid}}][\mathrm{Cn}] - k^{-}_{\mathrm{CnC5b7}}[\mathrm{CnC5b7}_{\mathrm{fluid}}] - k^{+}_{\mathrm{CnC5b8}}[\mathrm{CnC5b7}_{\mathrm{fluid}}][\mathrm{C8}] + k^{-}_{\mathrm{CnC5b8}}[\mathrm{CnC5b8}_{\mathrm{fluid}}]$$

41

$$\frac{d[\mathrm{CnC5b8}_{\mathrm{fluid}}]}{dt} = k^{+}_{\mathrm{CnC5b8}}[\mathrm{CnC5b7}_{\mathrm{fluid}}][\mathrm{C8}] - k^{-}_{\mathrm{CnC5b8}}[\mathrm{CnC5b8}_{\mathrm{fluid}}] - k^{+}_{\mathrm{CnC5b9}}[\mathrm{CnC5b8}_{\mathrm{fluid}}][\mathrm{C9}_{1}] + k^{-}_{\mathrm{CnC5b9}}[\mathrm{CnC5b9}_{1\mathrm{fluid}}]$$

42

$$\frac{d[\mathrm{CnC5b9}_{1\mathrm{fluid}}]}{dt} = k^{+}_{\mathrm{CnC5b9}}[\mathrm{CnC5b8}_{\mathrm{fluid}}][\mathrm{C9}_{1}] - k^{-}_{\mathrm{CnC5b9}}[\mathrm{CnC5b9}_{1\mathrm{fluid}}]$$

(v) Complement Proteins (host cell and fluid state)

1

$$\frac{d[\mathrm{C3}]}{dt} = -k^{+}_{\mathrm{C3(H_2O)}}[\mathrm{C3}] - \frac{k^{\mathrm{C3}}_{\mathrm{catC3(H_2O)Bb}}[\mathrm{C3}][\mathrm{C3(H_2O)Bb}_{\mathrm{fluid}}]}{K^{\mathrm{C3}}_{\mathrm{mC3(H_2O)Bb}_{\mathrm{fluid}}} + [\mathrm{C3}]} - \frac{k^{\mathrm{C3}}_{\mathrm{catC3bBb}}[\mathrm{C3}][\mathrm{C3bBb}_{\mathrm{fluid}}]}{K^{\mathrm{C3}}_{\mathrm{mC3bBb}_{\mathrm{fluid}}} + [\mathrm{C3}]} - \frac{k^{\mathrm{C3}}_{\mathrm{catC3bBb}}[\mathrm{C3}][\mathrm{C3bBb}_{\mathrm{pathogen}}]}{K^{\mathrm{C3}}_{\mathrm{mC3bBb}} + [\mathrm{C3}]} - \frac{k^{\mathrm{C3}}_{\mathrm{catC3bBbP}}[\mathrm{C3}][\mathrm{C3bBbP}_{\mathrm{pathogen}}]}{K^{\mathrm{C3}}_{\mathrm{mC3bBbP}} + [\mathrm{C3}]} - \frac{k^{\mathrm{C3}}_{\mathrm{catC3bBbP}}[\mathrm{C3}][\mathrm{C3bBbP*}_{\mathrm{pathogen}}]}{K^{\mathrm{C3}}_{\mathrm{mC3bBbP}} + [\mathrm{C3}]} - \frac{k^{\mathrm{C3}}_{\mathrm{catC3bBb}}[\mathrm{C3}][\mathrm{C3bBb}_{\mathrm{host}}]}{K^{\mathrm{C3}}_{\mathrm{mC3bBb}} + [\mathrm{C3}]}$$

2

$$\frac{d[\mathrm{C3a}]}{dt} = \frac{k^{\mathrm{C3}}_{\mathrm{catC3(H_2O)Bb}}[\mathrm{C3}][\mathrm{C3(H_2O)Bb}_{\mathrm{fluid}}]}{K^{\mathrm{C3}}_{\mathrm{mC3(H_2O)Bb}_{\mathrm{fluid}}} + [\mathrm{C3}]} + \frac{k^{\mathrm{C3}}_{\mathrm{catC3bBb}}[\mathrm{C3}][\mathrm{C3bBb}_{\mathrm{fluid}}]}{K^{\mathrm{C3}}_{\mathrm{mC3bBb}_{\mathrm{fluid}}} + [\mathrm{C3}]} + \frac{k^{\mathrm{C3}}_{\mathrm{catC3bBb}}[\mathrm{C3}][\mathrm{C3bBb}_{\mathrm{pathogen}}]}{K^{\mathrm{C3}}_{\mathrm{mC3bBb}} + [\mathrm{C3}]} + \frac{k^{\mathrm{C3}}_{\mathrm{catC3bBbP}}[\mathrm{C3}][\mathrm{C3bBbP}_{\mathrm{pathogen}}]}{K^{\mathrm{C3}}_{\mathrm{mC3bBbP}} + [\mathrm{C3}]} + \frac{k^{\mathrm{C3}}_{\mathrm{catC3bBbP}}[\mathrm{C3}][\mathrm{C3bBbP*}_{\mathrm{pathogen}}]}{K^{\mathrm{C3}}_{\mathrm{mC3bBbP}} + [\mathrm{C3}]} + \frac{k^{\mathrm{C3}}_{\mathrm{catC3bBb}}[\mathrm{C3}][\mathrm{C3bBb}_{\mathrm{host}}]}{K^{\mathrm{C3}}_{\mathrm{mC3bBb}} + [\mathrm{C3}]}$$

3

$$\frac{d[\mathrm{B}]}{dt} = -k^{+}_{\mathrm{C3(H_2O)B}}[\mathrm{C3(H_2O)}_{\mathrm{fluid}}][\mathrm{B}] + k^{-}_{\mathrm{C3(H_2O)B}}[\mathrm{C3(H_2O)B}_{\mathrm{fluid}}] - k^{+}_{\mathrm{C3bB}}[\mathrm{C3b}_{\mathrm{fluid}}][\mathrm{B}] + k^{-}_{\mathrm{C3bB}}[\mathrm{C3bB}_{\mathrm{fluid}}] - k^{+}_{\mathrm{C3bB}}[\mathrm{C3b}_{\mathrm{pathogen}}][\mathrm{B}] + k^{-}_{\mathrm{C3bB}}[\mathrm{C3bB}_{\mathrm{pathogen}}] - k^{+}_{\mathrm{C3bB}}[\mathrm{C3bP*}_{\mathrm{pathogen}}][\mathrm{B}] - k^{+}_{\mathrm{C3bB}}[\mathrm{C3b}_{\mathrm{host}}][\mathrm{B}] + k^{-}_{\mathrm{C3bB}}[\mathrm{C3bB}_{\mathrm{host}}]$$

4

$$\frac{d[D]}{dt} = -\frac{k^{D}_{catC3(H_2O)B}[D][C3bB_{fluid}]}{K^{D}_{mC3(H_2O)B} + [C3bB_{fluid}] + [C3(H_2O)B_{fluid}] + [C3bB_{host}] + [C3bB_{pathogen}] + [C3bBP_{pathogen}] + [C3bBP*_{pathogen}]}$$

$$-\frac{k^{D}_{catC3(H_2O)B}[D][C3(H_2O)B_{fluid}]}{K^{D}_{mC3(H_2O)B} + [C3bB_{fluid}] + [C3(H_2O)B_{fluid}] + [C3bB_{host}] + [C3bB_{pathogen}] + [C3bBP_{pathogen}] + [C3bBP*_{pathogen}]}$$

$$-\frac{k^{D}_{catC3(H_2O)B}[D][C3bB_{host}]}{K^{D}_{mC3(H_2O)B} + [C3bB_{fluid}] + [C3(H_2O)B_{fluid}] + [C3bB_{host}] + [C3bB_{pathogen}] + [C3bBP_{pathogen}] + [C3bBP*_{pathogen}]}$$

$$-\frac{k^{D}_{catC3(H_2O)B}[D][C3bB_{pathogen}]}{K^{D}_{mC3(H_2O)B} + [C3bB_{fluid}] + [C3(H_2O)B_{fluid}] + [C3bB_{host}] + [C3bB_{pathogen}] + [C3bBP_{pathogen}] + [C3bBP*_{pathogen}]}$$

$$-\frac{k^{D}_{catC3(H_2O)B}[D][C3bBP_{pathogen}]}{K^{D}_{mC3(H_2O)B} + [C3bB_{fluid}] + [C3(H_2O)B_{fluid}] + [C3bB_{host}] + [C3bB_{pathogen}] + [C3bBP_{pathogen}] + [C3bBP*_{pathogen}]}$$

$$-\frac{k^{D}_{catC3(H_2O)B}[D][C3bBP*_{pathogen}]}{K^{D}_{mC3(H_2O)B} + [C3bB_{fluid}] + [C3(H_2O)B_{fluid}] + [C3bB_{host}] + [C3bB_{pathogen}] + [C3bBP_{pathogen}] + [C3bBP*_{pathogen}]}$$

5

$$\frac{d[I]}{dt} = -\frac{k^{FI}_{catC3bH}[I][C3bH_{fluid}]}{K^{FI}_{mC3bH} + [C3bH_{fluid}] + [C3bCR1_{fluid}] + [iC3bCR1_{fluid}] + [C3bH_{host}] + [C3bCR1_{host}] + [iC3bCR1_{host}] + [iC3bCR1_{pathogen}] + [C3bCR1_{pathogen}]}$$

$$-\frac{k^{FI}_{catC3bH}[I][C3bCR1_{fluid}]}{K^{FI}_{mC3bH} + [C3bH_{fluid}] + [C3bCR1_{fluid}] + [iC3bCR1_{fluid}] + [C3bH_{host}] + [C3bCR1_{host}] + [iC3bCR1_{host}] + [iC3bCR1_{pathogen}] + [C3bCR1_{pathogen}]}$$

$$-\frac{k^{FI}_{catC3bH}[I][iC3bCR1_{fluid}]}{K^{FI}_{mC3bH} + [C3bH_{fluid}] + [C3bCR1_{fluid}] + [iC3bCR1_{fluid}] + [C3bH_{host}] + [C3bCR1_{host}] + [iC3bCR1_{host}] + [iC3bCR1_{pathogen}] + [C3bCR1_{pathogen}]}$$

$$-\frac{k^{FI}_{catC3bH}[I][C3bH_{host}]}{K^{FI}_{mC3bH} + [C3bH_{fluid}] + [C3bCR1_{fluid}] + [iC3bCR1_{fluid}] + [C3bH_{host}] + [C3bCR1_{host}] + [iC3bCR1_{host}] + [iC3bCR1_{pathogen}] + [C3bCR1_{pathogen}]}$$

$$-\frac{k^{FI}_{catC3bH}[I][C3bCR1_{host}]}{K^{FI}_{mC3bH} + [C3bH_{fluid}] + [C3bCR1_{fluid}] + [iC3bCR1_{fluid}] + [C3bH_{host}] + [C3bCR1_{host}] + [iC3bCR1_{host}] + [iC3bCR1_{pathogen}] + [C3bCR1_{pathogen}]}$$

$$-\frac{k^{FI}_{catC3bH}[I][iC3bCR1_{host}]}{K^{FI}_{mC3bH} + [C3bH_{fluid}] + [C3bCR1_{fluid}] + [iC3bCR1_{fluid}] + [C3bH_{host}] + [C3bCR1_{host}] + [iC3bCR1_{host}] + [iC3bCR1_{pathogen}] + [C3bCR1_{pathogen}]}$$

$$-\frac{k^{FI}_{catC3bH}[I][iC3bCR1_{pathogen}]}{K^{FI}_{mC3bH} + [C3bH_{fluid}] + [C3bCR1_{fluid}] + [iC3bCR1_{fluid}] + [C3bH_{host}] + [C3bCR1_{host}] + [iC3bCR1_{host}] + [iC3bCR1_{pathogen}] + [C3bCR1_{pathogen}]}$$

$$-\frac{k^{FI}_{catC3bH}[I][C3bCR1_{pathogen}]}{K^{FI}_{mC3bH} + [C3bH_{fluid}] + [C3bCR1_{fluid}] + [iC3bCR1_{fluid}] + [C3bH_{host}] + [C3bCR1_{host}] + [iC3bCR1_{host}] + [iC3bCR1_{pathogen}] + [C3bCR1_{pathogen}]}$$

6

$$\frac{d[\mathrm{P}]}{dt} = -k^{+}_{\mathrm{C3bP}}[\mathrm{C3bBb}_{\mathrm{pathogen}}][\mathrm{P}] + k^{-}_{\mathrm{C3bBbP}}[\mathrm{C3bBbP}_{\mathrm{pathogen}}] - k^{+}_{\mathrm{C3bP}}[\mathrm{C3bBbC3b}_{\mathrm{pathogen}}][\mathrm{P}] - k^{+}_{\mathrm{iC3b\,P}}[\mathrm{iC3b}_{\mathrm{pathogen}}][\mathrm{P}]$$
$$+ k^{-}_{\mathrm{iC3bP}}[\mathrm{iC3bP}_{\mathrm{pathogen}}] + k^{-}_{\mathrm{C3bP}}[\mathrm{C3bBbC3bP}_{\mathrm{pathogen}}] + k^{-}_{\mathrm{C3bP}}[\mathrm{C3bBbC3bC5P}_{\mathrm{pathogen}}] + k^{-}_{\mathrm{C3bP}}[\mathrm{C3bBbC3bC5bP}_{\mathrm{pathogen}}]$$
$$+ k^{-}_{\mathrm{C3bP}}[\mathrm{C3bBbC3bC5bC6P}_{\mathrm{pathogen}}]$$

7

$$\frac{d[\mathrm{P}^*]}{dt} = k^{+}_{\mathrm{P}^*\,\mathrm{released}}[\mathrm{P}^*_{\mathrm{neutrophil}}] - k^{+}_{\mathrm{P}^*_{\mathrm{surface}}}[\mathrm{P}^*][\mathrm{Surface}_{\mathrm{pathogen}}] + k^{-}_{\mathrm{P}^*}[\mathrm{PSurface}^*_{\mathrm{pathogen}}] - k^{+}_{\mathrm{C3bP}}[\mathrm{C3b}_{\mathrm{pathogen}}][\mathrm{P}^*]$$
$$+ k^{-}_{\mathrm{C3bP}}[\mathrm{C3bP}^*_{\mathrm{pathogen}}] - k^{+}_{\mathrm{C3bP}}[\mathrm{C3bB}_{\mathrm{pathogen}}][\mathrm{P}^*] + k^{-}_{\mathrm{C3bP}}[\mathrm{C3bBP}^*_{\mathrm{pathogen}}] - k^{+}_{\mathrm{C3bP}}[\mathrm{C3bBb}_{\mathrm{pathogen}}][\mathrm{P}^*]$$
$$+ k^{-}_{\mathrm{C3bBbP}}[\mathrm{C3bBbP}^*_{\mathrm{pathogen}}] - k^{+}_{\mathrm{C3bP}}[\mathrm{C3bBbC3b}_{\mathrm{pathogen}}][\mathrm{P}^*] + k^{-}_{\mathrm{C3bP}}[\mathrm{C3bBbC3bP}^*_{\mathrm{pathogen}}] + k^{-}_{\mathrm{C3bP}}[\mathrm{C3bBbC3bC5P}^*_{\mathrm{pathogen}}]$$
$$+ k^{-}_{\mathrm{C3bP}}[\mathrm{C3bBbC3bC5bP}^*_{\mathrm{pathogen}}] + k^{-}_{\mathrm{C3bP}}[\mathrm{C3bBbC3bC5bC6P}^*_{\mathrm{pathogen}}] + k^{-}_{\mathrm{P}^*_{\mathrm{surface}}}[\mathrm{C3bP}^*_{\mathrm{pathogen}}]$$
$$+ k^{-}_{\mathrm{P}^*_{\mathrm{surface}}}[\mathrm{C3bBP}^*_{\mathrm{pathogen}}] + k^{-}_{\mathrm{P}^*_{\mathrm{surface}}}[\mathrm{C3bBbP}^*_{\mathrm{pathogen}}] + k^{-}_{\mathrm{P}^*_{\mathrm{surface}}}[\mathrm{C3bBbC3bP}^*_{\mathrm{pathogen}}]$$
$$+ k^{-}_{\mathrm{P}^*_{\mathrm{surface}}}[\mathrm{C3bBbC3bC5P}^*_{\mathrm{pathogen}}] + k^{-}_{\mathrm{P}^*_{\mathrm{surface}}}[\mathrm{C3bBbC3bC5bP}^*_{\mathrm{pathogen}}] + k^{-}_{\mathrm{P}^*_{\mathrm{surface}}}[\mathrm{C3bBbC3bC5bPC6}^*_{\mathrm{pathogen}}]$$

8

$$\frac{d[\mathrm{H}]}{dt} = -k^{+}_{\mathrm{C3bH}}[\mathrm{C3(H_2O)}_{\mathrm{fluid}}][\mathrm{H}] + k^{-}_{\mathrm{C3bH}}[\mathrm{C3(H_2O)H}_{\mathrm{fluid}}] - k^{+}_{\mathrm{C3bH}}[\mathrm{C3(H_2O)Bb}_{\mathrm{fluid}}][\mathrm{H}] + k^{-}_{\mathrm{C3bBbH}_{\mathrm{decay}}}[\mathrm{C3(H_2O)BbH}_{\mathrm{fluid}}] - k^{+}_{\mathrm{C3bH}}[\mathrm{C3b}_{\mathrm{fluid}}][\mathrm{H}]$$
$$+ k^{-}_{\mathrm{C3bH}}[\mathrm{C3bH}_{\mathrm{fluid}}] - k^{+}_{\mathrm{C3bH}}[\mathrm{C3bBb}_{\mathrm{fluid}}][\mathrm{H}] + k^{-}_{\mathrm{C3bBbH}_{\mathrm{decay}}}[\mathrm{C3bBbH}_{\mathrm{fluid}}] - k^{+}_{\mathrm{C3bH}}[\mathrm{C3b}_{\mathrm{host}}][\mathrm{H}] + k^{-}_{\mathrm{C3bH}}[\mathrm{C3bH}_{\mathrm{host}}] - k^{+}_{\mathrm{C3bH}}[\mathrm{C3bBb}_{\mathrm{host}}][\mathrm{H}]$$
$$+ k^{-}_{\mathrm{C3bBbH}_{\mathrm{decay}}}[\mathrm{C3bBbH}_{\mathrm{host}}] - k^{+}_{\mathrm{C3bH}}[\mathrm{C3bBbC3b}_{\mathrm{host}}][\mathrm{H}] + k^{-}_{\mathrm{C3bBbH}_{\mathrm{decay}}}[\mathrm{C3bBbC3bH}_{\mathrm{host}}]$$
$$+ \frac{k^{\mathrm{FI}}_{\mathrm{catC3bH}}[\mathrm{I}][\mathrm{C3bH}_{\mathrm{fluid}}]}{K^{\mathrm{FI}}_{\mathrm{mC3bH}} + [\mathrm{C3bH}_{\mathrm{fluid}}] + [\mathrm{C3bCR1}_{\mathrm{fluid}}] + [\mathrm{iC3bCR1}_{\mathrm{fluid}}] + [\mathrm{C3bH}_{\mathrm{host}}] + [\mathrm{C3bCR1}_{\mathrm{host}}] + [\mathrm{iC3bCR1}_{\mathrm{host}}] + [\mathrm{iC3bCR1}_{\mathrm{pathogen}}] + [\mathrm{C3bCR1}_{\mathrm{pathogen}}]}$$
$$+ \frac{k^{\mathrm{FI}}_{\mathrm{catC3bH}}[\mathrm{I}][\mathrm{C3bH}_{\mathrm{host}}]}{K^{\mathrm{FI}}_{\mathrm{mC3bH}} + [\mathrm{C3bH}_{\mathrm{fluid}}] + [\mathrm{C3bCR1}_{\mathrm{fluid}}] + [\mathrm{iC3bCR1}_{\mathrm{fluid}}] + [\mathrm{C3bH}_{\mathrm{host}}] + [\mathrm{C3bCR1}_{\mathrm{host}}] + [\mathrm{iC3bCR1}_{\mathrm{host}}] + [\mathrm{iC3bCR1}_{\mathrm{pathogen}}] + [\mathrm{C3bCR1}_{\mathrm{pathogen}}]}$$

9

$$\frac{d[\text{CR1}_{\text{host}}]}{dt} = -k^{+}_{\text{CR1}}[\text{C3(H}_2\text{O)}_{\text{fluid}}][\text{CR1}] + k^{-}_{\text{CR1}}[\text{C3(H}_2\text{O)CR1}_{\text{fluid}}] - k^{+}_{\text{C3bCR1}}[\text{C3b}_{\text{fluid}}][\text{CR1}] + k^{-}_{\text{C3bCR1}}[\text{C3bCR1}_{\text{fluid}}] - k^{+}_{\text{iC3bCR1}}[\text{iC3b}_{\text{fluid}}][\text{CR1}]$$

$$+ k^{-}_{\text{iC3bCR1}}[\text{iC3bCR1}_{\text{fluid}}] - k^{+}_{\text{C3bCR1}}[\text{C3bBb}_{\text{fluid}}][\text{CR1}] + k^{-}_{\text{C3bBbCR1}_{\text{decay}}}[\text{C3bBbCR1}_{\text{fluid}}] - k^{+}_{\text{C3bCR1}}[\text{C3b}_{\text{pathogen}}][\text{CR1}] + k^{-}_{\text{C3bCR1}}[\text{C3bCR1}_{\text{pathogen}}]$$

$$- k^{+}_{\text{iC3bCR1}}[\text{iC3b}_{\text{pathogen}}][\text{CR1}] + k^{-}_{\text{iC3bCR1}}[\text{iC3bCR1}_{\text{pathogen}}] - k^{+}_{\text{C3bCR1}}[\text{C3b}_{\text{host}}][\text{CR1}] + k^{-}_{\text{C3bCR1}}[\text{C3bCR1}_{\text{host}}] - k^{+}_{\text{iC3bCR1}}[\text{iC3b}_{\text{host}}][\text{CR1}]$$

$$+ k^{-}_{\text{C3bCR1}}[\text{iC3bCR1}_{\text{host}}] - k^{+}_{\text{C3bCR1}_{\text{host}}}[\text{C3bBb}_{\text{host}}][\text{CR1}] + k^{-}_{\text{C3bBbCR1}_{\text{decay}}}[\text{C3bBbCR1}_{\text{host}}] - k^{+}_{\text{C3bCR1}}[\text{C3bBbC3b}_{\text{host}}][\text{CR1}]$$

$$+ k^{-}_{\text{C3bBbCR1}_{\text{decay}}}[\text{C3bBbC3bCR1}_{\text{host}}]$$

$$+ \frac{k^{\text{FI}}_{\text{catC3bH}}[\text{I}][\text{C3bCR1}_{\text{host}}]}{K^{\text{FI}}_{\text{mC3bH}} + [\text{C3bH}_{\text{fluid}}] + [\text{C3bCR1}_{\text{fluid}}] + [\text{iC3bCR1}_{\text{fluid}}] + [\text{C3bH}_{\text{host}}] + [\text{C3bCR1}_{\text{host}}] + [\text{iC3bCR1}_{\text{host}}] + [\text{iC3bCR1}_{\text{pathogen}}] + [\text{C3bCR1}_{\text{pathogen}}]}$$

$$+ \frac{k^{\text{FI}}_{\text{catC3bH}}[\text{I}][\text{iC3bCR1}_{\text{host}}]}{K^{\text{FI}}_{\text{mC3bH}} + [\text{C3bH}_{\text{fluid}}] + [\text{C3bCR1}_{\text{fluid}}] + [\text{iC3bCR1}_{\text{fluid}}] + [\text{C3bH}_{\text{host}}] + [\text{C3bCR1}_{\text{host}}] + [\text{iC3bCR1}_{\text{host}}] + [\text{iC3bCR1}_{\text{pathogen}}] + [\text{C3bCR1}_{\text{pathogen}}]}$$

$$+ \frac{k^{\text{FI}}_{\text{catC3bH}}[\text{I}][\text{C3bCR1}_{\text{fluid}}]}{K^{\text{FI}}_{\text{mC3bH}} + [\text{C3bH}_{\text{fluid}}] + [\text{C3bCR1}_{\text{fluid}}] + [\text{iC3bCR1}_{\text{fluid}}] + [\text{C3bH}_{\text{host}}] + [\text{C3bCR1}_{\text{host}}] + [\text{iC3bCR1}_{\text{host}}] + [\text{iC3bCR1}_{\text{pathogen}}] + [\text{C3bCR1}_{\text{pathogen}}]}$$

$$+ \frac{k^{\text{FI}}_{\text{catC3bH}}[\text{I}][\text{iC3bCR1}_{\text{fluid}}]}{K^{\text{FI}}_{\text{mC3bH}} + [\text{C3bH}_{\text{fluid}}] + [\text{C3bCR1}_{\text{fluid}}] + [\text{iC3bCR1}_{\text{fluid}}] + [\text{C3bH}_{\text{host}}] + [\text{C3bCR1}_{\text{host}}] + [\text{iC3bCR1}_{\text{host}}] + [\text{iC3bCR1}_{\text{pathogen}}] + [\text{C3bCR1}_{\text{pathogen}}]}$$

$$+ \frac{k^{\text{FI}}_{\text{catC3bH}}[\text{I}][\text{C3bCR1}_{\text{pathogen}}]}{K^{\text{FI}}_{\text{mC3bH}} + [\text{C3bH}_{\text{fluid}}] + [\text{C3bCR1}_{\text{fluid}}] + [\text{iC3bCR1}_{\text{fluid}}] + [\text{C3bH}_{\text{host}}] + [\text{C3bCR1}_{\text{host}}] + [\text{iC3bCR1}_{\text{host}}] + [\text{iC3bCR1}_{\text{pathogen}}] + [\text{C3bCR1}_{\text{pathogen}}]}$$

$$+ \frac{k^{\text{FI}}_{\text{catC3bH}}[\text{I}][\text{iC3bCR1}_{\text{pathogen}}]}{K^{\text{FI}}_{\text{mC3bH}} + [\text{C3bH}_{\text{fluid}}] + [\text{C3bCR1}_{\text{fluid}}] + [\text{iC3bCR1}_{\text{fluid}}] + [\text{C3bH}_{\text{host}}] + [\text{C3bCR1}_{\text{host}}] + [\text{iC3bCR1}_{\text{host}}] + [\text{iC3bCR1}_{\text{pathogen}}] + [\text{C3bCR1}_{\text{pathogen}}]}$$

10

$$\frac{d[\text{DAF}_{\text{host}}]}{dt} = -k^{+}_{\text{C3bBbDAF}}[\text{C3bBb}_{\text{host}}][\text{DAF}] + k^{-}_{\text{C3bBbDAF}_{\text{decay}}}[\text{C3bBbDAF}_{\text{host}}] - k^{+}_{\text{C3bBbDAF}}[\text{C3bBbC3b}_{\text{host}}][\text{DAF}]$$

$$+ k^{-}_{\text{C3bBbDAF}_{\text{decay}}}[\text{C3bBbC3bDAF}_{\text{host}}]$$

11

$$\frac{d[\text{C5}]}{dt} = -k^{+}_{\text{C3bBbC3bC5}}[\text{C3bBbC3b}_{\text{host}}][\text{C5}] + k^{-}_{\text{C3bBbC3bC5}}[\text{C3bBbC3bC5}_{\text{host}}] - k^{+}_{\text{C3bBbC3bC5}}[\text{C3bBbC3b}_{\text{pathogen}}][\text{C5}]$$

$$+ k^{-}_{\text{C3bBbC3bC5}}[\text{C3bBbC3bC5}_{\text{pathogen}}] - k^{+}_{\text{C3bBbC3bC5}}[\text{C3bBbC3bP}_{\text{pathogen}}][\text{C5}] + k^{-}_{\text{C3bBbC3bC5}}[\text{C3bBbC3bPC5}_{\text{pathogen}}]$$

$$- k^{+}_{\text{C3bBbC3bC5}}[\text{C3bBbC3bP}*_{\text{pathogen}}][\text{C5}] + k^{-}_{\text{C3bBbC3bC5}}[\text{C3bBbC3bPC5}*_{\text{pathogen}}]$$

12

$$\frac{d[\text{C5a}]}{dt} = k^{\text{kcat}}_{\text{C3bBbC3bC5}}[\text{C3bBbC3bC5}_{\text{pathogen}}] + k^{\text{kcat}}_{\text{C3bBbC3bC5}}[\text{C3bBbC3bC5}_{\text{pathogen}}] + k^{\text{kcat}}_{\text{C3bBbC3bC5}}[\text{C3bBbC3bPC5}_{\text{pathogen}}] + k^{\text{kcat}}_{\text{C3bBbC3bC5}}[\text{C3bBbC3bPC5}*_{\text{pathogen}}]$$

13

$$\frac{d[\text{C5b}]}{dt} = k^{-}_{\text{C5b}}[\text{C3bBbC3bC5b}_{\text{host}}] - k^{-}_{\text{C5b}}[\text{C3bBbC3bC5b}_{\text{pathogen}}] - k^{-}_{\text{C5b}}[\text{C3bBbC3bPC5b}_{\text{pathogen}}] - k^{-}_{\text{C5b}}[\text{C3bBbC3bPC5b}*_{\text{pathogen}}]$$

14

$$\frac{d[\text{C6}]}{dt} = -k^{+}_{\text{C3bBbC3bC5bC6}}[\text{C3bBbC3bC5b}_{\text{host}}][\text{C6}] + k^{-}_{\text{C3bBbC3bC5bC6}}[\text{C3bBbC3bC5bC6}_{\text{host}}] - k^{+}_{\text{C3bBbC3bC5bC6}}[\text{C3bBbC3bC5b}_{\text{pathogen}}][\text{C6}] + k^{-}_{\text{C3bBbC3bC5bC6}}[\text{C3bBbC3bC5bC6}_{\text{pathogen}}] - k^{+}_{\text{C3bBbC3bC5bC6}}[\text{C3bBbC3bPC5b}_{\text{pathogen}}][\text{C6}] + k^{-}_{\text{C3bBbC3bC5bC6}}[\text{C3bBbC3bC5bPC6}_{\text{pathogen}}] - k^{+}_{\text{C3bBbC3bC5bC6}}[\text{C3bBbC3bPC5b}*_{\text{pathogen}}][\text{C6}] + k^{-}_{\text{C3bBbC3bC5bC6}}[\text{C3bBbC3bC5bPC6}*_{\text{pathogen}}]$$

15

$$\frac{d[\text{C7}]}{dt} = -k^{+}_{\text{C5b7}}[\text{C3bBbC3bC5bC6}_{\text{host}}][\text{C7}] - k^{+}_{\text{C5b7}}[\text{C3bBbC3bC5bC6}_{\text{pathogen}}][\text{C7}] - k^{+}_{\text{C5b7}}[\text{C3bBbC3bPC5bC6}_{\text{pathogen}}][\text{C7}] - k^{+}_{\text{C5b7}}[\text{C3bBbC3bPC5bC6}*_{\text{pathogen}}][\text{C7}] + k^{-}_{\text{C5b7}}[\text{pC5b7}_{\text{fluid}}] + k^{-}_{\text{C5b7}}[\text{hC5b7}_{\text{fluid}}]$$

16

$$\frac{d[\text{C8}]}{dt} = -k^{+}_{\text{C5b8}}[\text{pC5b7}_{\text{fluid}}][\text{C8}] - k^{+}_{\text{C5b8}}[\text{hC5b7}_{\text{fluid}}][\text{C8}] + k^{-}_{\text{C5b8}}[\text{C5b8}_{\text{fluid}}] - k^{+}_{\text{CnC5b8}}[\text{CnC5b7}_{\text{fluid}}][\text{C8}] + k^{-}_{\text{CnC5b8}}[\text{CnC5b8}_{\text{fluid}}] - k^{+}_{\text{VnC5b8}}[\text{VnC5b7}_{\text{fluid}}][\text{C8}] + k^{-}_{\text{VnC5b8}}[\text{VnC5b8}_{\text{fluid}}] - k^{+}_{\text{C5b8host}}[\text{C5b7}_{\text{host}}][\text{C8}] - k^{+}_{\text{C5b8}}[\text{C5b7}_{\text{pathogen}}][\text{C8}]$$

17

$$\frac{d[\text{C9}]}{dt} = -k^{+}_{\text{C5b9}}[\text{C5b8}_{\text{fluid}}][\text{C9}_1] + k^{-}_{\text{C5b9}_{\text{fluid}}}[\text{C5b9}_{\text{1fluid}}] - k^{+}_{\text{CnC5b9}}[\text{CnC5b8}_{\text{fluid}}][\text{C9}] + k^{-}_{\text{CnC5b9}}[\text{CnC5b9}_{\text{fluid}}] - k^{+}_{\text{VnC5b9}}[\text{VnC5b8}_{\text{fluid}}][\text{C9}] + k^{-}_{\text{VnC5b9}}[\text{VnC5b9}_{\text{fluid}}] - k^{+}_{\text{C5b9}}[\text{C5b8}_{\text{pathogen}}][\text{C9}_{18}] - k^{+}_{\text{C5b9}}[\text{C5b8}_{\text{host}}][\text{C9}_1] - k^{+}_{\text{C5b9}}[\text{C5b9}_{\text{1host}}][\text{C9}_{17}]$$

18

$$\frac{d[\text{Vn}]}{dt} = -k^{+}_{\text{VnC5b7}}[\text{hC5b7}_{\text{fluid}}][\text{Vn}] - k^{+}_{\text{VnC5b7}}[\text{pC5b7}_{\text{fluid}}][\text{Vn}] + k^{-}_{\text{VnC5b7}}[\text{VnC5b7}_{\text{fluid}}]$$

19

$$\frac{d[\text{Cn}]}{dt} = -k^{+}_{\text{CnC5b7}}[\text{hC5b7}_{\text{fluid}}][\text{Cn}] - k^{+}_{\text{CnC5b7}}[\text{pC5b7}_{\text{fluid}}][\text{Cn}] + k^{-}_{\text{CnC5b7}}[\text{CnC5b7}_{\text{fluid}}]$$

20

$$\frac{d[\mathrm{CD59}]}{dt} = -k^{+}_{\mathrm{CD59C5b9}}[\mathrm{C5b9}_{\mathrm{1host}}][\mathrm{CD59}] + k^{-}_{\mathrm{CD59C5b9}}[\mathrm{CD59C5b9}_{\mathrm{1host}}]$$
