# Supplementary material for: Quantitative Modeling of the Alternative Pathway of the Complement System
Source: PLoS One. 2016 Mar 31;11(3):e0152337. doi: 10.1371/journal.pone.0152337 (PMC4816337; doi:10.1371/journal.pone.0152337)
Supplement: S2 Text — (PDF) [file pone.0152337.s012.pdf]

S2 Text. Modeling of Complement Amplification on Cell Surfaces.

We incorporated two types of cell surfaces in our model. Host cells are represented by human erythrocytes, and pathogen cells by *E. coli*. We made a spherical cell assumption, and used the following radii for each cell type:

$r_{erythrocyte} = 3.4 \times 10^{-6}\ m^2$

$r_{E.coli} = 0.5 \times 10^{-6}\ m^2$

Thus, the surface area of each cell type can be calculated according to

$A = 4\pi r^2$

$A_{erythrocyte} = 5.8 \times 10^{-10}\ m^2$

$A_{E.coli} = 3.14 \times 10^{-12}\ m^2$

Based on estimates from crystallographic and electron microscopy data, we assumed C3b (and C3b-containing species) to occupy a 100 × 100 Å area on cell surfaces. C5b-7 was assumed to occupy the same surface area. MAC was assumed to occupy a 200 × 200 Å area on cell surfaces, based on previously reported dimensions of the pores. The 100 × 100 Å area is referred to as one binding site, and thus MAC requires 4 binding sites for deposition. By dividing the cell surface area by binding site area, we calculated the number of binding sites per cell.

$$Number\ of\ C3b\ Binding\ Sites = \frac{A_{erythrocyte}}{A_{C3b\ Binding\ Site}} = \frac{1.45 \times 10^{-10}\ m^2}{1 \times 10^{-16}\ m^2}$$

$$= 1.45 \times 10^{6}\ sites/cell$$

Host Cell: [*C3b Binding Sites*$_{global}$] = 1.45×10^6 sites/cell × 5×10^{12} cells/L × 1 mol/6.022×10^{23} sites

= 1.20×10^{-5} M

$$Number\ of\ C3b\ Binding\ Sites = \frac{A_{E.coli}}{A_{C3b\ Binding\ Site}} = \frac{3.14 \times 10^{-12}\ m^2}{1 \times 10^{-16}\ m^2}$$

$$= 3.14 \times 10^{4}\ sites/cell$$

Pathogen: [*C3b Binding Sites*$_{global}$] = 3.14×10^4 sites/cell × 1×10^5 cells/L × 1 mol/6.022×10^{23} sites

= 5.21×10^{-15} M

These numbers represent the bulk concentration of binding sites in human blood. Since complement activation and propagation occurs primarily on cell surfaces, we adapted our model to account for the locally high concentrations of complement components and binding sites in the vicinity of surface-bound convertases. It is known that nascent C3b and C5b-7 have short half-lives before becoming inactivated and incapable of attaching to cell surfaces. Thus, we considered an active hemispheric region surrounding a surface-bound convertase, and calculated the radius of this region based on the diffusion coefficient of each species, and the time required for 90% species inactivation (according to known half-lives).

$D = \frac{k_B T}{6\pi\mu r}$, k_B = Boltzmann constant, T = temperature, μ = viscosity of blood, r = molecule radius

$r_{C3b} = 37.0\ Å$

$$D = \frac{\left(1.38 \times 10^{-23}\ \frac{kg\ m^2}{s^2\ K}\right)(310\ K)}{6\pi\left(4 \times 10^{-3}\ \frac{kg}{m\ s}\right)(3.7 \times 10^{-9}\ m)}$$

$D_{C3b} = 1.53 \times 10^{-11}\frac{m^2}{s}$, $D_{C3b} =$ diffusion coefficient of C3b

$t_{\frac{1}{2},C3b} = 60\mu s$, $t_{\frac{1}{2},C3b} =$ half – life of C3b

$t_{90\%,C3b} = 200\mu s$, $t_{90\%,C3b} =$ half – life for the 90% of C3b

$r_{Hemisphere_{C3b}} = \sqrt{6(D_{C3b})(t_{90\%,C3b})}$

$r_{Hemisphere_{C3b}} = 1356.3 \times 10^{-10} m$

$V_{Hemisphere} = \left(\frac{1}{2}\right)\frac{4}{3}\pi(r_{Hemisphere_{C3b}})^3 \times \frac{1000L}{m^3}$

$V_{Hemisphere} = 5.22 \times 10^{-18} L$

Further, the local concentration of binding sites was calculated based on the area of the hemispheric regions and the number of binding sites within the region

$A_{Hemisphere} = \pi(r_{Hemisphere_{C3b}})^2$

$A_{Hemisphere} = 5.77 \times 10^{-14}\ m^2$

$A_{C3b\ Binding\ Site} = 1 \times 10^{-16} m^2$

$$Number\ of\ C3b\ Binding\ Sites = \frac{A_{Hemisphere}}{A_{C3b\ Binding\ Site}}$$

$Number\ of\ C3b\ Binding\ Sites = 578$

$$[C3b\ Binding\ Site_{local}] = \frac{Number\ of\ C3b\ Binding\ Sites}{V_{Hemisphere}} \times \frac{1}{N_A} = 1.83 \times 10^{-4}\ \text{M}$$

$N_A =$ Avogadro's number

The effect of high local concentrations of complement components was accounted for in the equations using a scaling factor, which is multiplied by the bulk concentration of binding sites to yield an effective local concentration near the surface

$$Scaling\ Factor_{Pathogen} = \frac{[C3b\ Binding\ Site_{local}]}{[C3b\ Binding\ Site_{global}]}$$

$Scaling\ Factor_{Pathogen} = 3.52 \times 10^{10}$

$$Scaling\ Factor_{Host} = \frac{[C3b\ Binding\ Site_{local}]}{[C3b\ Binding\ Site_{global}]}$$

$Scaling\ Factor_{Host} = 15.6$

The same calculation was also done for C5b7:

$r_{C5b,7} = 47.7\ \text{Å}$

$D_{C5b7} = 1.18 \times 10^{-11} \frac{m^2}{s}$

$D_{C5b7} =$ diffusion coefficient of C5b7

$t_{\frac{1}{2},C5b7} = 0.01s, \quad t_{90\%,C5b7} = 0.033s$

$r_{Hemisphere_{C5b7}} = 15289.9 \times 10^{-10} m$

$V_{Hemisphere} = 7.63 \times 10^{-15} L$

$A_{C5b7\ Binding\ Site} = 1 \times 10^{-16} m^2$

$Number\ of\ C5b,7\ Binding\ Sites = 74410$

$[C5b7\ Binding\ Site] = 1.61 \times 10^{-5} M$

$Scaling\ Factor_{Pathogen} = 3.10 \times 10^{9}$

$Scaling\ Factor_{Host} = 1.4$

Lastly, we also estimated rate constant by using:

$$k = 4\pi R D N_A 10^3 M^{-1} s^{-1}$$

where R= radius, D = diffusion coefficient, N_A = Avogadro's number, and 10^3 is unit correction factor. All the parameter data can be found in Table S1, and concentrations can be found in Table S2.
